# Four new triterpene saponins from *Cephalaria speciosa* and their potent cytotoxic and immunomodulatory activities

**DOI:** 10.1038/s41598-023-44114-6

**Published:** 2023-10-08

**Authors:** Ozan Oztunc, Gaye Sumer Okkali, Sevda Zeinali, Ayse Nalbantsoy, Nazli Boke Sarikahya

**Affiliations:** 1 Department of Chemistry, Faculty of Science, Ege University, 35100 Bornova, Izmir Türkiye; 2 Department of Biotechnology, Faculty of Engineering, Ege University, 35100 Bornova, Izmir Türkiye; 3 Department of Bioengineering, Faculty of Engineering, Ege University, 35100 Bornova, Izmir Türkiye

**Keywords:** Natural products, Chromatography, NMR spectroscopy, Structure-based drug design

## Abstract

Four new triterpene saponins, namely speciosides A-D (**1–4**) along with six known saponins were isolated from the *n*-butanol extract of *Cephalaria speciosa*. In addition to these, three new prosapogenins (**2a–4a**) were obtained after alkaline hydrolysis. Elucidation of the structures of the isolated compounds was carried out by 1D, 2D NMR, HR-ESI/MS and GC–MS analyses. Cytotoxic activity was investigated on A549, CCD34-Lu, MDA-MB-231, PC-3, U-87MG, HeLa, HepG-2 cells by MTT method. Additionally, the immunomodulatory effect of compounds was evaluated for macrophage polarization with/without inactivated IBV D274 antigen treatment on THP-1 cells originated macrophage cells in terms of M1 or M2. According to the cytotoxicity results, compound **1** and prosapogenin **2a** exhibit significant cytotoxicity than doxorubicin by comparison. The results demonstrated that saponin molecules treated THP-1 originated macrophages were induced M1 and/or M2 polarization. Additionally, macrophage cells treated with/without IBV D274 antigen contained saponin compounds were triggered significantly M2 polarization relative to M1. Notably, monodesmosidic saponins (**1** and **2a**–**4a**) in comparison with bisdesmosidic ones (**2–4**) demonstrated the most effect on M2 polarization. In conclusion, the results showed that all the isolated new saponins and their prosapogenins have immunomodulatory potential on macrophage cells increasing immune response without significant cytotoxic effect on THP-1 originated macrophages.

## Introduction

Saponins have become the focus of attention of all scientists in recent years due to their diverse activities. Many saponin derivatives with activities on a broad scale have been reported. Biological properties such as cytotoxic, hemolytic, anti-cancer, anti-inflammatory, anti-bacterial, molluscicidal, anti-fungal, anti-viral, and insecticidal activities and different physiochemical properties of saponins have led to the vast number of traditional and industrial applications^1,2^. In the literature, triterpenoid saponins have also commercially significant usage for their pharmaceutical properties as herbal products and immunological adjuvants^3,4^. Their other applications are natural non-ionic natural surfactants in cleansing products in the personal care sector such as shower gels, shampoos, foam baths, hair conditioners, lotions, liquid soaps, baby care products, mouthwashes, and toothpastes^5,6^. Due to the broad spectrum of biological activities and diverse applications of saponins, we use *Cephalaria* species that have proven to contain large amounts of saponins based on our previous studies^7–11^. Previous phytochemical investigations on *Cephalaria* species show that this genus is rich in saponins, iridoids, flavonoids, alkaloids, and lignans^10,12–16^ and many of them have been found to possess immunomodulatory, antifungal, antioxidant, antimicrobial and cytotoxic activities^7,10,13,16^. *Cephalaria speciosa* is one of the endemic species within the genus *Cephalaria*. It is a perennial plant with light yellow flowers that grows in rocky areas around Erzincan^17^.

Macrophages not only initiate innate immune responses, but can also act as effector cells that help resolve these responses, such as fighting infection, promoting inflammation, angiogenesis, wound healing, tissue homeostasis, tissue remodeling, and the development of cancer. They possess the ability to adapt and change their genetic profile and function in response to various environmental, tissue, and inflammatory stimuli^18^. The classification of macrophages is widely accepted that they can be divided into two main subtypes with pro-inflammatory and anti-inflammatory properties known as M1-like and M2-like macrophages^19^. These subtypes represent the extremes of a spectrum with many intermediate subsets existing between them. M1 macrophages are pro-inflammatory and initiate immune responses, disrupt tissue integrity, and hinder tumor progression by enhancing anti-tumoral responses from T cells and natural killer cells. Conversely, M2 macrophages are anti-inflammatory and involved in tissue remodeling and tumor growth^20,21^. Macrophages can be stimulated by bacterial products, including lipopolysaccharides (LPS) and several inflammatory cytokines, IL-1 (interleukin-1), IL-6, IL-12, and nitric oxide (NO)^22^.

In the present paper, a study of the components of *Cephalaria speciosa* (Caprifoliaceae)^17^ has been undertaken, for the first time, and phytochemical studies on the aerial parts of the plant resulted in four new triterpene saponins (**1–4**) namely speciosides A-D, along with six known ones (**5–10**) and three new prosapogenins (**2a–4a**) obtained after alkaline hydrolysis. Their structures were established based on extensive spectroscopic analysis, including 1D, 2D NMR, and HR-ESI/MS data. Cytotoxic activity was investigated on A549, CCD34-Lu, MDA-MB-231, PC-3, U87MG, HeLa, and HepG-2 cells by MTT method. It was also examined the effect of new saponins and their prosapogenins with/without antigen treatment on macrophage cells derived from THP-1 monocytes in order to evaluate immunomodulatory potentials.

## Materials and methods

### General

In the present study, several chromatographic techniques, suitable adsorbents, solvents, and instruments were used through isolation, purification, and spectral analysis procedures. For the structural studies, FTIR spectra and optical rotations were analyzed on ATI Mattson Genesis Series Fourier transform infrared spectrophotometer and Rudolph Research Analytical Autopol I automatic polarimetry, respectively. HR-ESI/MS analyses were carried out using a Bruker LC micro-Q-TO-F mass spectrometer. GC–MS analysis for the silylated monosaccharides was performed via Shimadzu GC–MS QP 2010 plus instrument. 1D- and 2D-NMR spectra were recorded on a Varian AS 400 MHz and 600 MHz spectrometer in DMSO-*d*_6_ with TMS as an internal standard. For the plant extraction procedure, Silverson AX5 emulsifier was used. Medium Pressure Liquid Chromatography (MPLC) was carried out using a Buchi system [pump module C605; glass columns (15–49 mm × 100–920 mm); pump manager C-615; UV photometer C-635; fraction collector C-660]. Lichroprep RP-18 (25–40 µm, Merck 9303) and silica gel 60 (0.063–0.200 mm, Merck 7734) were used for both column chromatography (CC) and MPLC studies. For the open column chromatography procedures, Spectra/Chrom® CF-1 fraction collector was used to collect eluents on the same frequency. Thin layer chromatography (TLC) was performed on F254 (Merck 5554) and RP18 F254s (Merck 5560) pre-coated aluminum sheets. For evaporation procedures, Buchi vacuum evaporator instrument (rotavapor RII, vacuum controller/V-850, vacuum pump/V-700) was used to concentrate samples and extracts.

### Plant material

All methods were carried out in accordance with guidelines on good agricultural and collection practices for medicinal plants of the World Health Organization and European Medicines Agency^17,18^. The plant material was collected in accordance with relevant institutional, national and international guidelines and legislation. The appropriate permission for the collection was provided by “Republic of Turkiye Ministry of Agriculture and Forestry, General Directorate of Nature Conservation and National Parks” with the number E-21264211-288.04-6345319. *Cephalaria speciosa*^17^ was gathered in August 2013 from 1600 m altitude hillside between Sivas-Erzincan (39°54′01'' N, 38°51′08'' E). The plant was identified by R. Suleyman Gokturk and then was archived at Akdeniz University Herbarium Research Centre with the number R.S. Gokturk 7673. The aerial parts of the plant were harvested by hand, dried at room temperature and kept away sunlight.

### Extraction and isolation

Dried aerial parts of collected *Cephalaria speciosa* were grounded (2.4 kg) and extracted with MeOH (6L × 5) overnight. During the day, dried and the chopped plant was extracted via emulsifier. The filtered extract solution was evaporated under reduced pressure at 40 °C using a rotary evaporator. After this procedure, 422.0 g MeOH extract was obtained. The dried extract was further extracted with 900 mL (each) of *n*-BuOH:H_2_O (1:1) solvent system. Separated organic (301.0 g) and aqueous phases (250.3 g) were dried. For the removal of apolar components and oily parts from dried this extract, it was extracted nine times using 80 mL of *n*-hexane and 125.8 g *n*-BuOH extract was obtained. Vacuum liquid chromatography (VLC) using reversed-phase silica gel and MeOH:H_2_O solvent system with a gradient from 0 to 100% MeOH (10% MeOH increasing for each step) was used for the fractionation procedure of *n*-BuOH extract. 11 fractions were obtained after the VLC procedure and were combined as to be 4 fractions. RP-VLC fraction 3 (17.1 g) was subjected to MPLC-1 (36 × 460 mm column, max. pressure: 30 bar, flow rate: 30 mL/min, adsorbent; silica gel, solvent system; CHCl_3_:MeOH:H_2_O, 90:10:1–61:32:7) yielded 14 sub-fractions. Combined fractions Frs. 6–8 (3.2 g) of MPLC-1 was exposed to a silica gel CC eluted with CHCl_3_:MeOH:H_2_O (90:10:1–61:32:7) solvent system yielded 11 fractions. 6th fraction (341.0 mg) of this method was subjected to MPLC-2 over RP silica gel using a suitable glass column (15 × 230 mm) and programme (max. pressure: 50 bar, flow rate: 10 mL/min, solvent system; H_2_O:MeOH, 70:30–35:65) to obtain new compound **1** (42.6 mg). Fr-11 of MPLC-1 (1.6 g) was exposed to a silica gel CC eluted with CHCl_3_:MeOH:H_2_O (90:10:1–61:32:7) and 11 fractions were obtained. Fraction 6 and fraction 8 gave new compounds **4** (121.0 mg) and **7** (19.7 mg), respectively. 9th fraction from this column and the 4th fraction from MPLC-1 were combined (363.0 mg) and was subjected to a silica gel CC eluted with CHCl_3_:MeOH:H_2_O (90:10:1–61:32:7) and yielded 6 fractions. Fractions 4 and 5 gave new compounds **3** (23.0 mg) and **2** (63.0 mg), respectively. Fraction 13 of MPLC-1 (1.1 g) was subjected to MPLC-3 over RP silica gel using a suitable column (15 × 460 mm), programme (max. pressure: 50 bar, flow rate: 15 mL/min) and solvent system H_2_O:MeOH, 65:35–0:100 MeOH. Known compounds **6** (250.0 mg) and **5** (20.8 mg) were obtained from fractions 6 + 7 and 11, respectively. Hereunder, the 13th fraction of MPLC-3 (130.0 mg) was exposed to a silica gel CC eluted with CHCl_3_:MeOH:H_2_O, 90:10:1–61:32:7 to give compound **10** (36.5 mg) as a fraction 9. Fraction 12 of MPLC-1 (1.47 g) was exposed to a silica gel CC eluted with CHCl_3_:MeOH:H_2_O, 90:10:1–70:30:3, yielding 7 fractions. 6th fraction of this column (279.0 mg) was subjected to another silica gel CC eluted with CHCl_3_:MeOH:H_2_O, 90:10:1–61:32:7 to give compound **8** (36.0 mg) which is the 4th fraction of total 6 fractions. The 14th fraction of MPLC-1 (1.37 g) was exposed to a silica gel CC eluted with CHCl_3_:MeOH:H_2_O, 90:10:1–61:32:7 to give 9 fractions. Fraction 1 was detected as compound **9** (17.0 mg).

### Specioside A (1)

**Figure 1:**
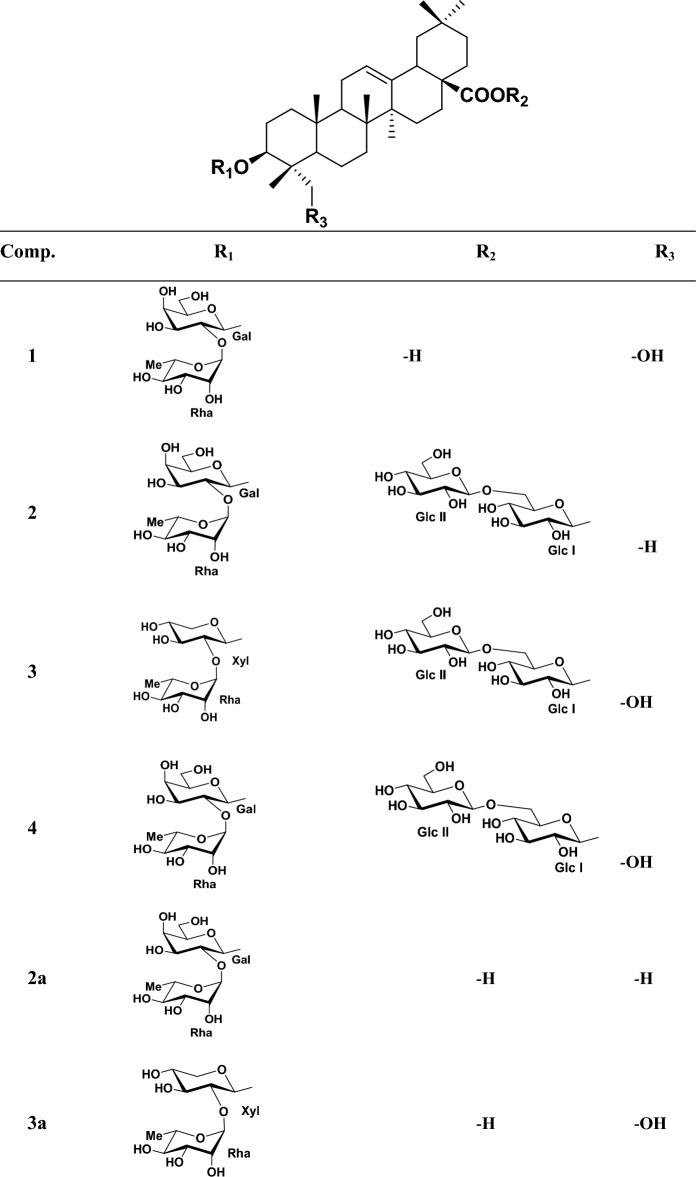

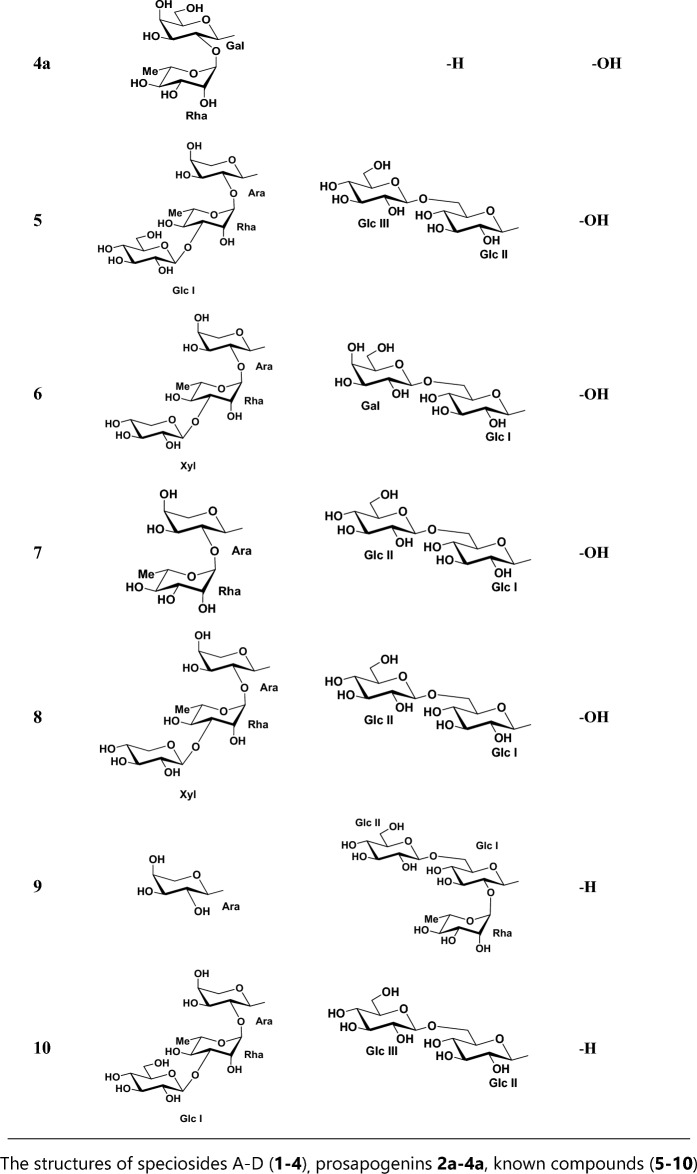
The structures of speciosides A-D (**1–4**)_**,**_ prosapogenins **2a–4a**, known compounds (**5–10**).

A pale yellow, amorphous powder (42.6 mg, 0.01% based on dry MeOH extract); [*α*]_D_^25^ -10.8 (c 0.19, DMSO); IR (ATR crystal): *ν*_max_ = 3347, 2923, 1693, 1727, 1268, 1048 cm^−1^; ^1^H-NMR (DMSO-*d*_*6*_, 600 MHz) and ^13^C-NMR (DMSO-*d*_*6*_, 150 MHz) see Tables 1 and 2, respectively; negative-ion HR-ESI/MS *m/z*: 779.45880 [M-H] (calcd. for C_42_H_67_O_13_, 779.45762). Purity of **1** is determined as 99.54% from ^1^H-NMR (Fig. 1).

**Table 1:** ^1^H-NMR data for compounds **1–4.**

No	Aglycone				No	Sugar Units			
	1	2	3	4		1	2	3	4
1	nd, 1.44, m	nd, 1.42	0.78, 1.42, m	0.79, 1.42, m		**Gal at C-3**	**Gal at C-3**	**Xyl at C-3**	**Gal at C-3**
2	1.47, 1.75, m	1.48, nd, m	1.53, 1.62, m	1.50, 1.74,m	1	4.25, d (7.2)	4.20, d (8.0)	4.26, d (7.8)	4.26, d (7.2)
3	3.47, m	2.98, m	3.46, m	3.47, m	2	3.44, m	3.50, m	3.14, m	3.46, m
4	–	–	–	–	3	3.39, m	3.42, m	3.21, m	3.43, m
5	1.16, m	nd	1.13, m	1.12, m	4	3.57, m	3.57, m	3.23, m	3.56, m
6	1.15, m, nd	nd	1.11, 1.34	1.10, 1.41,m	5	3.23, m	3.29, m	2.93, 3.64, m	3.23, m
7	1.14, 1.46, m	0.63, m, nd,	1.10, m, nd	nd, 1.14, m	6	3.38, 3.49, m	3.43, 3.62, m		3.39, 3.61, m
8	–	–	–	–		**Rha**	**Rha**	**Rha**	**Rha**
9	1.47, m	1.43, m	1.45, m	1.42, m	1	5.17, brs	5.20, brs	5.19, brs	5.17, brs
10	–	–	–	–	2	3.67, m	3.68, m	3.68, m	3.67, m
11	1.05, 1.75, m	nd, 1.75, m	1.05, 1.75, m	nd, 1.75, m	3	3.47, m	3.47, m	3.45, m	3.45, m
12	5.10, brs	5.14, brs	5.13, brs	5.14, brs	4	3.14, m	3.16, m	3.14, m	3.11, m
13	–	–	–	–	5	3.77, m	3.79, m	3.73, m	3.77, m
14	–	–	–	–	6	1.01, m	1.03, d (6.0)	1.02, d (6.0)	1.03, d (6.4)
15	nd	0.91, m, nd	0.92, 1.69, m	0.90, 1.73, m			**Glc I at C-28**		
16	nd	nd, 1.91, m	1.55, 1.92, m	1.56, 1.95, m	1		5.21, d (6.4)	5.18, d (8.4)	5.20, d (8.4)
17	–	–	–	–	2		3.10, m	3.09, m	3.10, m
18	2.73, dd (9; 2.2)	2.71, m	2.68, m	2.70, m	3		3.33, m	3.31, m	3.36, m
19	1.01, 1.57, m	nd, 1.58, m	1.04, 1.58, m	1.03, 1.55, m	4		3.19, m	3.18, m	3.16, m
20	–	–	–	–	5		3.19, m	3.16, m	3.17, m
21	1.09, 1.26, m	nd	1.13, 1.31, m	1.11, 1.33, m	6		3.57, 3.89, m	3.56, m, 3.90, d (7.2)	3.56, 3.90, m
22	1.38, 1.56, m	nd	1.45, m	1.45, m			**Glc II**		
23	3.05, 3.34, m	0.93, s	3.05, 3.29, m	3.06, 3.34, m	1		4.17, d (7.2)	4.17, d (7.8)	4.18, d (8.0)
24	0.53, s	0.72, s	0.53, s	0.53, s	2		2.92, m	2.91, m	2.93, m
25	0.83, s	0.89, s	0.82, s	0.84, s	3		3.08, m	3.10, m	3.07, m
26	0.68, s	0.66, s	0.64, s	0.63, s	4		3.18, m	3.03, m	3.01, m
27	1.06, s	1.04, s	1.04, s	1.04, s	5		3.02, m	2.99, m	3.01, m
28	–	–	–	–	6		3.39, 3.50, m	3.41, 3.59, m	3.37, 3.58, m
29	0.83, s	0.83, s	0.83, s	0.84, s					
30	0.84, s	0.82, s	0.83, s	0.84, s					

^1^H NMR data (*δ*) were measured in DMSO-*d*_6_ at 600 MHz.

Coupling constants (*J*) in Hz are given in parentheses.

The assignments are based on DEPT, COSY, HMQC and HMBC experiments.

nd: not detected.

**Table 2:** ^13^C-NMR data for compounds **1–4.**

No	1	2	3	4	No	1	2	3	4
1	38.8	38.9	38.7	38.9		**Gal at C-3**	**Gal at C-3**	**Xyl at C-3**	**Gal at C-3**
2	25.9	26.3	25.7	25.9	1	103.8	104.8	104.0	103.8
3	80.0	88.4	79.5	80.0	2	74.3	74.6	76.6	74.4
4	42.6	39.4	42.6	42.6	3	75.5	75.3	78.5	75.4
5	46.8	55.8	46.6	46.8	4	69.0	69.3	70.5	69.3
6	17.6	18.2	nd	17.5	5	75.2	75.1	66.0	75.2
7	32.9	32.7	32.2	32.3	6	60.5	61.4		61.4
8	39.2	39.0	39.3	39.4		**Rha**	**Rha**	**Rha**	**Rha**
9	47.7	47.6	47.5	47.6	1	100.2	100.4	100.3	100.3
10	36.4	36.7	36.4	36.4	2	70.7	70.8	70.7	70.7
11	23.4	23.4	23.4	23.4	3	70.9	70.8	70.8	70.9
12	121.1	122.1	122.1	122.1	4	72.5	72.5	72.5	72.6
13	145.4	144.0	144.0	144.0	5	68.1	68.4	68.3	68.3
14	41.8	41.7	41.7	41.7	6	18.3	18.3	18.2	18.2
15	27.8	27.6	27.6	27.7			**Glc I at C-28**		
16	23.5	22.9	22.9	22.9	1		94.5	94.5	94.5
17	46.8	46.4	46.4	46.4	2		72.7	72.7	72.7
18	41.7	41.1	41.1	41.1	3		76.9	77.1	76.9
19	46.0	46.0	46.0	46.0	4		69.7	69.5	69.7
20	31.0	30.7	30.7	30.7	5		77.1	76.9	77.1
21	34.3	33.7	33.6	33.7	6		68.2	68.1	68.3
22	32.5	32.1	32.1	32.1			**Glc II**		
23	62.8	27.8	62.6	62.8	1		103.4	103.4	103.4
24	13.4	16.7	13.5	13.5	2		73.9	73.9	73.9
25	16.0	15.7	16.1	16.1	3		77.2	77.1	77.1
26	17.5	17.1	17.1	17.2	4		70.4	70.3	70.4
27	26.1	26.0	26.0	26.0	5		77.2	77.3	77.2
28	180.8	175.7	175.7	175.7	6		60.6	61.3	60.7
29	33.6	33.2	33.2	33.2					
30	24.0	23.8	23.9	23.9					

^13^C NMR data were measured in DMSO-*d*_6_ at 150 MHz.

The assignments are based on HSQC, COSY and HMBC experiments.

nd: not detected.

### Specioside B (2)

A white, amorphous powder (63.0 mg, 0.015% based on dry MeOH extract); [*α*]_D_^25^ -6.1 (c 0.33, MeOH); IR (ATR crystal): *ν*_max_ = 3350, 2927, 1634, 1738, 1454, 1047 cm^−1^; ^1^H-NMR (DMSO-*d*_*6*_, 600 MHz) and ^13^C-NMR (DMSO-*d*_*6*_, 150 MHz) see Tables 1 and 2, respectively; positive-ion HR-ESI/MS *m/z*: 1111.56523 [M + Na]^+^ (calcd. for C_54_H_88_O_22_Na, 1111.56595. Purity of **2** is determined as 96.25% from ^1^H-NMR (Fig. 1).

### Specioside C (3)

A white, amorphous powder (23.0 mg, 0.005% based on dry MeOH extract); [*α*]_D_^25^ -15.9 (c 0.13, MeOH); IR (ATR crystal): *ν*_max_ = 3328, 2923, 1634, 1731, 1404, 1032 cm^−1^; ^1^H-NMR (DMSO-*d*_*6*_, 600 MHz) and ^13^C-NMR (DMSO-*d*_*6*_, 150 MHz) see Tables 1 and 2, respectively; positive-ion HR-ESI/MS *m/z*: 1097.54704 [M + H]^+^ (calcd. for C_53_H_86_O_22_, 1097.55030). Purity of **3** is determined as 96.72% from ^1^H-NMR (Fig. 1).

### Specioside D (4)

A white, amorphous powder (121.0 mg, 0.03% based on dry MeOH extract ); [*α*]_D_^25^ -11.7 (c 0.34, MeOH); IR (ATR crystal): *ν*_max_ = 3381, 2926, 1737, 1634, 1056 cm^−1^; ^1^H-NMR (DMSO-*d*_*6*_, 600 MHz) and ^13^C-NMR (DMSO-*d*_*6*_, 150 MHz) see Tables 1 and 2, respectively; positive-ion HR-ESI/MS *m/z*: 1127.55725 [M + H]^+^ (calcd. for C_54_H_88_O_23_, 1127.56086). Purity of **4** is determined as 99.86% from ^1^H-NMR (Fig. 1).

### Monosaccharide analysis

For the investigation of the exact structure of carbohydrate units, acidic hydrolysis and GC–MS analysis were performed. 3–5 mg (each) of compounds **1–4** were hydrolyzed with 5 ml 5% HCl (aq) solution under reflux for 2 h at 90 °C. And then the reaction mixture was neutralized using a 5% KOH (aq) solution. The resulting aqueous solution was extracted with 10 ml of CHCl_3_, the extraction procedure was repeated three times for the organic and aqueous layers from each extraction step. The water phase dried and was kept in a *hi-vac* for 24 h. It was dissolved in 2 mL of pyridine, after dissolution 2 mL of HMDS-TMCS (1:1) (hexamethyldisilazane-trimethylchlorosilane) was added and was carried out under reflux, and argon gas at 85 °C for 2 h. A silylation reaction was carried out for the standard sugar mixture, simultaneously. The monosaccharide analysis of the compounds **1–4** was performed by the GC-Mass method of Sarikahya and Kirmizigul^10^ under the following conditions; column, HP-5MS (60 m × 0.25 mm ID × 0.25 µm df), flow rate, 1.0 mL/min; split ratio, 1:20; ion source temperature, 230 °C; inlet and interface temperatures, 250 °C; temperature program started at 60 °C (held for 1 min) followed by 10 °C/min at 240 °C (held for 2 min), the final temperature reached 320 °C (held for 3 min) and the EI mode was used. L-rhamnose, D-mannose, D-xylose, D-galactose, and D-glucose were detected by co-injection of the hydrolysate with standard silylated sugars peaks at Rt (min) 13.38, 15.39, 14.51, 15.93 and 17.13, respectively.

### Alkaline hydrolysis

The esteric linkages of the sugar moieties which are in bisdesmosidic triterpene glycosides (compounds **2–4**) were hydrolyzed by the following method. In this method, all glycosides (3 mg each) were refluxed with 5% KOH in a water solution (pH = 12–13) at 80 °C for 1 h. The reaction mixtures were neutralized with 5% HCl in a water solution and then concentrated to dryness^10^. The residues were extracted with *n*-BuOH:H_2_O (1:1) and the organic layers were analyzed by ^1^H-NMR to give the pure prosapogenins (**2a–4a**); 3-O-*α*-L-rhamopyranosyl-(1 → 2)-*β*-D-galactopyranosyl oleanoic acid (**2a**), 3-O-*α*-L-rhamopyranosyl-(1 → 2)-*β*-D-xylopyranosyl hederagenin (**3a**), 3-O-*α*-L-rhamopyranosyl-(1 → 2)-*β*-D-galactopyranosyl hederagenin (**4a**).

### Cell culture

The human leukemia monocyte cell line (THP-1), human lung adenocarcinoma cell line (A549), human breast cancer cells (MDA-MB231), human prostate adenocarcinoma cells (PC3), human malignant glioma cell line (U87MG), human cervical gland carcinoma cells (HeLa), human hepatocellular carcinoma cells (HEP-G2) and normal human lung fibroblast cells (CCD-34-LU) as a healthy cell line in our stock used in this study were purchased from American Type Culture Collection (ATCC). THP-1 cells were cultured in Roswell Park Memorial Institute (RPMI 1640) medium and MDA-MB231, PC3, U87 MG, HeLa, HEP-G2, and CCD-34-LU cells cultivated in Dulbecco’s Modified Eagle’s Medium/Nutrient Mixture F-12 (DMEM/F12) (Gibco, USA) medium supplemented with 10% FBS (Gibco, USA), 100U/mL of penicillin 100 µg/mL of streptomycin (Gibco, USA), and 2 mM L-glutamine (Gibco, USA).

### Cytotoxicity analysis of new compounds 1–4 and 2a–4a

Cytotoxicity of saponins (**1–4**) and their prosapogenins (**2a–4a**) were tested against A549, MDA-MB-231, PC-3, U87MG, HeLa, HepG-2 cancer cells and CCD34-Lu healthy cell line using MTT [3-(4,5-dimethylthiazole-2-yl)-2,5-diphenyltetrazolium bromide)] assay (Acros Organics, New Jersey, USA)^11, 23^. For this purpose, 1 × 10^5^ cells/mL of all the cell lines were seeded in 96-well plates (Corning, USA) and cultivated in the at 37 °C overnight. After the incubation, the cells were incubated with different concentrations of saponins (50, 5, and 0.5 µg/mL), and doxorubicin (Sigma, USA) (20, 2, and 0.2 µg/mL) for 48 h at 37 °C. Then, the MTT was added as 20 *μ*L (from the stock concentration of 2.5 mg/mL) to all wells and further incubated for 4 h at 37 °C. Following the incubation, the medium was removed and 150 µL DMSO was added to dissolve the formed formazan crystals. After the formazan crystals were completely dissolved, the optical density (OD) was measured at 570 nm with an UV/Vis microplate spectrophotometer (Thermo Fisher Scientific, USA). All data are the results of at least three independent experiments carried out in triplicate. Data are presented as mean ± standard error of the mean (SEM) of samples. The IC_50_ value was calculated using the GraphPad Prism 8 (USA) program based on the calculated percent viability values and absorbance values. The percentage of viability was determined by Eq. (1):

1 $$\% {\text{Viable}}\,{\text{cells}} = \frac{{ \left[ {\left( {{\text{absorbance}}\,{\text{of}}\,{\text{treated}}\,{\text{cells}}} \right) - \left( {{\text{absorbance}}\,{\text{of}}\,{\text{blank}}} \right)} \right] }}{{\left[ {\left( {{\text{absorbance}}\,{\text{of}}\,{\text{control}}} \right) - \left( {{\text{absorbance}}\,{\text{of}}\,{\text{blank}}} \right)} \right] }} \times 100$$

### Determination of macrophage polarization by flow cytometry

To differentiate THP-1 cells into M0-THP-1, cells were seeded at an initial concentration of 1 × 10^6^ cells/mL in 6-well plates (Corning, USA) and treated with 15 ng/mL phorbol 12-myristate 13—acetate (PMA, Sigma USA). Then incubate at 5% CO_2_, 37 °C for 48 h^24^. To evaluate the potential of saponins with/without infectious bronchitis virus (IBV) D274 antigen on macrophage polarization, THP-1 cells were treated with saponins at 3 µg/mL and then incubated for 24 h with or without 4 µL of inactivated IBV D274 antigens (final titer of 1 HA). The inactive IBV D274 antigen used in this study was prepared in our previously published study^25^. Based on the results of our previous research, the saponin concentration was selected as a non-cytotoxic concentration^9^. At the end of the incubation period, the cells were collected by trypsinization and washed three times with PBS. Following, 1 µL of PE anti-human CD80 (Biolegend, CA), FITC anti-human CD163 (Biolegend, CA), and FITC anti-human CD11b (Biolegend, CA) antibodies were added into the cells pellet and incubated for 45 min at room temperature in the dark. The cells pellets underwent two PBS washes following incubation. Finally, the cells were subsequently analyzed by flow cytometry (BD Accuri, C5). 1 × 10^4^ cells were counted for each sample group using flow cytometry. All data are the results of at least three independent experiments carried out in triplicate. Data are presented as mean ± standard error of the mean (SEM) of samples. The untreated stained macrophage cells with anti-human CD80, FITC anti-human CD163, and FITC anti-human CD11b were compared with the results of treated cells to obtain from M0 polarization to M1 and M2 or both.

### Statistical analysis

The results of at least three independent experiments were compared with the variable groups and the control group, with mean ± standard deviation values. One-way analysis of variance (ANOVA) was performed with GraphPad Prism 8 (USA) by Dunnett’s test. *P* < 0.05 was considered significant.

## Results and discussion

Phytochemical studies on the aerial parts of the *Cephalaria speciosa* resulted in a total of 10 saponins, 4 of which new triterpene saponins, namely, speciosides A-D (**1–4**) with 3 new prosapogenins (**2a–4a**) (Fig. 1). A total of 6 known saponins named macranthoidin A (**5**)^26^ elmalienoside A (**6**)^27^, dipsacoside B (**7**)^28^, decaisoside E (**8**)^29^, scoposide A (**9**)^10^ (3-O-*β*-D-glucopyranosyl-(1 → 3)-*α*-L-rhamnopyranosyl-(1 → 2)-*α*-L-arabinopyranosyl oleanolic acid 28-*β*-D-glucopyranosyl-(1 → 6)-*β*-D-glucopyranosyl ester (**10**)^30^ were also isolated, purified and structurally identified.

Compound **1** was obtained as a pale yellow amorphous powder having [α]_D_^25^: –10.8 (c: 0.19, DMSO). IR spectrum of compound **1** showed absorption bands of functional groups which are aliphatic C–H (2923 cm^−1^), alkene –C=C– (1693 cm^−1^), carboxylic acid carbonyl C=O (1727 cm^−1^), and C–O (1268, 1048 cm^−1^) and hydroxyl groups (3347 cm^−1^). The ^13^C-NMR spectrum showed that compound **1** consisted of 42 carbons (Table 2), of which 12 were identified for carbohydrate groups and 30 carbons were determined for the aglycone. The ^13^C-NMR data of **1** revealed the presence of six methyl carbon signals at *δ*_C_ 13.4 (C-24), 16.0 (C-25), 17.5 (C-26), 26.1 (C-27), 33.6 (C-29), 24.0 (C-30) and ^1^H-NMR data showed six singlet proton signals at *δ*_H_ 0.53, 0.83, 0.68, 1.06, 0.83, 0.84, respectively. The aglycone part of **1** was also found to consist of one carboxylic acid carbon at *δ*_C_ 180.8 (C-28), oxygen-bearing methine carbon at *δ*_C_ 80.0 (C-3), hydroxyl methyl group at *δ*_C_ 62.8 (C-23), and with two typical olefinic carbon signals at *δ*_C_ 121.1 (C-12) and 145.4 (C-13) confirmed that compound **1** has a hederagenin aglycone. The C-28 carbonyl carbon was observed at 180.8 which suggests that **1** is a monodesmosidic triterpene saponin. The 2D-NMR correlations for the aglycone moiety were detected as follows, between *δ*_H_ 0.68 proton signal (H-26) and *δ*_C_ 32.9 (C-7), 41.8 (C-14), 47.7 (C-9) carbon signals, *δ*_H_ 0.53 (H-24) singlet methyl proton signal and *δ*_C_ 42.6 (C-4), 46.8 (C-5), 62.8 (C-23), 80.0 (C-3) carbon signals, *δ*_H_ 0.83 proton signal of H-25 and *δ*_C_ 36.4 (C-10), 38.8 (C-1), 46.8 (C-5) carbon signals, *δ*_H_ 0.83 proton signal of H-30 and *δ*_C_ 31.0 (C-20), 33.6 (C-29) carbon signals, proton of C-29 *δ*_H_ 0.83 and *δ*_C_ 24.0 (C-30) carbon signal of aglycone. Another correlation was observed between *δ*_H_ 1.06 proton signal of H-27 of aglycone and *δ*_C_ 27.8 (C-15), 39.2 (C-8), 41.8 (C-14). The carbon signal of C-13 of aglycone, *δ*_C_ 145.4 (C-13) gives HMBC correlation with both proton signals *δ*_H_ 1.05 and 1.75 of H-11. HMBC correlations were also observed between H-23 protons (*δ*_H_ 3.34, 3.05) and C-3 (*δ*_C_ 80.0), C-24 (*δ*_C_13.4) carbon signals, H-18 proton *δ*_H_ 2.73 and C12-13 (*δ*_C_ 122.1, 145.4) carbon signals, H-12 proton (*δ*_H_ 5.10) and C-14 (*δ*_C_ 41.8) carbon signal. In addition to these, the COSY spectrum supported these correlations. For the carbohydrate moieties, *δ*_C_ 103.8 and 100.2 due to the high chemical shift values identified as anomeric carbons of sugars. *δ*_C_ 18.3 peak in ^13^C-NMR was identified as a typical signal for C-6 of rhamnose. Carbon signals at *δ*_C_ 103.8, 74.3, 75.5, 69.0, 75.2, 60.5 and *δ*_C_ 100.2, 70.7, 70.9, 72.5, 68.1, 18.3 were identified as two different carbohydrate units depending on COSY and HMBC spectra. Sugar moiety where directly bonded to aglycone was determined by HMBC correlation between *δ*_C_ 80.0 (C-3) and *δ*_H_ 4.25 (anomeric proton of galactose). Rhamnose and galactose linkage point was also identified by HMBC correlation between *δ*_C_ 100.2 (C-1 of rhamnose) and *δ*_H_ 3.44 (H-2 of galactose). All other 2D-NMR correlations supported carbon and proton values of the two carbohydrate moieties. A gas chromatography analysis of **1** also confirmed the types of sugar units. Identification of L-rhamnose and D-galactose was detected for **1**, giving peaks at 13.35 min and 15.89 min, respectively. Based on all these evidences IUPAC name of compound **1** is 3-O-*α*-L-rhamopyranosyl-(1 → 2)-*β*-D-galactopyranosyl-hederagenin (specioside A) (Fig. 1) (Fig. S 1–S 8 in Sup. Info).

Compound** 2** was obtained as a white amorphous powder with specific rotation of [α]_D_^25^: –6.1 (c 0.33, MeOH). The IR and NMR spectroscopic features of **2** were similar to those of **1** except for the sugar units and free typical hydroxy group linked C-23 (Tables 1 and 2). Spectral analysis of the compound **2** showed that the structure consists of 54 carbons can be seen ^13^C-NMR spectrum (See. Sup. Info. Fig. S 13). Aglycone part was determined as oleanoic acid based on chemical shift difference for C-23 of aglycone compared to hederagenin where compound **2** shows signal for C-23 at *δ*_C_ 27.8 methyl signal (Table 2). The structure of aglycone moiety was defined based on HSQC, COSY, and HMBC spectrum analysis in a similar way as in compound **1**. The ^1^H and ^13^C-NMR data for the aglycone part also agree with data for oleanoic acid aglycone reported in the literature^10^. Sugar carbons were defined according to COSY and HMBC correlations. For the sugar moieties, the ^1^H-NMR spectrum of compound **2** showed four anomeric proton signals at *δ*_H_ 4.20 (d, *J* = 8.0 Hz), 5.20 (brs), 5.21 (d, *J* = 6.4 Hz), 4.17 (d, *J* = 7.2 Hz), giving in the HSQC spectrum cross-peaks with four anomeric carbon signals at *δ*_C_ 104.8, 100.4, 94.5 and 103.4, respectively. HMBC correlations between *δ*_H_ 4.20 (d, *J* = 8.0 Hz) (H-1 of galactose) and *δ*_C_ 88.4 (C-3 of the aglycone), *δ*_H_ 5.20 (brs) (H-1 of rhamnose) and *δ*_C_ 74.6 (C-2 of galactose), *δ*_H_ 5.21 (d, *J* = 6.4 Hz) (H-1 of Glc I) and *δ*_C_ 175.7 (C-28 of aglycone), *δ*_H_ 4.17 (d, *J* = 7.2 Hz) (H-1 of Glc II) and *δ*_C_ 68.2 (C-6 of Glc I) showed the linkage points of the sugar molecules. The sugars in the structure were also proven by GC–MS analysis. Identification of L-rhamnose, D-galactose, and D-glucose was detected for **2**, giving from peaks at 13.35, 15.89, and 17.09 min, respectively. Accordingly, the structure of **2** was assigned as 3-O-*α*-L-rhamnopyranosyl-(1 → 2)-*β*-D-galactopyranosyl-oleanoic acid-28-O-*β*-D-glucopyranosyl-(1 → 6)-*β*-D-glucopyranosyl ester (specioside B) (Fig. 1) (Fig. S 9–S 16 in Sup. Info.).

Compound **3** was obtained as a white amorphous powder with a specific rotation of [α]_D_^25^ –15.9 (c 0.13, MeOH). The IR and NMR data of the aglycon moiety of **3** were identical to the other new compounds (**1** and **4**). The C-3 oxymethine carbon and C-28 carbonyl carbon were observed at *δ*_C_ 79.5 and 175.7, respectively which suggests that **3** is a bisdesmosidic triterpene saponin. The other aglycone carbon locations were determined by studies on COSY and HMBC correlations. For the sugar moieties, the ^1^H-NMR spectrum of **3** displayed four anomeric proton signals at *δ*_H_ 4.26 (d, *J* = 7.8 Hz), 5.19 (brs), 5.18 (d, *J* = 8.4 Hz) and 4.17 (d, *J* = 7.8 Hz) giving in the HSQC spectrum cross-peaks with four anomeric carbon signals at *δ*_C_ 104.0, 100.3, 94.5 and 103.4, respectively. Proton signals for the sugar moieties were associated with COSY and HMBC spectra. Carbohydrate bonding points to aglycone were confirmed by HMBC correlations between *δ*_C_ 79.5 (C-3 of aglycone) and *δ*_H_ 4.26 (H-1 of xylose) and *δ*_C_ 175.7 (C-28 of aglycone) and *δ*_H_ 5.18 (H-1 of Glc I). The interactions between *δ*_C_ 76.6 (C-2 of xylose) and *δ*_H_ 5.19 (H-1 of rhamnose), *δ*_H_ 4.17 (H-1 of Glc II), and *δ*_C_ 68.1 (C-6 of Glc I) showed the bonding points of the sugar molecules to one another. Identification of L-rhamnose, D-xylose, and D-glucose in the GC–MS experiment was detected for **3**, giving from peaks at 13.34, 14.58, and 17.09 min respectively, and thus the sugar types were clarified. Based on all this evidence IUPAC name of compound **3** is 3-O-*α*-L-rhamnopyranosyl-(1 → 2)-*β*-D-xylopyranosyl-hederagenin-28-O-*β*-D-glucopyranosyl-(1 → 6)-*β*-D-glucopyranosyl ester (specioside C) (Fig. 1) (Fig. S 17–S 24 in Sup. Info.).

Compound **4** was obtained as a pale yellow amorphous powder with a specific rotation of [α]_D_^25^ –11.7 (c 0.34, MeOH). Compound **4** consists of 54 carbons according to ^13^C-NMR (Table 2). 24 of the carbon signals were identified for carbohydrate groups and 30 carbons were determined for the aglycone and confirmed that compound **4** has a hederagenin aglycone. The C-3 oxymethine carbon and C-28 carbonyl carbon were observed at *δ*_C_ 80.0 and 175.7, respectively which suggests that compound **4** is a bisdesmosidic hederagenin type triterpene saponin. Aglycone carbon locations were determined by studies on COSY and HMBC correlations. For the carbohydrate moieties, the ^1^H-NMR spectrum of compound **4** showed four anomeric proton signals at *δ*_H_ 4.26 (d, *J* = 7.2 Hz), 5.17 (brs), 5.20 (d, *J* = 8.4 Hz) and 4.18 (d, *J* = 8.0 Hz) giving HSQC spectrum cross-peaks with four anomeric carbon signals at *δ*_C_ 103.8, 100.3, 94.5 and 103.4, respectively. HMBC cross-peaks between *δ*_C_ 80.0 (C-3 of aglycone) and *δ*_H_ 4.26 (H-1 of galactose), *δ*_C_ 175.7 (C-28 of aglycone) and *δ*_H_ 5.20 (H-1 of Glc I) showed the sugar linkage points to the aglycone. Furthermore, HMBC correlations between *δ*_H_ 5.17 (H-1 of rhamnose) and *δ*_C_ 74.4 (C-2 of galactose), *δ*_H_ 3.56, 3.90 (both H-6 of Glc I) and *δ*_C_ 103.4 (C-1 of Glc II) showed sugar to sugar bonding points. Identification of L-rhamnose, D-galactose, and D-glucose in GC–MS analysis was detected for **4**, giving from peaks at 13.35, 15.89, and 17.09 min, respectively. Based on all this evidence IUPAC name of compound **4** is 3-O-*α*-L-rhamnopyranosyl-(1 → 2)-*β*-D-galactopyranosyl-hederagenin-28-O-*β*-D-glucopyranosyl-(1 → 6)-*β*-D-glucopyranosyl ester (specioside D) (Fig. 1) (Fig. S 25–S 41 in Sup. Info.). All other structures and ^1^H and ^13^C spectra of known compounds were given in Sup. Info (Fig. S 42–S 59).

The cytotoxic effects of compounds **1–4** and prosapogenins **2a–4a**, which was obtained after alkaline hydrolysis, were examined using the MTT method. The estimated IC_50_ values was given in Table 3 and the percent vitality graph according to the MTT result was given in Fig. S 60–S 67 in Sup. Info. According to the results, while compounds **2–4**, and **3a** did not have a cytotoxic effect on THP-1-derived macrophage cells., compounds **1**, **2a**, and **4a** (27.57 ± 0.52, 37.34 ± 8.17, and 35.74 ± 4.90 µM) inflicted weak cytotoxic effect when compared with doxorubicin (6.84 ± 0.18 µM). Compounds **1**, **2a**, and **4a** demonstrated significant cytotoxicity on most cancerous cells. The mentioned compounds showed significant IC_50_ on A549 and MDA-MB-231 cells as 8.59 ± 0.19 and 15.09 ± 1.02 µM for compound **1**, and 20.77 ± 0.46 and 24.29 ± 2.65 µM for prosapogenin **2a**, and 8.13 ± 0.01 and 8.43 ± 0.15 µM for **4a** when compared with doxorubicin (40.01 ± 0.02 and 70.00 ± 0.02 µM). Compounds **2–4** and prosapogenin **3a** did not show significant cytotoxicity on all the tested cell lines (Table 3). These results demonstrated that bisdesmosidic compounds (**2–4**) did not show any cytotoxic activity while following monodesmosidic compounds (**1**, **2a**, **3a**, and **4a**) have cytotoxic effect on cancerous cell lines. In literature, as a similar study Tlili et al.^31^, who identified the biochemical profiles of the major compounds found in plant leaf extract and examined its anti-leukemic potential against acute monocytic leukemia (AML) THP-1 cells. They proved that the mTOR pathway may be involved in cell cycle inhibition and apoptosis induction caused by saponin. Furthermore, the obtained data confirm the predictions that the monodesmosidic saponins exhibit parallel cytotoxicity in our previous studies^9,11^. Furthermore, the structures of compounds **1** and **2a** have the same galactose and rhamnose units attached third carbon of aglycone moieties however their aglycone structures are hederagenin and oleanane, respectively. These structural varieties may prove the activity distinctness; cytotoxic activity on A549, MDA-MB-231, and HeLa cell lines (8.59 ± 0.19, 15.09 ± 1.02, and 48.11 ± 4.56 µM) for compound **1**, and A549 and MDA-MB-231 (20.77 ± 0.46 and 24.29 ± 2.65 µM) for compound **2a**. These results may relate with free -CH_2_OH of hederagenin aglycone moiety of compound **1** increased the cytotoxicity.

**Table 3:** The IC_50_ values for compounds **1–4** and **2a–4a** and doxorubicin (*μ*M).

Samples	THP-1 originated-macrophage	CCD-34Lu	A549	MDA-MB-231	PC-3	U87MG	HeLa	HepG-2
1	27.57 ± 0.52	–	8.59 ± 0.19	15.09 ± 1.02	–	–	48.11 ± 4.56	–
2	–	–	–	–	–	–	–	–
3	–	–	–	–	–	–	–	–
4	–	–	–	–	–	–	–	–
2a	37.34 ± 8.17	–	20.77 ± 0.46	24.29 ± 2.65	–	–	–	–
3a	–	*–*	–	–	–	–	–	–
4a	35.74 ± 4.90	–	8.13 ± 0.01	8.43 ± 0.15	–	–	16.33 ± 4.75	–
Doxorubicin	6.84 ± 0.18	33.98 ± 0.02	40.01 ± 0.02	70.00 ± 0.02	4.23 ± 0.02	11.09 ± 0.02	23.95 ± 0.02	36.85 ± 0.02

–, Not detected.

All values were represented the mean ± standard deviation (n = 3 test).

In this study, it was also aimed to examine the potential of saponin and their prosapogenins from *C. speciosa* on THP-1 monocytes-originated macrophage polarization. Regarding the immune modulation potential of saponins, THP-1-originated macrophage cells were used to determine of saponin effect on macrophage polarization. The use of the THP-1 cell line as a suitable model to study the functions and responses of monocytes and macrophages, as well as the differentiation of macrophages and any potential effects of environmental stimuli is suggested^22^. Also, Genin et al.^32^ suggested the THP-1 cell line for macrophage differentiation and established a novel and practical model of human macrophage polarization to study how macrophages could modulate tumor cells, specifically the tumor cells' response to chemotherapeutic agents. For this purpose, THP-1 cells were used as a suitable model to study macrophage differentiation and the potential effects of environmental stimuli. The THP-1 cell line is recommended for macrophage differentiation and for establishing a practical model of human macrophage polarization to study how macrophages could modulate tumor cells.

**Figure 2:**
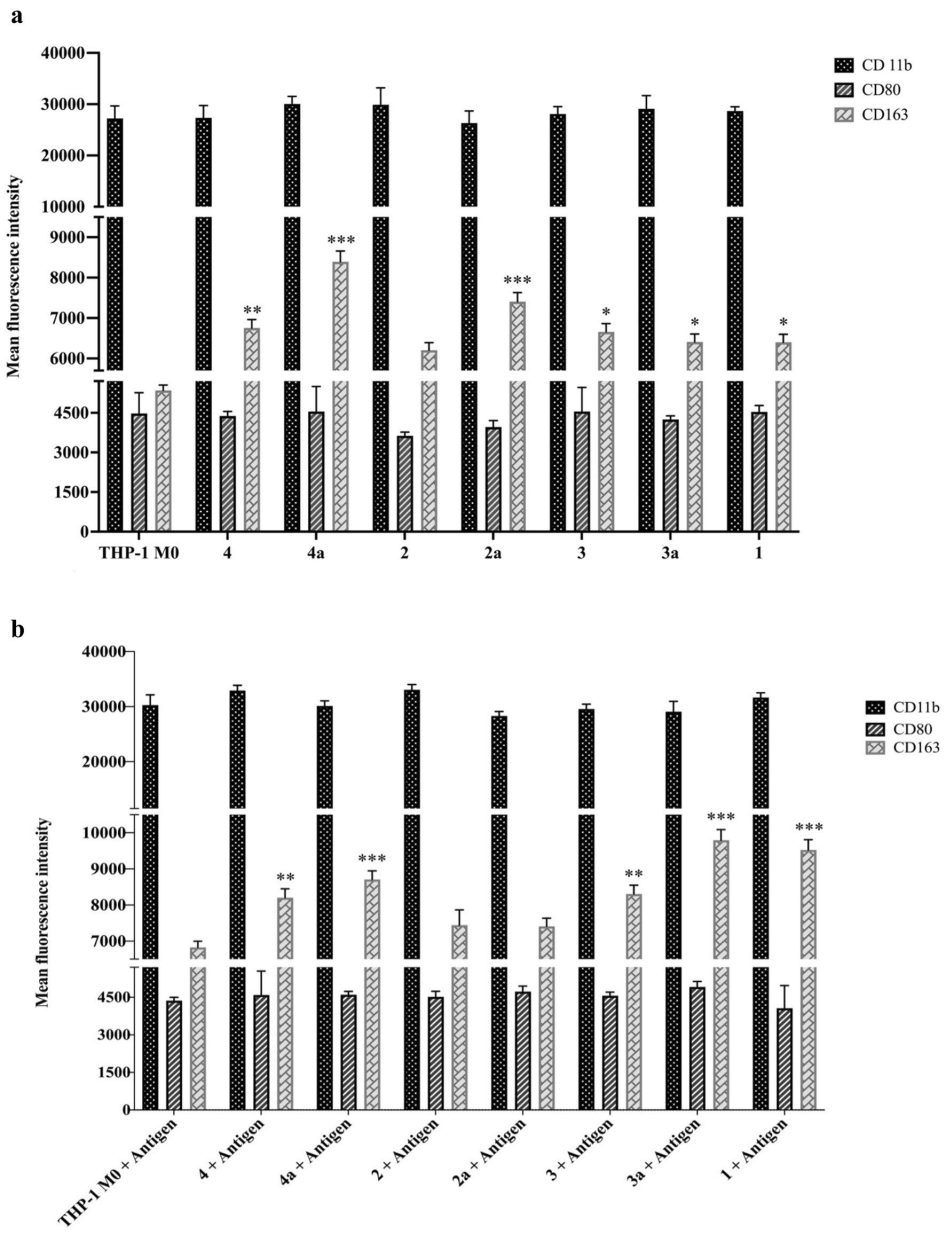
(**a**) Saponins without antigen treatment, (**b**) Saponins treated with antigen. THP-1 cells were treated with 15 ng/ml PMA and 3 µg/ml saponins and 1 HA titer of IBV D274 induced in the saponins with antigen group for 24 h incubation. The macrophage polarization was determined with flow cytometry. Each value represents the mean ± standard deviation of three independents measurements. (*P* < 0.05*, *P* < 0.01**, *P* < 0.001***).

The Mean Fluorescent Intensity (MFI) values (Table 5) of flow cytometry results showed that the MFI of CD11b in all THP-1 originated macrophage cells is almost near 30.000 which means all of the treated groups induced M0 polarization in THP-1 macrophages (Fig. 2). According to Fig. 2 in both antigen and without antigen saponin treated cells MFI values of CD163, the marker of M2 on macrophages when compared with CD80, the marker of M1, are higher which shows the saponins potential on M2 macrophage polarization. And also, it is worthwhile to mention the MFI values of cells treated with both antigen and saponin that is significantly higher than the cells without antigen (Fig. 2a,b). According to the fact that M1/M2 describes the two major and opposing activities of macrophages^33^. In the present study all of the samples showed the similar inverse relationship between CD80 and CD163’s MFI values. Based on our results, the saponins enhanced M2 polarization. In the present study, all samples showed a similar inverse relationship between the MFI values of CD80 and CD163. Compounds **4a**, **3a**, and **1** showed the most distinguished MFI values with antigen treatment with 8706 ± 239.19, 9794.8 ± 291.84, and 9522.65 ± 283.68 values (***P < 0.001), respectively, among the other compounds when treated with IBV D274 antigen (Table 4). Moreover, compounds **4a** and **2a** exhibited the highest CD163 MFI values without antigen treatment with MFI values of 8391.56 ± 264.75 and 7405.42 ± 224.13 (***P < 0.001), respectively (Table 5).

**Table 4:** MFI values of CD markers on THP-1 derived macrophage cells treated with saponins and antigen.

	CD 11b	CD80	CD163
THP-1 M0 + Ag	30,274.00 ± 1,859.74	4363.67 ± 133.29	6827.70 ± 169.38
**4** + Ag	32,925.30 ± 947.75	4593.32 ± 951.60	8203.00 ± 246.09**
**4a** + Ag	30,124.70 ± 913.74	4604.40 ± 128.49	8706.00 ± 239.19***
**2** + Ag	33,053.20 ± 951.60	4516.00 ± 223.23	7441.00 ± 423.23
**2a** + Ag	28,297.32 ± 808.92	4727.00 ± 221.96	7408.64 ± 224.16
**3** + Ag	29,545.10 ± 906.36	4564.51 ± 138.81	8303.34 ± 245.1**
**3a** + Ag	29,092.45 ± 852.78	4911.00 ± 224.16	9794.80 ± 291.84***
**1** + Ag	31,644.21 ± 849.30	4062.43 ± 906.36	9522.65 ± 283.68***

^a^**P < 0.01; ***P < 0.001.

^b^All values represented the mean ± standard deviation (n = 3 test).

**Table 5:** MFI values of CD markers on THP-1 derived macrophage cells treated with saponins.

	CD 11b	CD80	CD163
THP-1 M0	27,208.00 ± 2444.77	4,476.31 ± 786.24	5,346.00 ± 205.83
**4**	27,324.70 ± 2398.39	4,380.00 ± 169.38	6,755.64 ± 206.67**
**4a**	30,031.76 ± 1486.58	4,552.00 ± 947.76	8,391.56 ± 264.75**
**2**	29,875.39 ± 3311.75	3,629.71 ± 142.80	6,206.45 ± 185.19
**2a**	26,304.81 ± 2370.42	3,962.53 ± 246.09	7,405.42 ± 224.13***
**3**	28,092.69 ± 1435.47	4,549.73 ± 913.74	6,655.82 ± 206.67*
**3a**	29,092.75 ± 2580.19	4,248.00 ± 137.13	6,408.00 ± 195.24*
**1**	28,644.50 ± 849.33	4,536.00 ± 239.19	6,400.83 ± 196.02*

*P < 0.05; **P < 0.01; ***P < 0.001.

All values represented the mean ± standard deviation (n = 3test).

Regarding saponins, studies suggest that they can trigger Th2 in M0 macrophages, which in turn stimulates M2 macrophage polarization^34,35^. Furthermore, treating cells with both antigens and saponins can enhance macrophage polarization, leading to an improved immune system response. Macrophages, derived from monocytes, play a critical role in inflammation, host defense, and tissue healing. M2-polarized macrophages have potential as adjuvants for anticancer therapies, and recent approaches focus on M2 polarization. According to Zhao et al.^36^ demonstrated that *Panax* saponins promote M2 macrophage polarization so that depend on their effects of anti-inflammation, saponins have important role on treatment of vascular disease. Similarly, our findings demonstrate that saponins play an effective role in M2 macrophage polarization. As mentioned before, our findings indicate that saponins take an effective role in M2 macrophage polarization so that induce wound healing effect.

## Conclusion

This study evaluated the cytotoxic activities and macrophage polarization potential of four new oleanoic acid and hederagenin type triterpene saponins (**1–4**) named speciosides A-D and their new prosapogenins (**2a–4a**), respectively. The 4 new saponins and 3 prosapogenins were tested for their cytotoxicity. Consequently, **1**, **2a**, and **4a** exhibited significant cytotoxicity against A549 cells and MDA-MB-231 cells. All isolated new saponins (**1–4**) and their prosapogenins (**2a–4a**) induced M2 macrophage polarization. These results provide valuable information for the literature on the structure, cytotoxicity, and immunomodulatory relationship of the studied saponins. Further studies should explore the activity-structure relationships to establish a model for activity-targeted synthesis and semi-synthesis. Moreover, additional research on the biological activities of these saponins for pharmaceutical and industrial applications is recommended.

### Supplementary Information

Supplementary Information.

## Data Availability

Data generated or analyzed during the study are included in the Supplementary Information. The data used in this current study are available from the corresponding authors at a reasonable request.
