# Supplementary material for: Four new triterpene saponins from Cephalaria speciosa and their potent cytotoxic and immunomodulatory activities
Source: Sci Rep. 2023 Oct 8;13:16964. doi: 10.1038/s41598-023-44114-6 (PMC10560666; doi:10.1038/s41598-023-44114-6)
Supplement: Supplementary file 1 — Supplementary Information. [file 41598_2023_44114_MOESM1_ESM.docx]

**Four new triterpene saponins from *Cephalaria speciosa* and their potent cytotoxic and immunomodulatory activities**

Ozan Oztunc^1^, Gaye Sumer Okkali^1^, Sevda Zeinali^2^, Ayse Nalbantsoy^3^ and Nazli Boke Sarikahya^1*^

^1^Department of Chemistry, Faculty of Science, Ege University, 35100, Bornova, Izmir, Turkiye

^2^Department of Biotechnology, Faculty of Science, Ege University, 35100, Bornova, Izmir, Turkiye

^3^Department of Bioengineering, Faculty of Engineering, Ege University, 35100, Bornova, Izmir, Turkiye

**Table of contents**

Figure ***S* 1**. Structure of compound **1**........................................................................................4

Figure **S 2**. HR-ESI/MS spectrum of compound **1**.....................................................................5

Figure ***S* 3**. FTIR spectrum of compound **1**................................................................................6

Figure ***S* 4**. 1H-NMR spectrum of compound **1** (600 MHz, DMSO-*d*6).....................................7

Figure ***S* 5**. 13C-NMR spectrum of compound **1** (150 MHz, DMSO-*d*6) ...................................8

Figure ***S* 6**. COSY spectrum of compound **1**..............................................................................9

Figure ***S* 7**. HSQC spectrum of compound **1**............................................................................11

Figure ***S* 8**. HMBC spectrum of compound **1**............................................................................12

Figure ***S* 9**. Structure of compound **2**.......................................................................................13

Figure ***S* 10**. HR-ESI/MS spectrum of compound **2**..................................................................14

Figure ***S* 11**. FTIR spectrum of compound **2**.............................................................................15

Figure ***S* 12**. 1H-NMR spectrum of compound **2** (400 MHz, DMSO-*d*6)...................................16

Figure ***S* 13**. 13C-NMR spectrum of compound **2** (100 MHz, DMSO-*d*6)..................................17

Figure ***S* 14**. COSY spectrum of compound **2**............................................................................18

Figure ***S* 15**. HSQC spectrum of compound **2**...........................................................................19

Figure ***S* 16**. HMBC spectrum of compound **2**..........................................................................20

Figure ***S* 17**. Structure of compound **3**.....................................................................................21

Figure ***S* 18**. HR-ESI/MS spectrum of compound **3**..................................................................22

Figure ***S* 19**. FTIR spectrum of compound **3**.............................................................................23

Figure ***S* 20**. 1H NMR spectrum of compound **3** (600 MHz, DMSO-*d*6)...................................24

Figure ***S* 21**. 13C-NMR spectrum of compound **3** (150 MHz, DMSO-*d*6)..................................25

Figure ***S* 22**. COSY spectrum of compound **3**............................................................................26

Figure ***S* 23**. HSQC spectrum of compound **3**...........................................................................27

Figure ***S* 24**. HMBC spectrum of compound **3**..........................................................................28

Figure ***S* 25**. Structure of compound **4**.....................................................................................29

Figure ***S* 26**. HR-ESI/MS spectrum of compound **4**..................................................................30

Figure ***S* 27**. FTIR spectrum of compound **4**.............................................................................31

Figure ***S* 28**. 1H NMR spectrum of compound **4** (400 MHz, DMSO-*d*6)....................................32

Figure ***S* 29**. 13C NMR spectrum of compound **4** (100 MHz, DMSO-*d*6)..................................33

Figure ***S* 30**. COSY spectrum of compound **4**............................................................................34

Figure ***S* 31**. HSQC spectrum of compound **4**...........................................................................35

Figure ***S* 32**. HMBC spectrum of compound **4**..........................................................................36

Figure ***S* 33**. HMBC spectrum of compound **4**..........................................................................37

Figure ***S* 34**. Comparison between A-1H-NMR of compound **2** and B- compound **2a**.............38

Figure ***S* 35**. Comparison between A-1H-NMR of compound **3** and B- compound **3a**.............39

Figure ***S* 36**. Comparison between A-1H-NMR of compound **4** and B- compound **4a**.............40

Figure ***S* 37**. GC-MS spectrum of silylated standard sugars.....................................................41

Figure ***S* 38**. GC-MS spectrum of silylated monosaccharides from compound **1**.....................42

Figure ***S* 39**. GC-MS spectrum of silylated monosaccharides from compound **2**.....................43

Figure ***S* 40**. GC-MS spectrum of silylated monosaccharides from compound **3**.....................44

Figure ***S*** **41**. GC-MS spectrum of silylated monosaccharides from compound **4**.....................45

Figure **S 42**. Structure of compound **5**…………………………………………………………………………………46

Figure **S 43**. ^1^H NMR spectrum of compound **5** ……………………………………………………………..……47

Figure **S 44**. ^13^C NMR spectrum of compound **5**…………………………………………………………………..48

Figure **S 45**. Structure of compound **6** ………………………………………………………………………………..49

Figure **S 46**. ^1^H NMR spectrum of compound **6**………………………………………………………….……..…50

Figure **S 47**. ^13^C NMR spectrum of compound **6**…………...........................................................51

Figure **S 48**. Structure of compound **7**…………………………………………………………..………….………..52

Figure **S 49**. ^1^H NMR spectrum of compound **7**……………………………………………….……………..…..53

Figure **S 50**. ^13^C NMR spectrum of compound **7**………………………………………..………………………..54

Figure **S 51**. Structure of compound **8** ……………………………………………………………………….……….55

Figure **S 52**. ^1^H NMR spectrum of compound **8**……………………………………………….……………..…..56

Figure **S 53**. ^13^C NMR spectrum of compound **8**………………………………………..………………………..57

Figure **S 54**. Structure of compound **9** ……………………………………………………………………….……….58

Figure **S 55**. ^1^H NMR spectrum of compound **9**……………………………………………….……………..…..59

Figure **S 56**. ^13^C NMR spectrum of compound **9**………………………………………..………………………..60

Figure **S 57**. Structure of compound **10** …………………………………………………………………….……….61

Figure **S 58**. ^1^H NMR spectrum of compound **10**……………………………………………..……………..…..62

Figure **S 59**. ^13^C NMR spectrum of compound **10**………………………………………..……….……………..63

Figure ***S*** **60**. A549 Cell viability following 48 h treatment of the compound usage................64

Figure ***S*** **61**. CCD34-Lu Cell viability following 48 h treatment of the compound usage..........64

Figure ***S*** **62**. MDA-MB-231 Cell viability following 48 h treatment of the compound usage...65

Figure ***S* 63**. PC-3 Cell viability following 48 h treatment of the compound usage..................65

Figure ***S* 64**. U-87 MG Cell viability following 48 h treatment of the compound usage...........66

Figure ***S* 65**. U-87 MG Cell viability following 48 h treatment of the compound usage...........66

Figure ***S* 66**. HeLa Cell viability following 48 h treatment of the compound usage.................67

Figure ***S* 67**. HepG-2 Cell viability following 48 h treatment of the compound usage.............67

Figure **S *1***. Structure of compound **1**


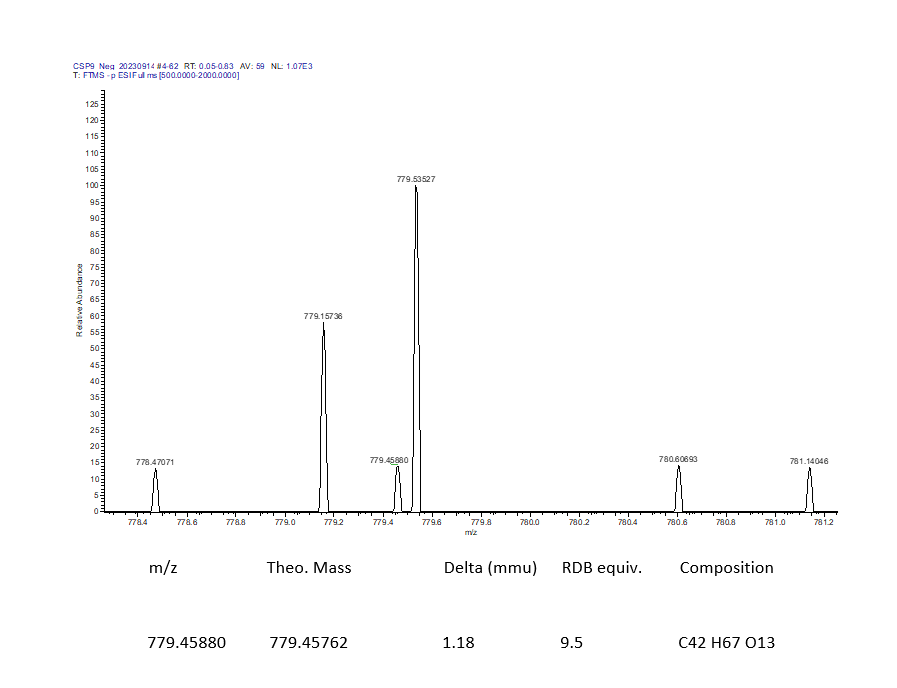


Figure **S *2****.* HR-ESI/MS spectrum of compound **1**


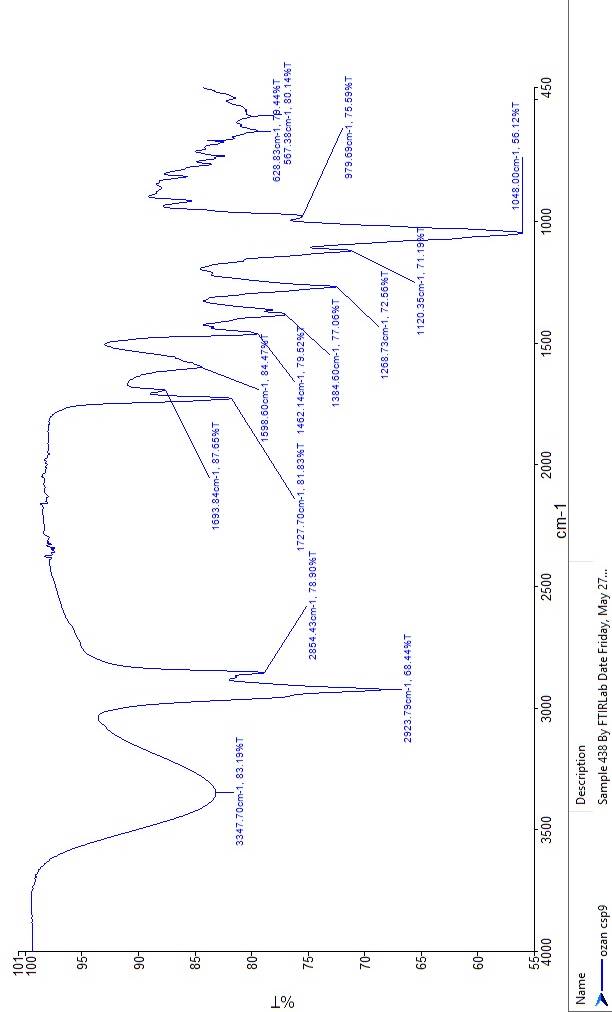


Figure **S *3***. FTIR spectrum of compound **1**


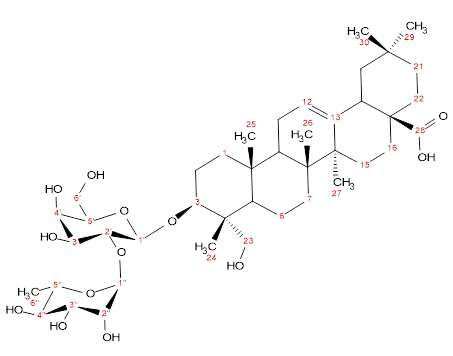

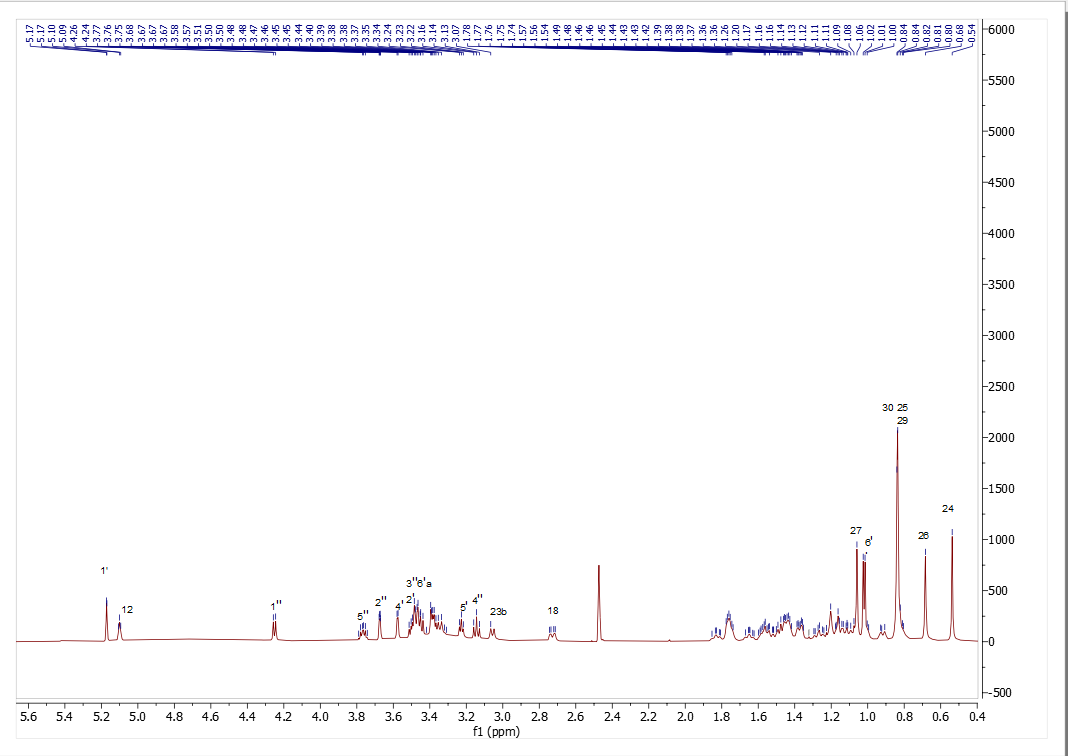
 Figure **S *4****.* ^1^H-NMR spectrum of compound **1** (600 MHz, DMSO-*d_6_*)


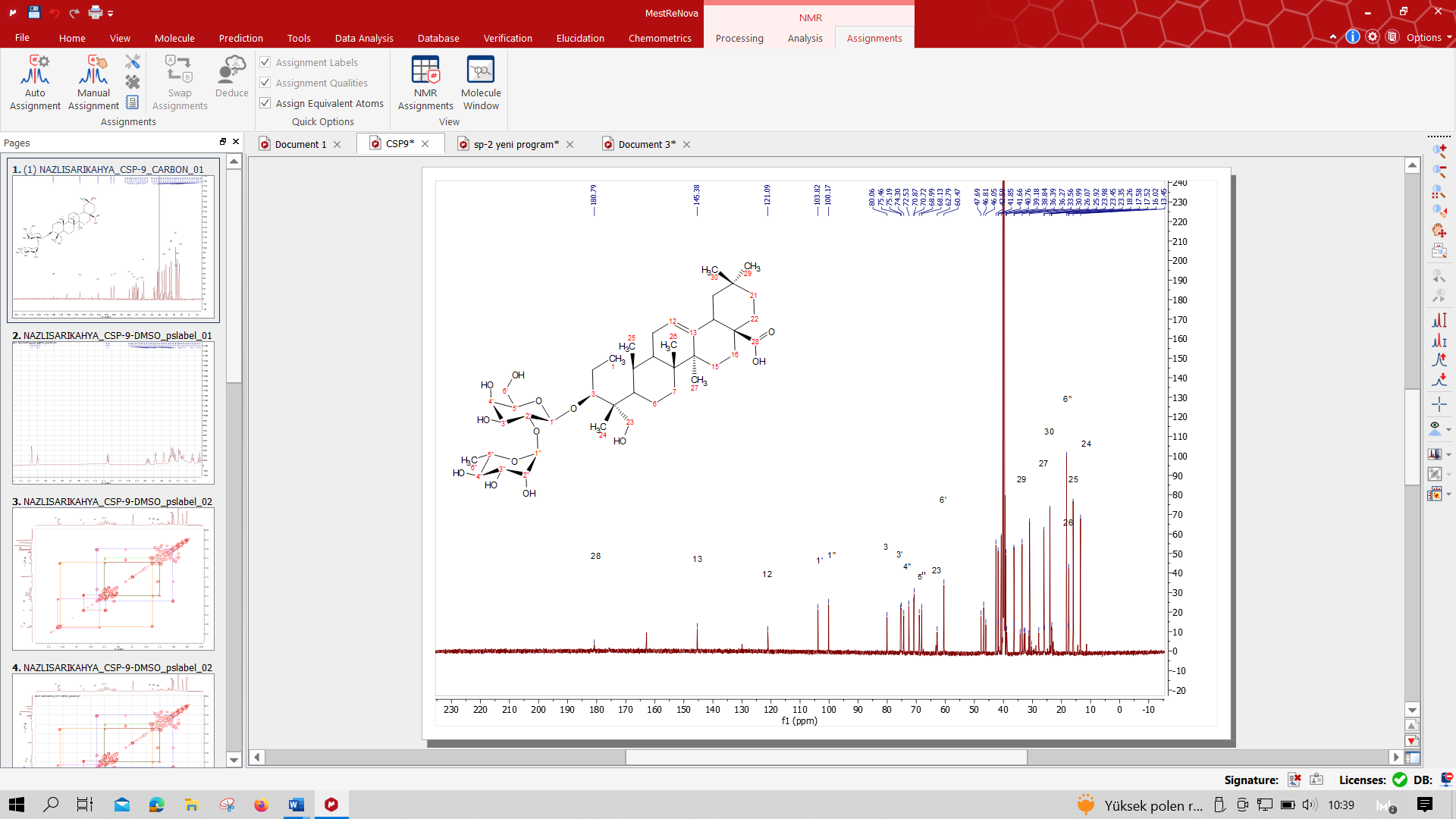


Figure **S *5***. ^13^C-NMR spectrum of compound **1** (150 MHz, DMSO-*d_6_*)


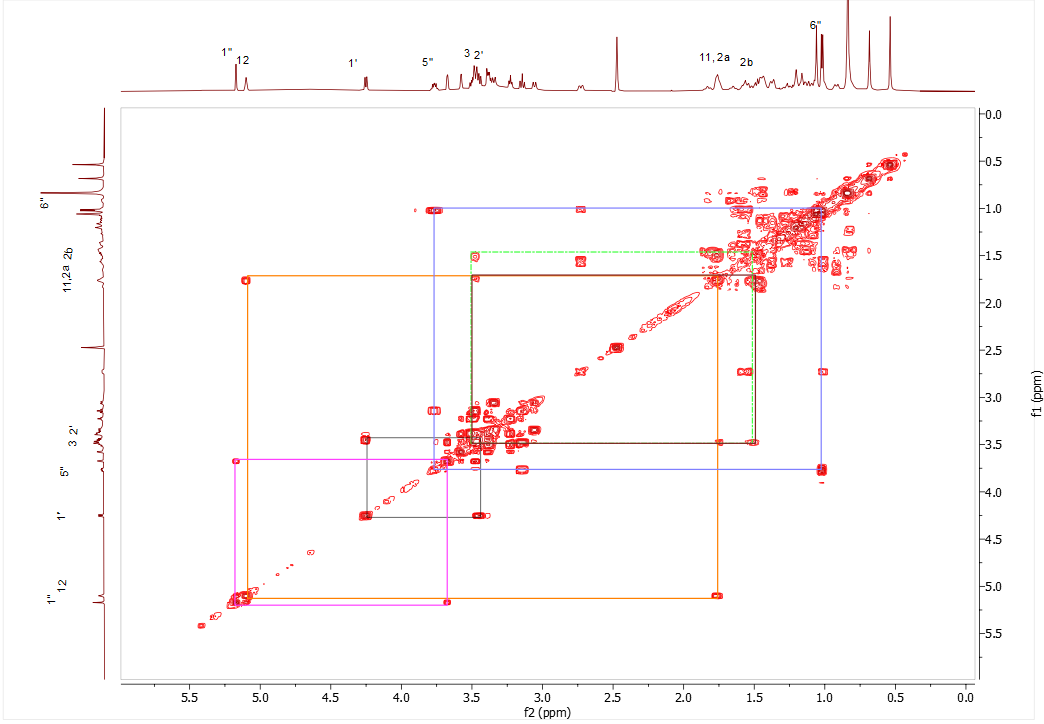


Figure **S *6***. COSY spectrum of compound **1**


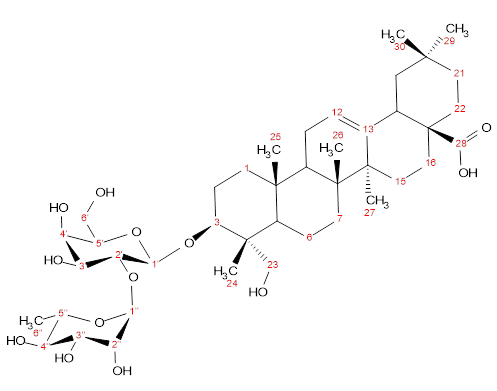

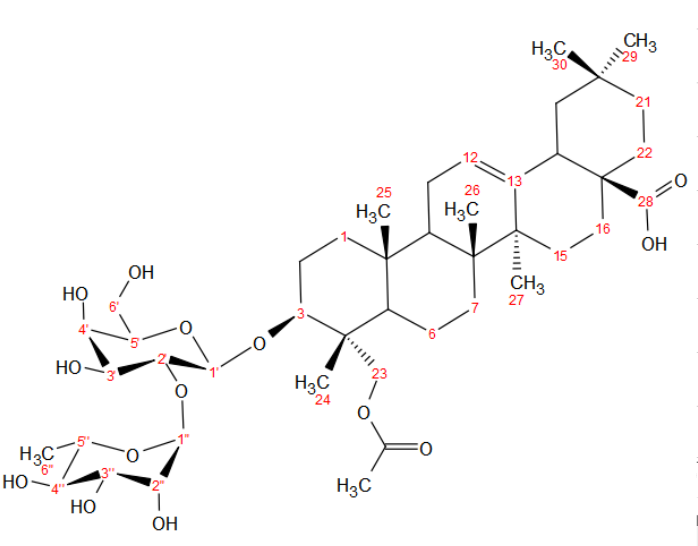

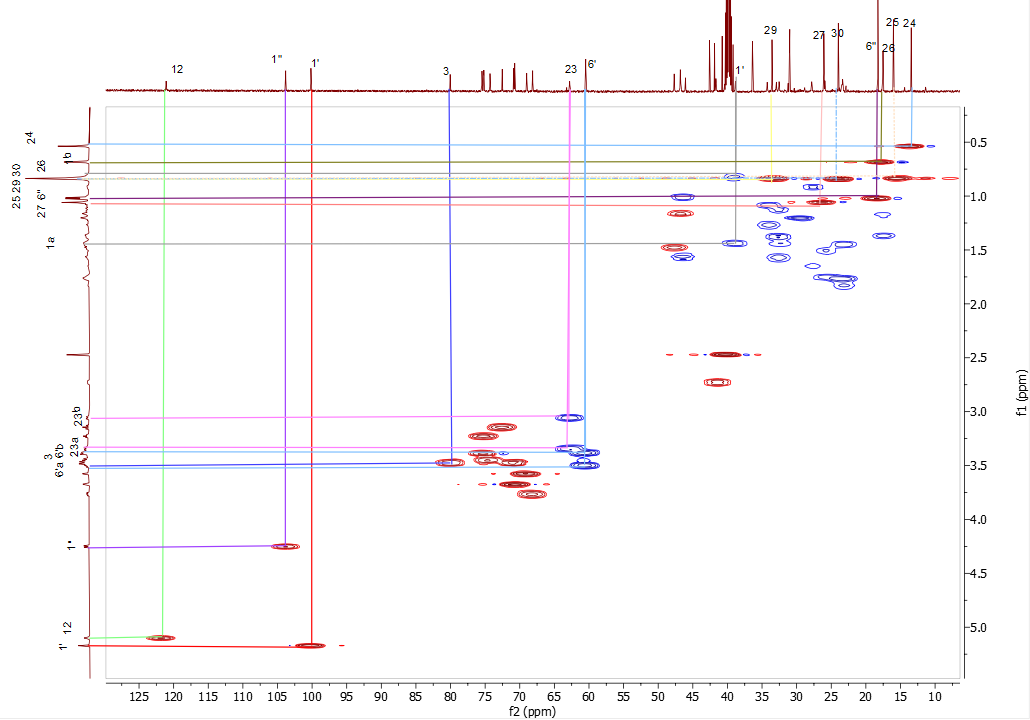


Figure **S *7***. HSQC spectrum of compound **1**


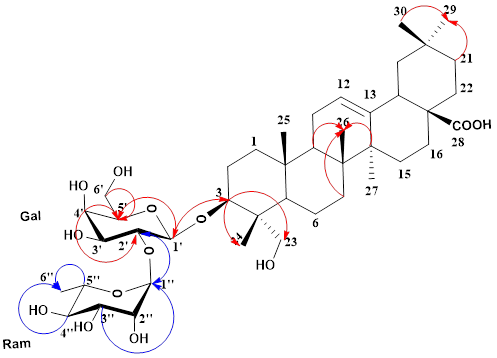

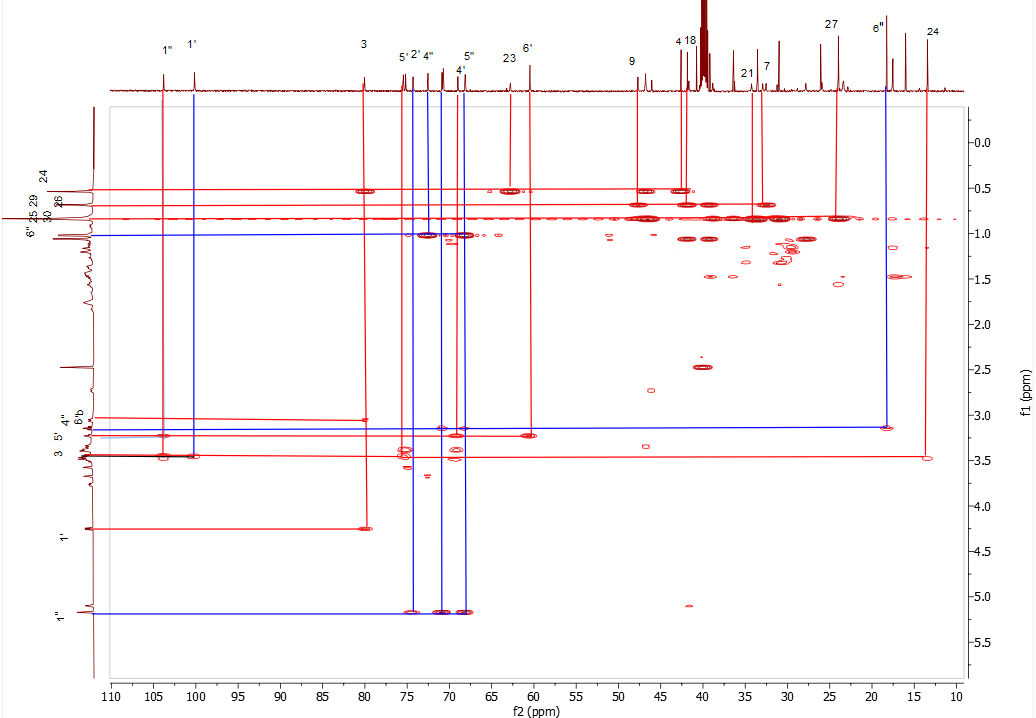
 Figure **S *8***. HMBC spectrum of compound **1**

Figure **S *9***. Structure of compound **2**


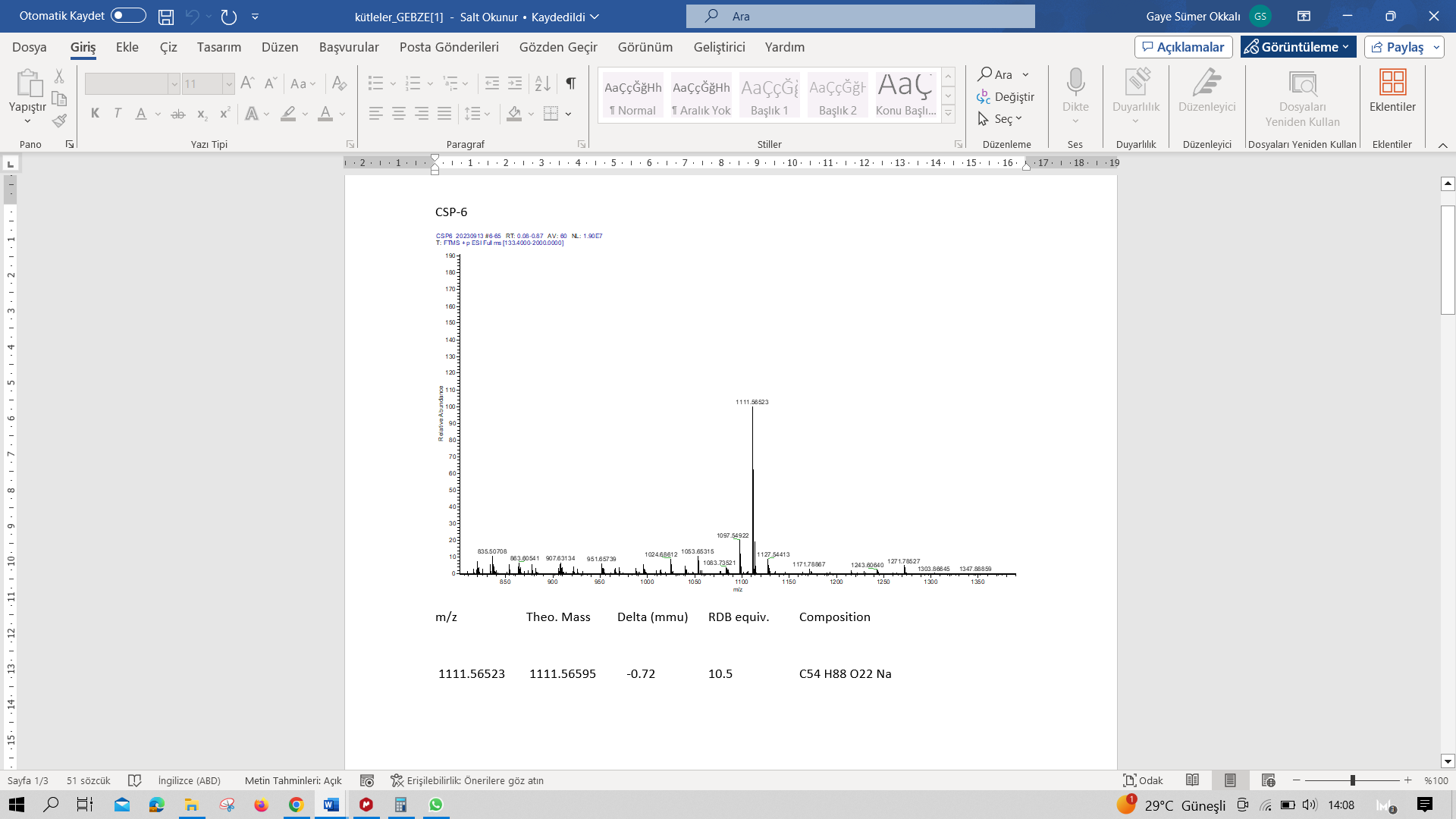


Figure ***S 10***. HR-ESI/MS spectrum of compound **2**

**
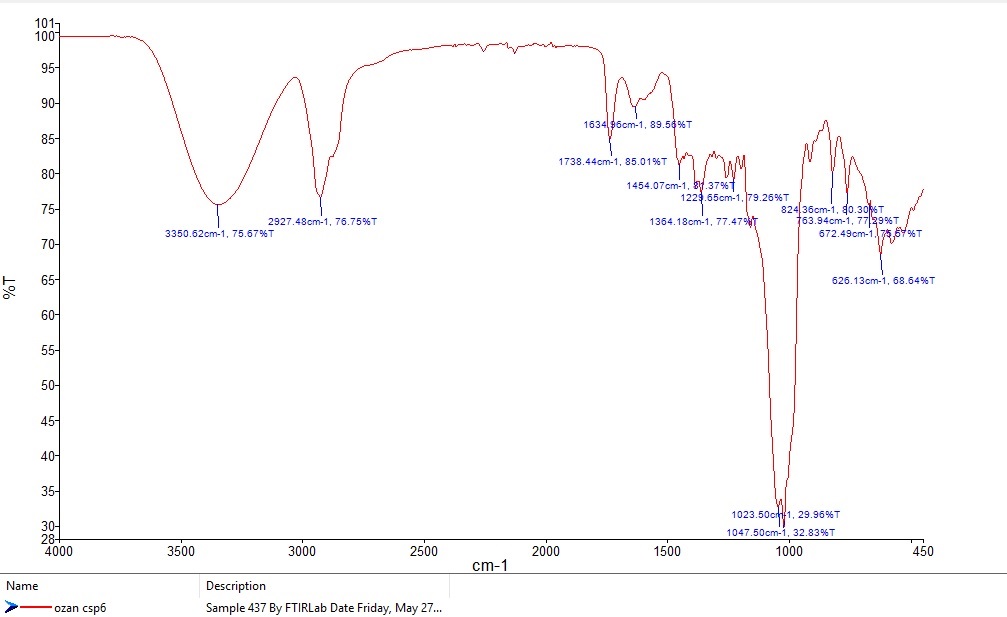
**Figure **S *11***. FTIR spectrum of compound **2
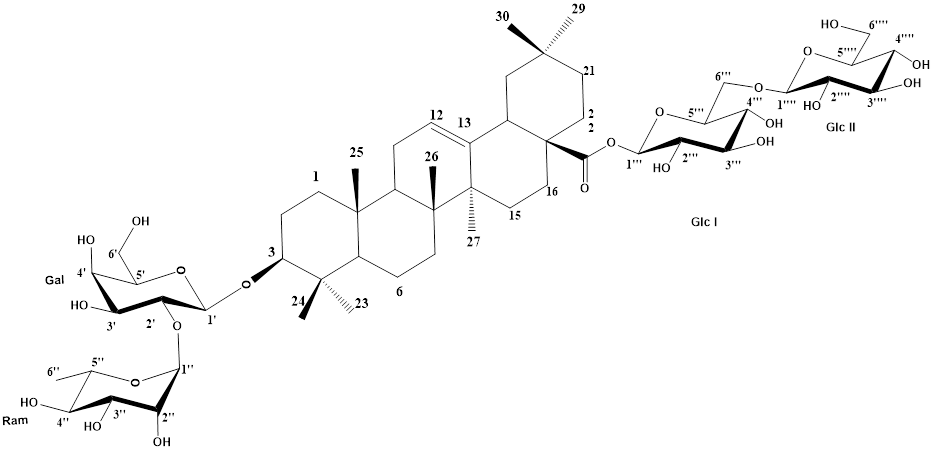
*
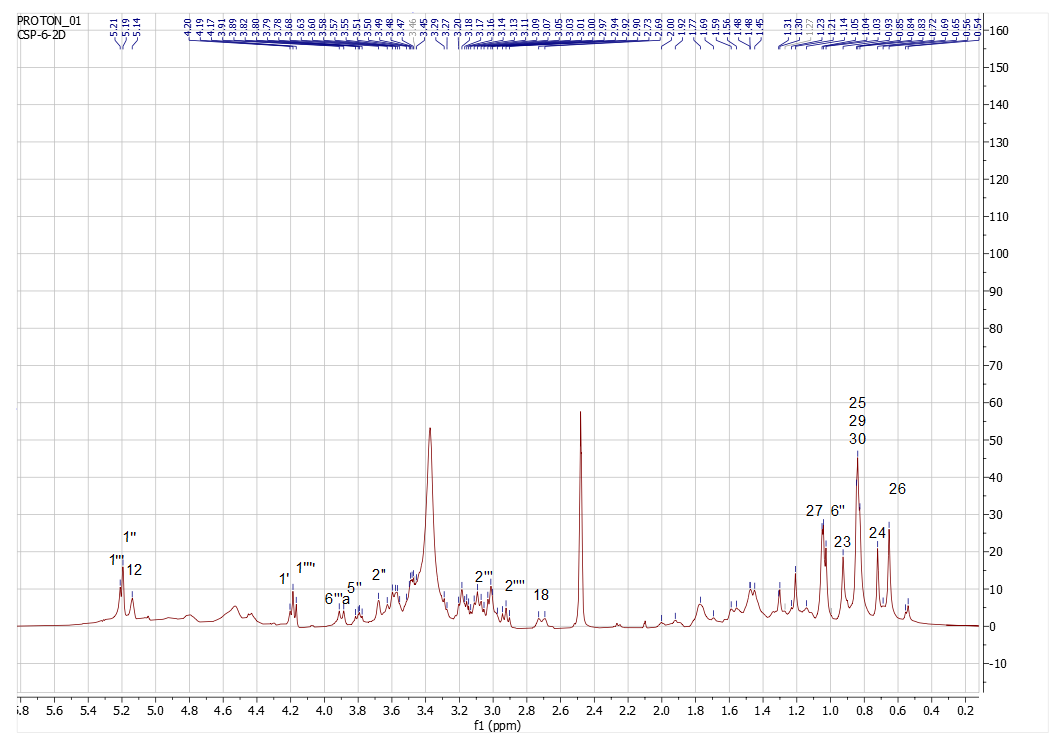
***

Figure **S *12***. ^1^H-NMR spectrum of compound **2** (400 MHz, DMSO-*d_6_*)

**
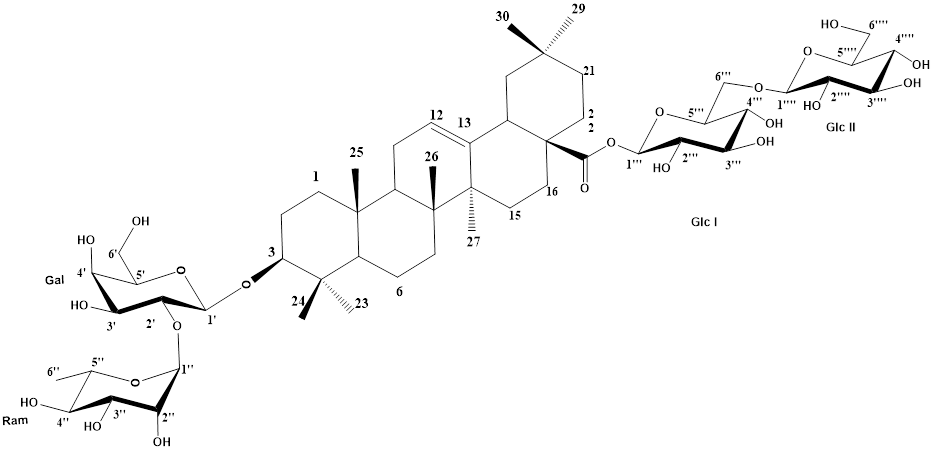
***
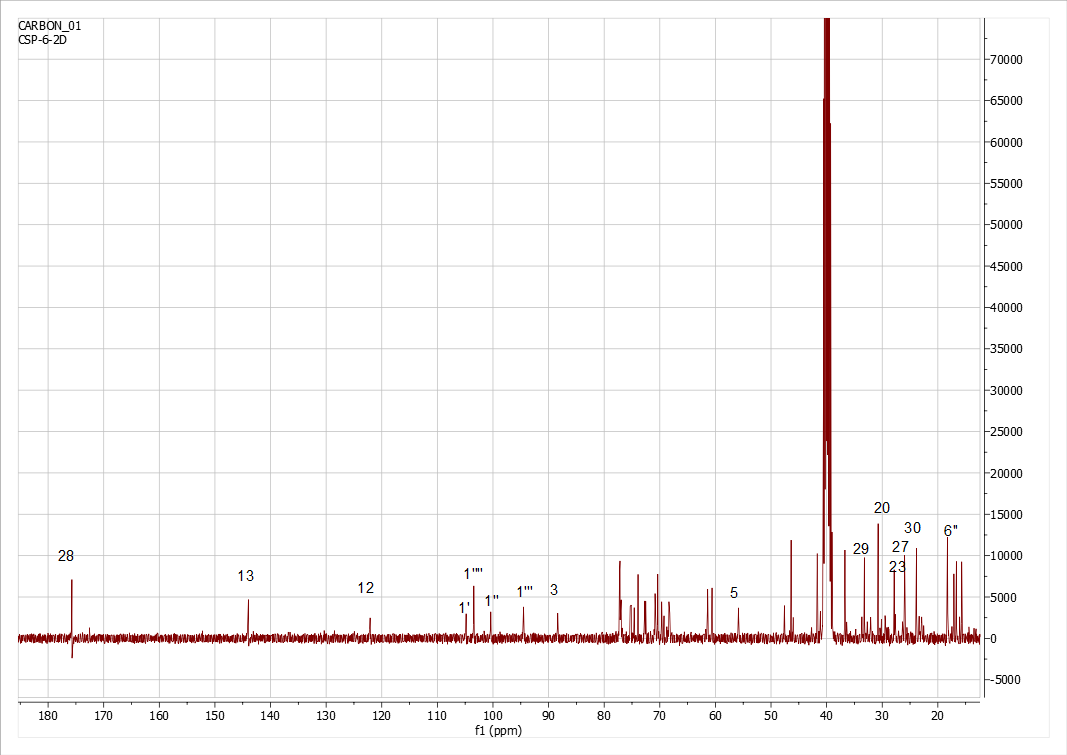
*

Figure **S *13***. ^13^C-NMR spectrum of compound **2** (100 MHz, DMSO-*d_6_*)


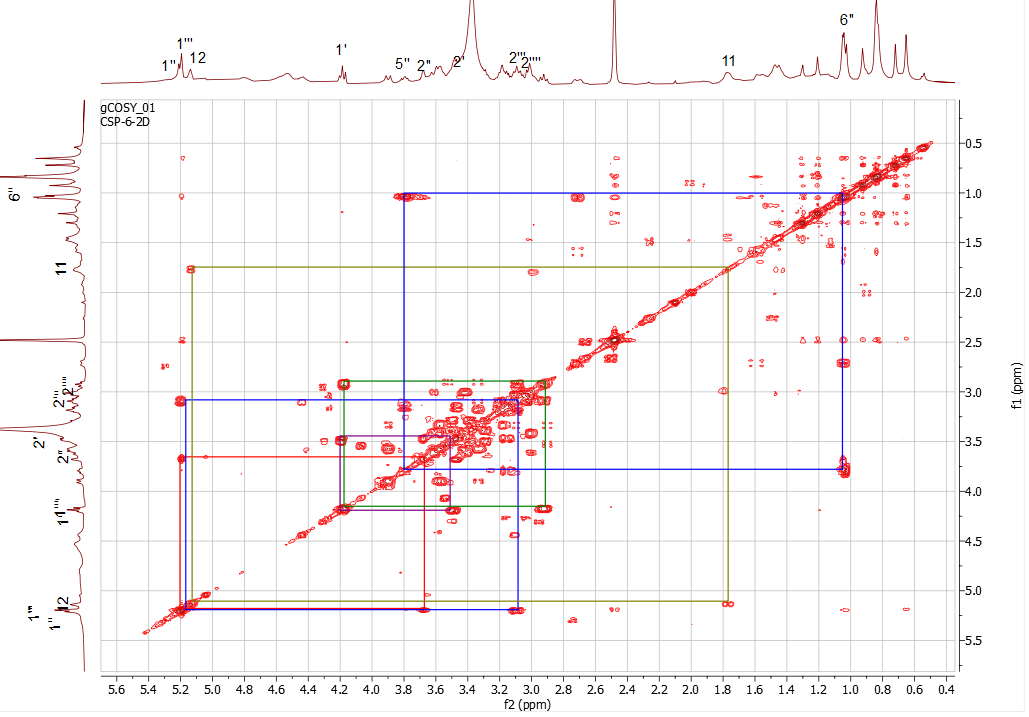
 Figure ***S 14***. COSY spectrum of compound **2**


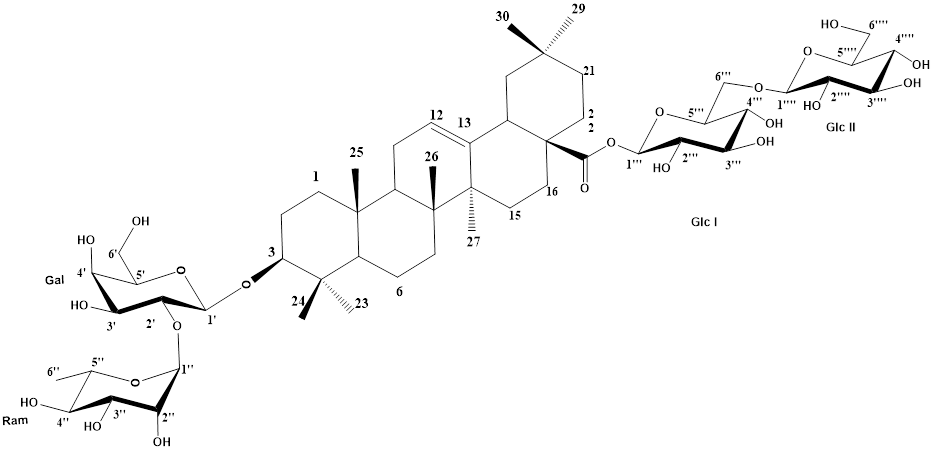


Figure **S *15***. HSQC spectrum of compound **2**


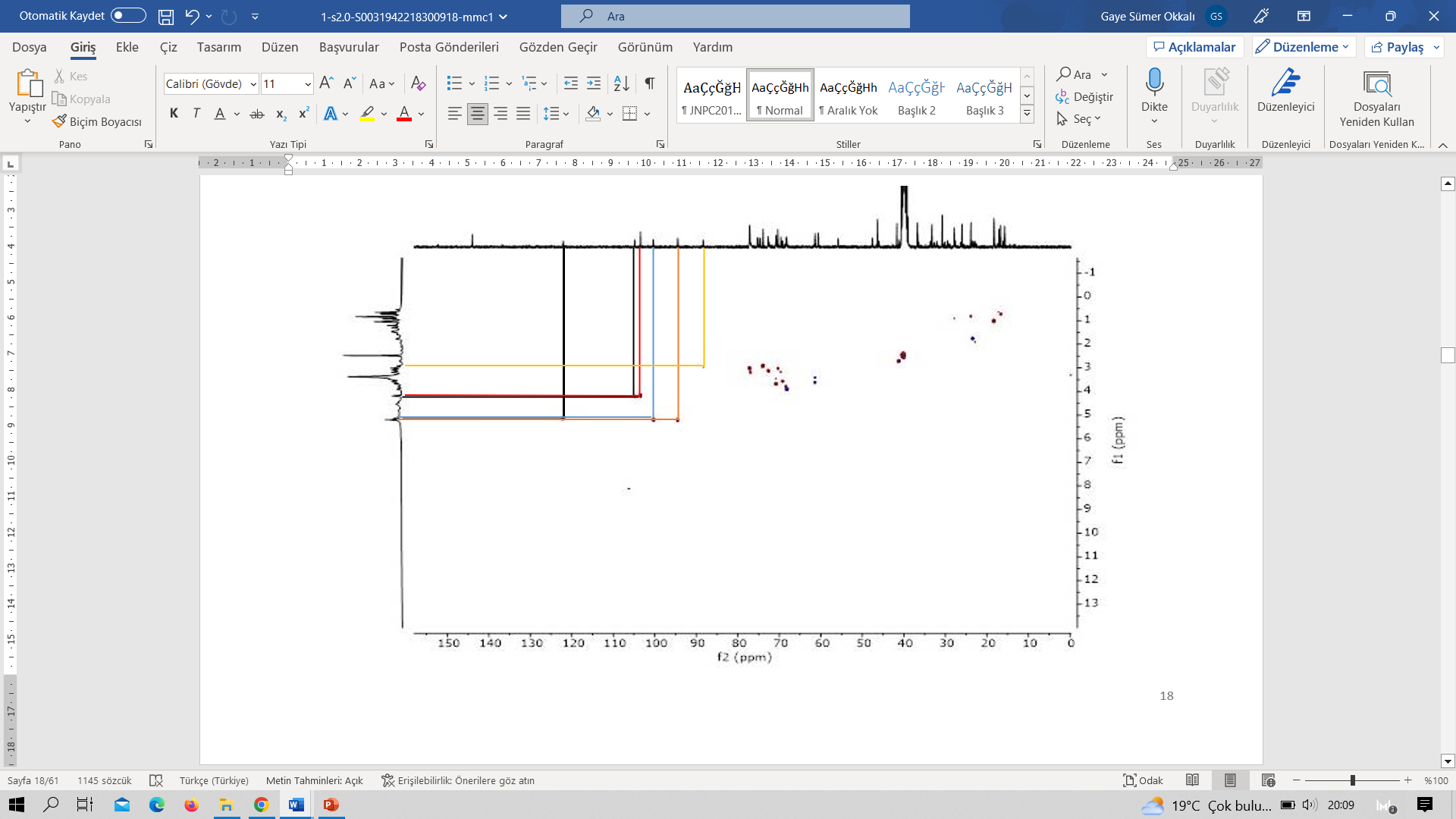


13

12

1’

1’’’’

1’’

1’’’

3

12

1’’

1’’’

3

1’

1’’’’


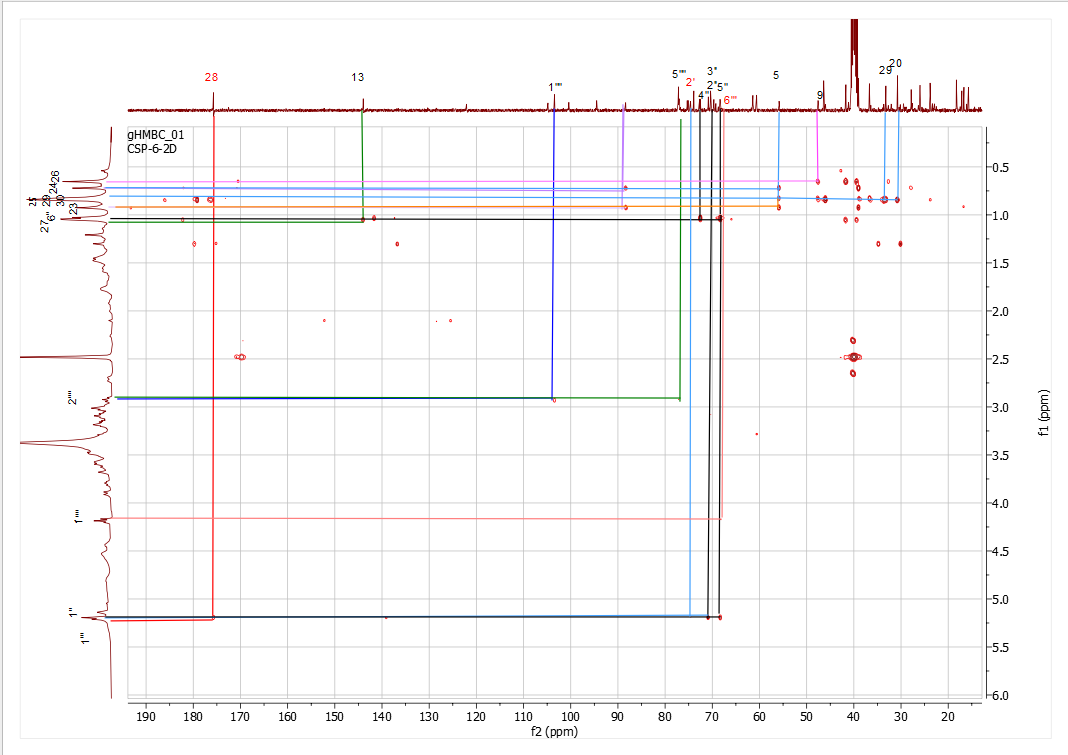


Figure **S *16***. HMBC spectrum of compound **2**

Figure **S *17****.* Structure of compound **3**


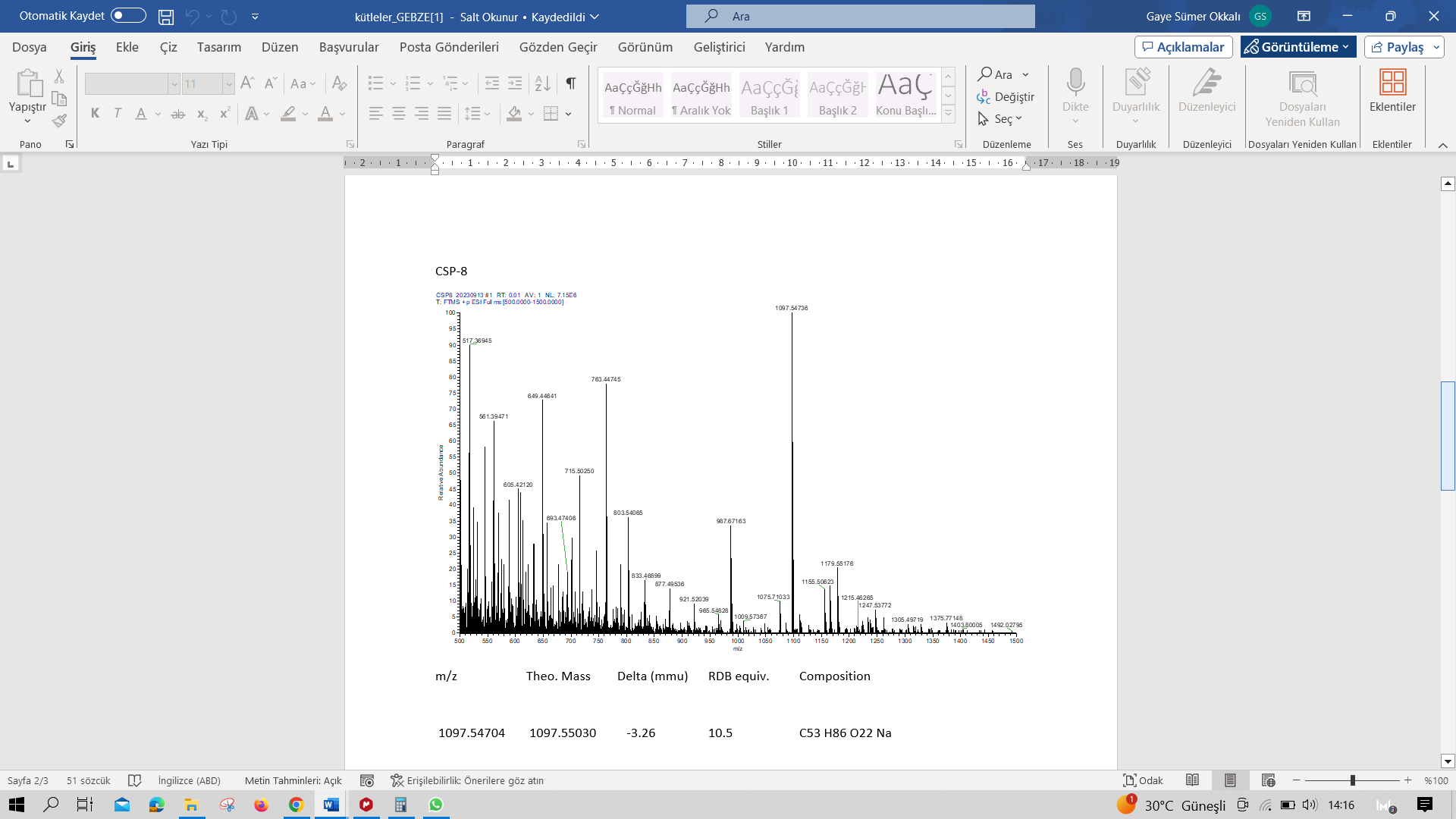


Figure **S *18***. HR-ESI/MS spectrum of compound **3**

**
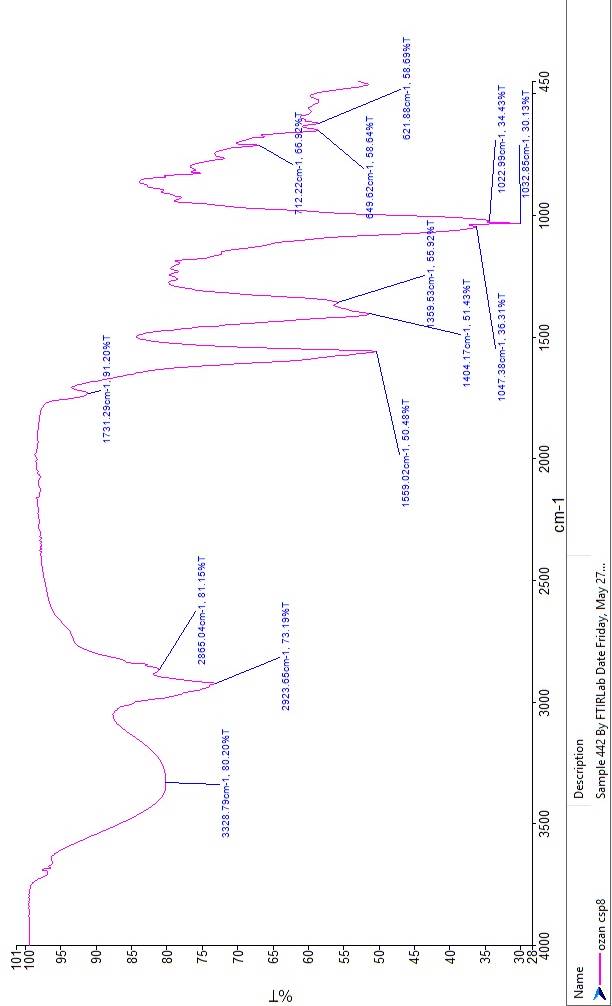
**

Figure **S *19***. FTIR spectrum of compound **3**


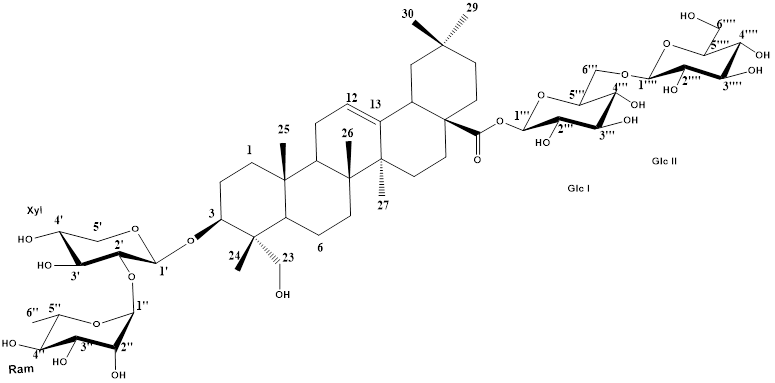

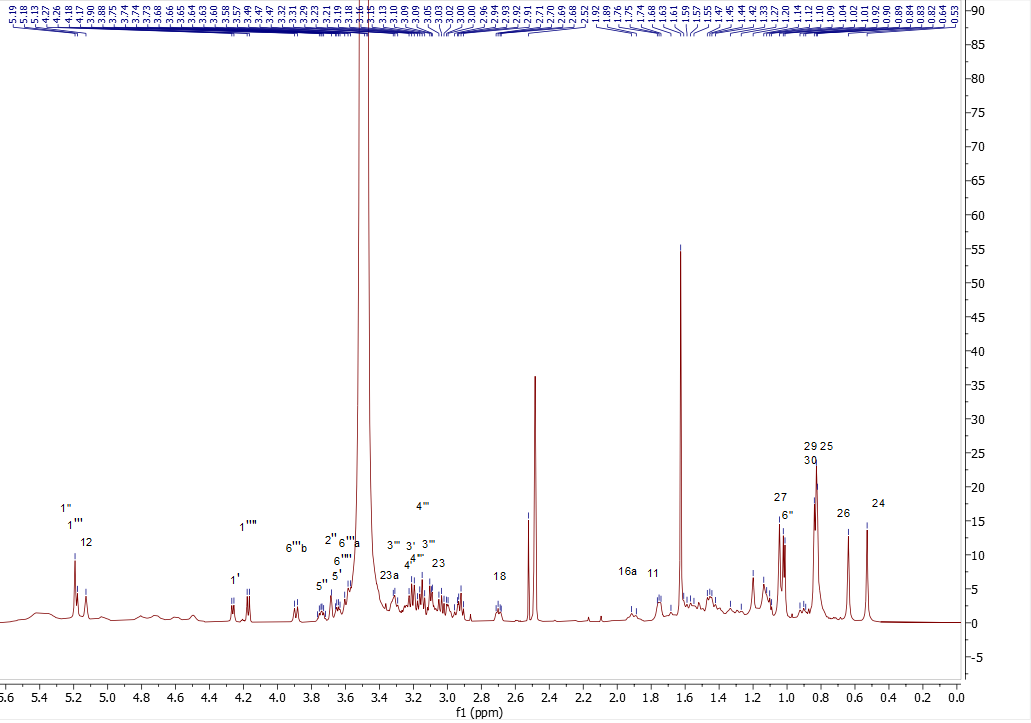


Figure **S *20***. ^1^H NMR spectrum of compound **3** (600 MHz, DMSO-*d_6_*)


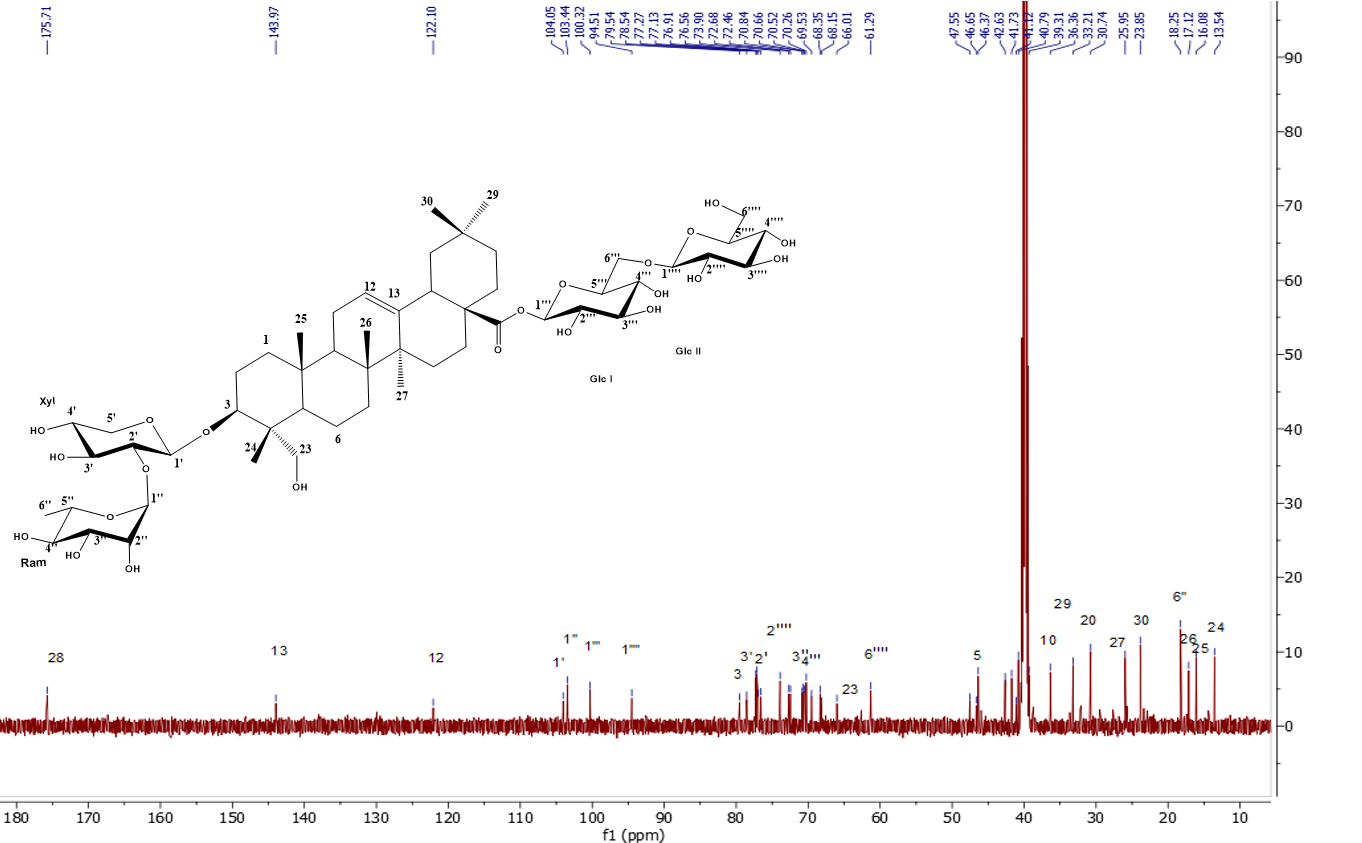


Figure **S *21***. ^13^C-NMR spectrum of compound **3** (150 MHz, DMSO-*d_6_*)


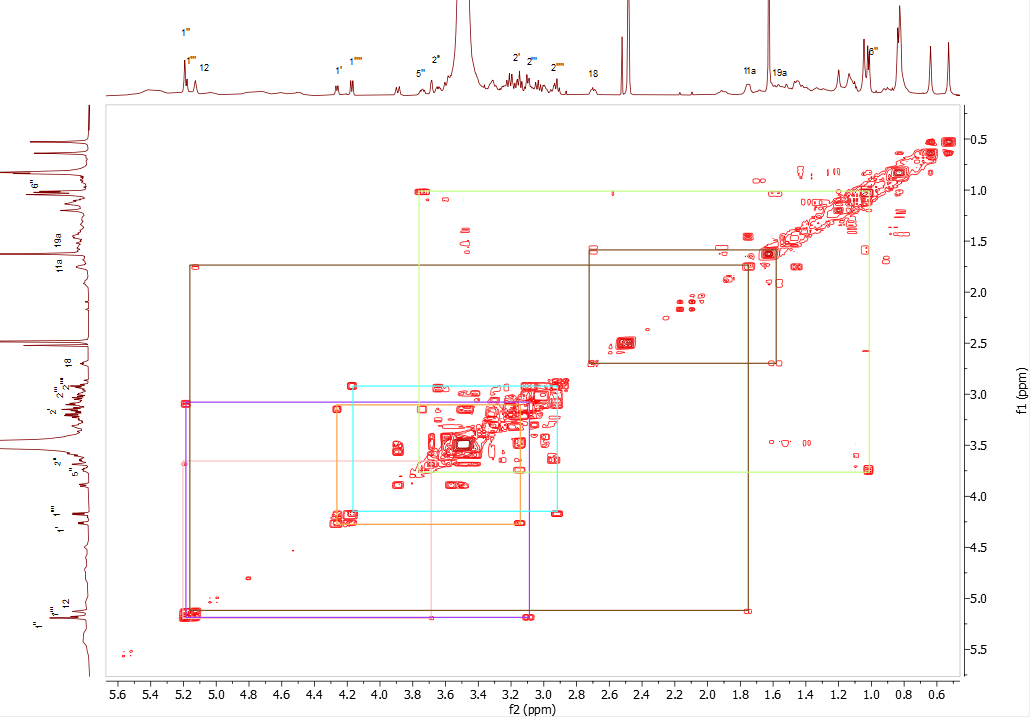

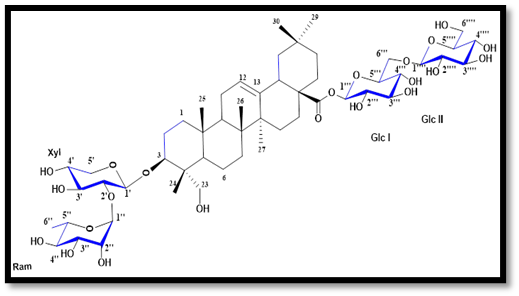


Figure **S *22***. COSY spectrum of compound **3**


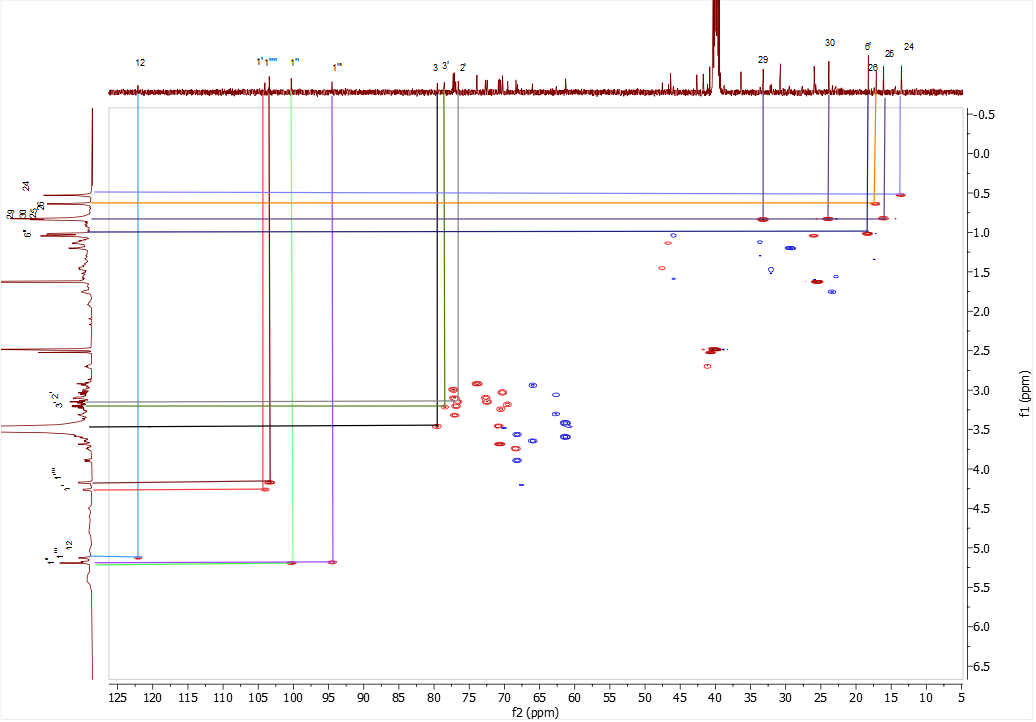


Figure **S *23***. HSQC spectrum of compound **3**


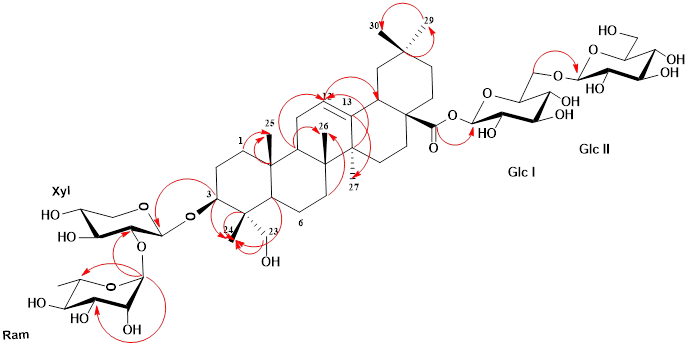

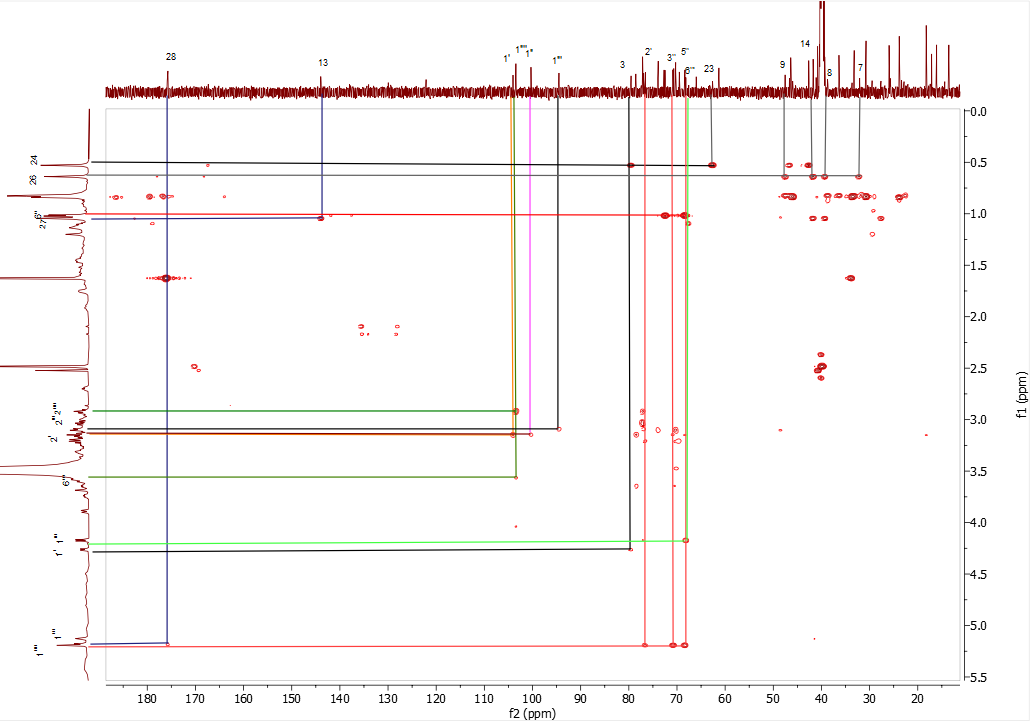


Figure **S *24***. HMBC spectrum of compound **3**

Figure **S *25***. Structure of compound **4**


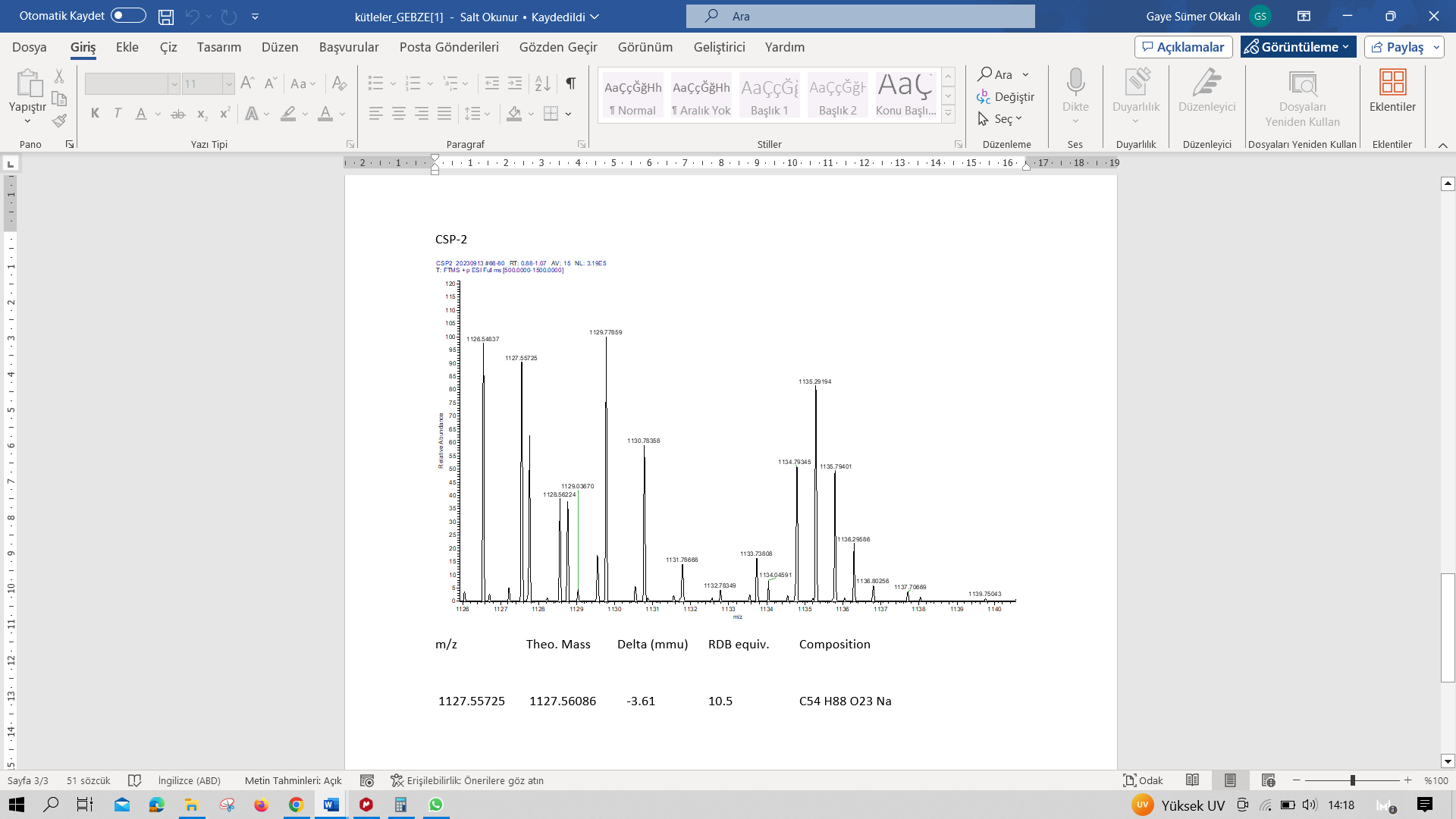


Figure **S *26***. HR-ESI/MS spectrum of compound **4**


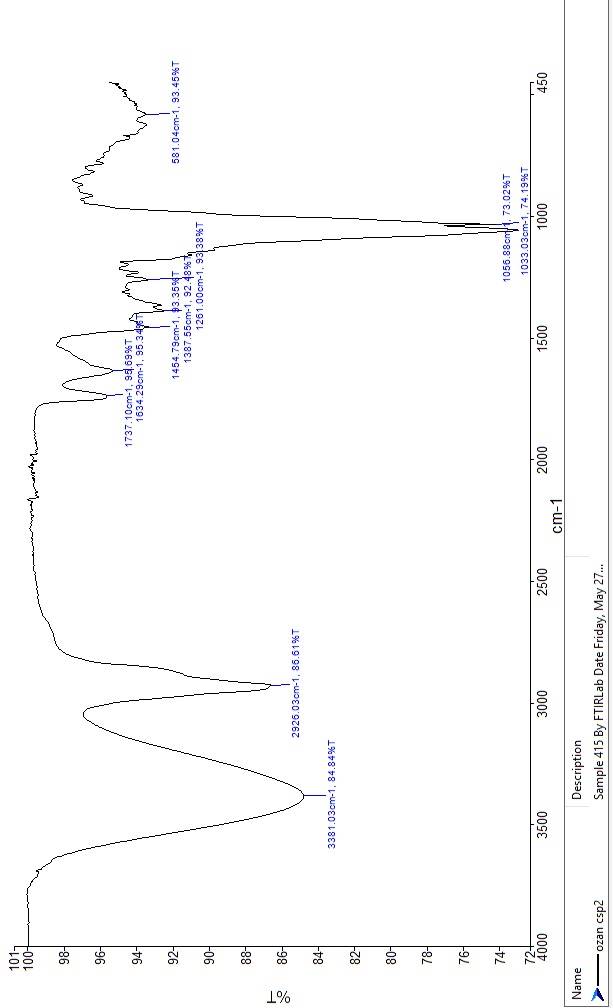


Figure **S *27***. FTIR spectrum of compound **4**


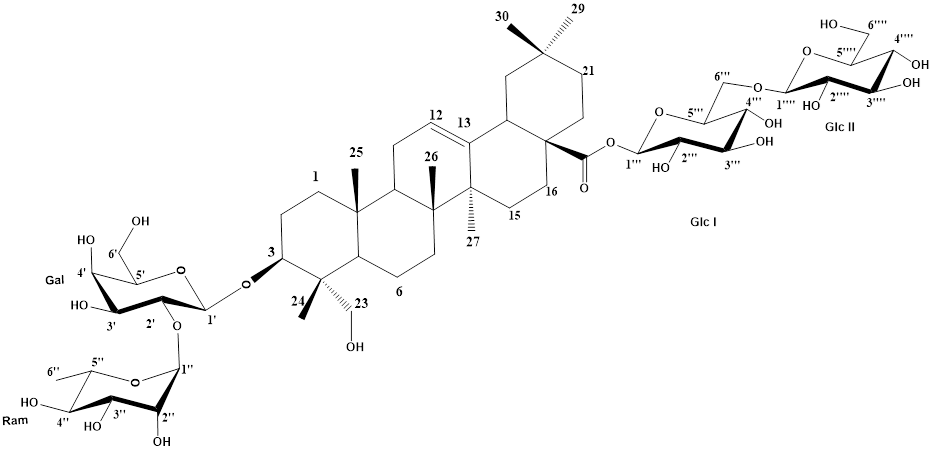

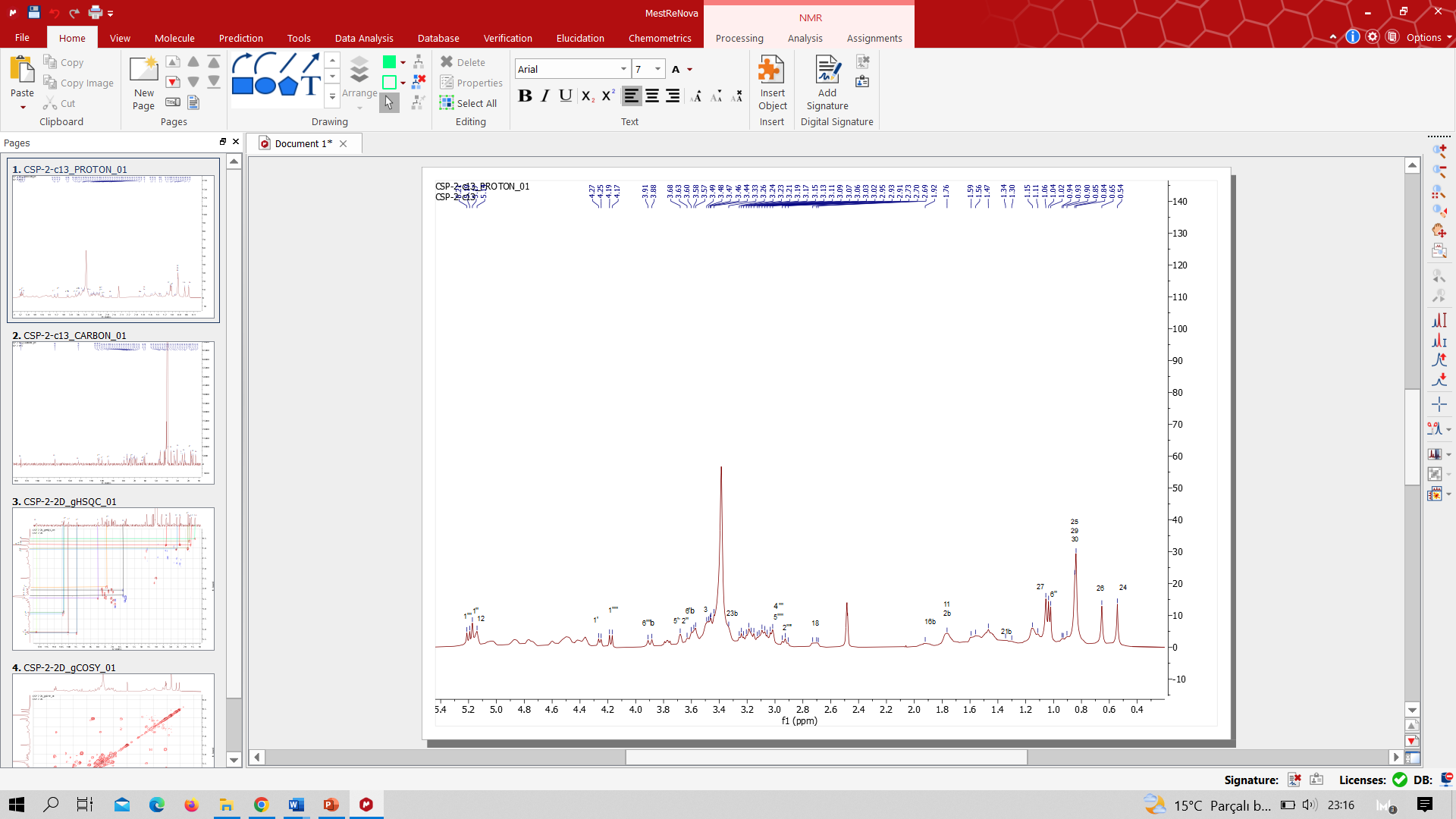


Figure **S *28***. ^1^H NMR spectrum of compound **4** (400 MHz, DMSO-*d_6_*)


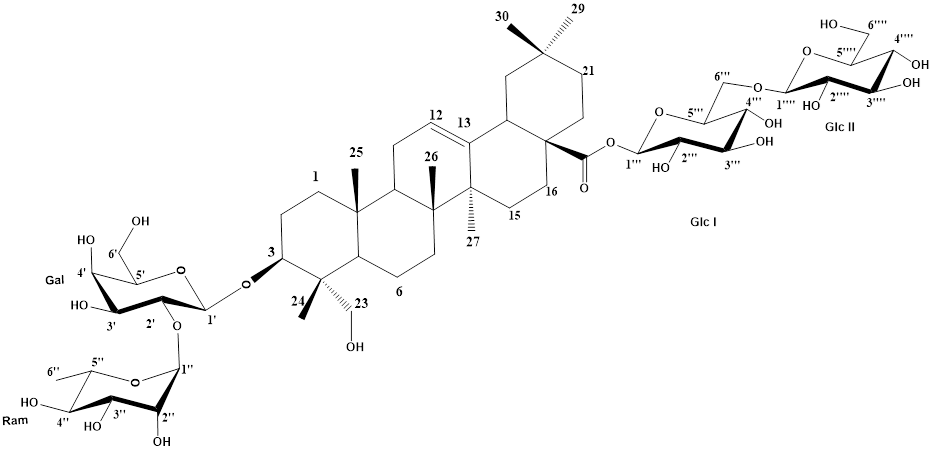

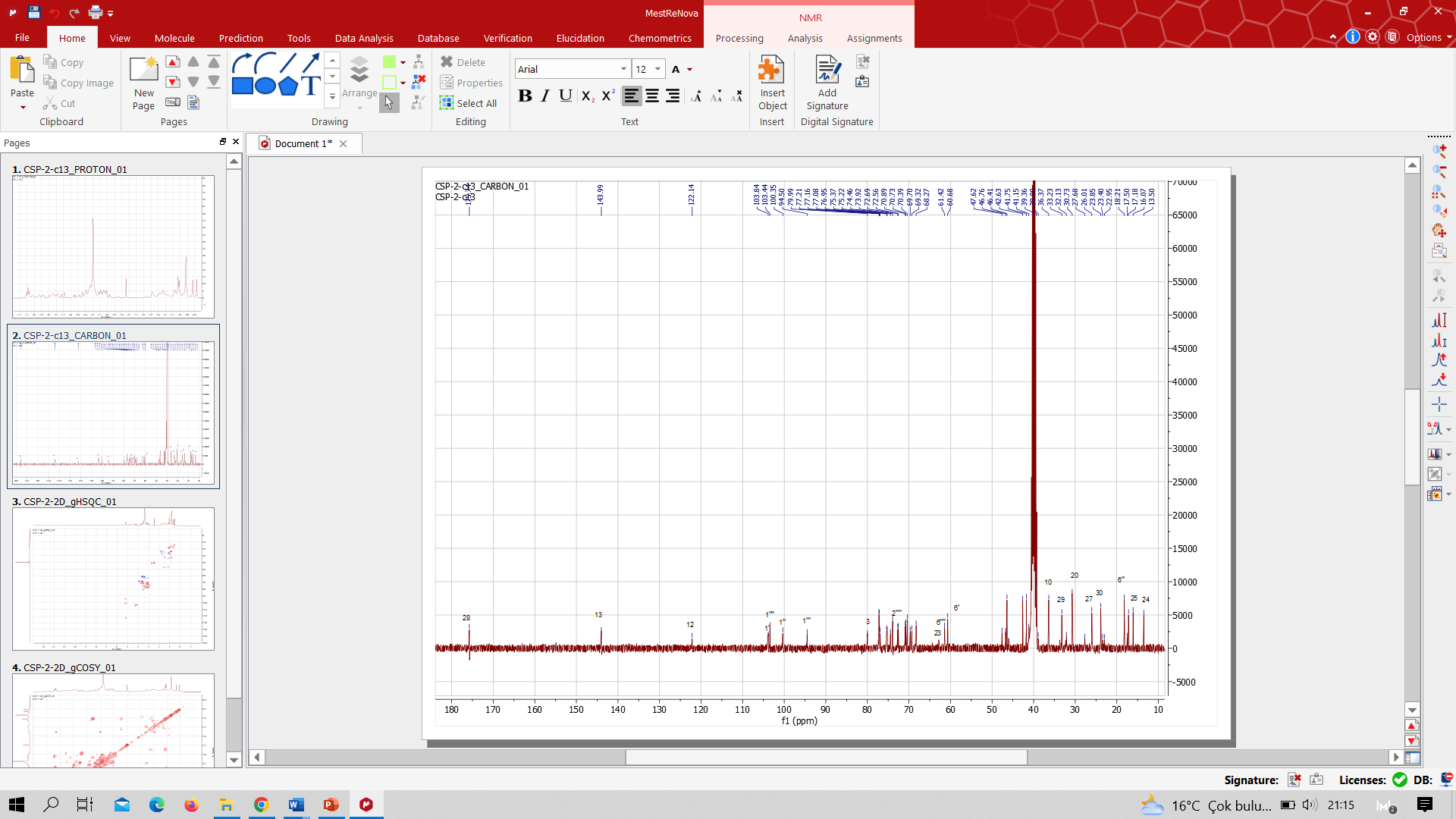


Figure **S *29***. ^13^C NMR spectrum of compound **4** (100 MHz, DMSO-*d_6_*)


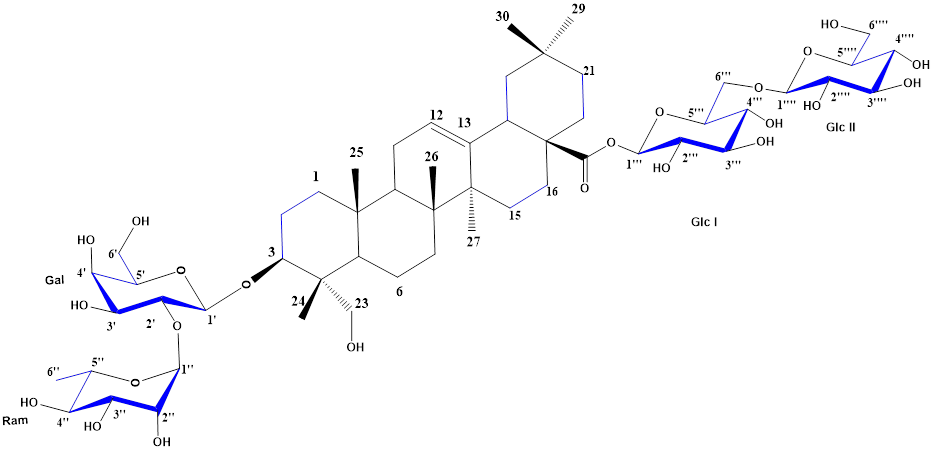

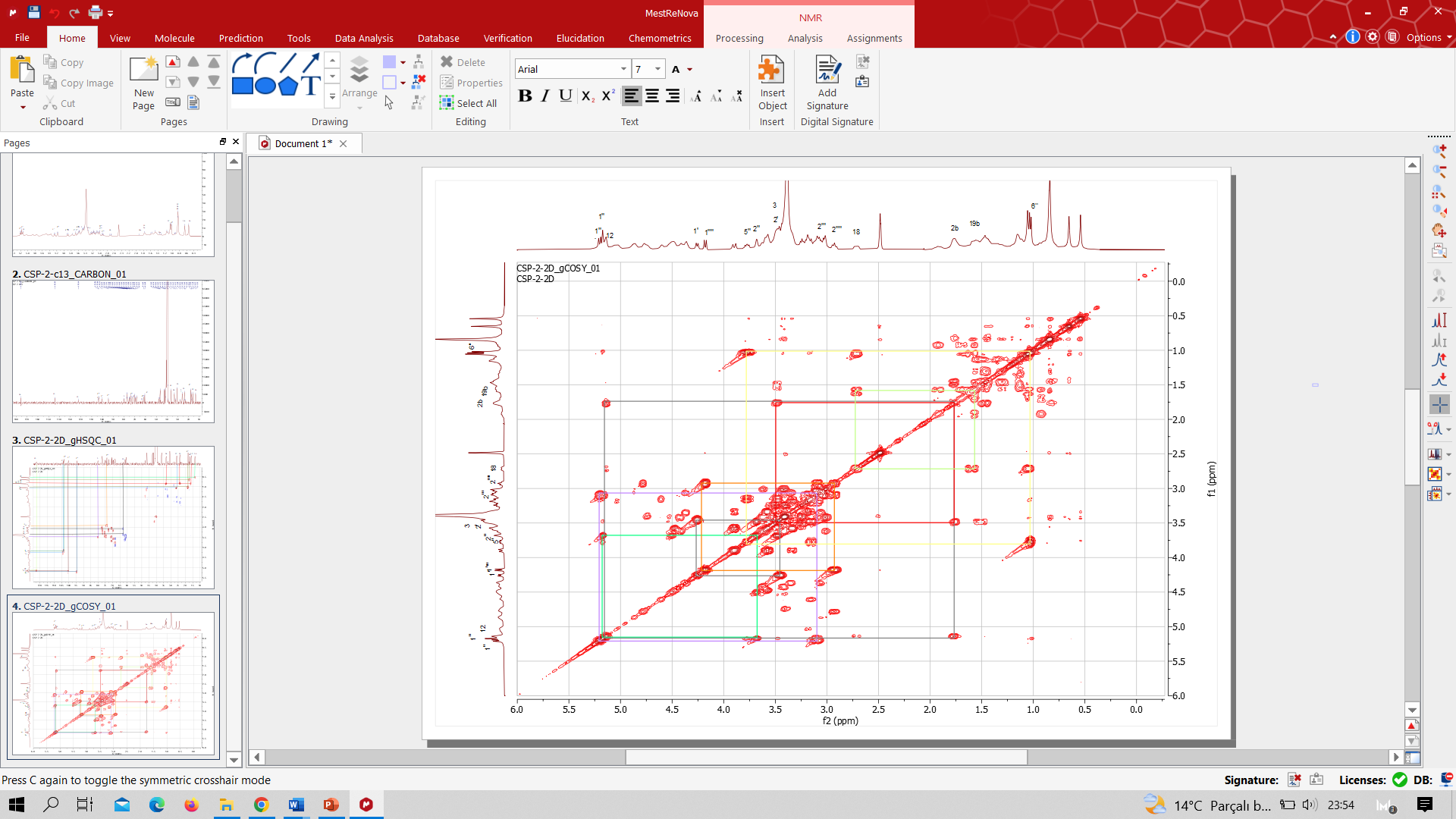


Figure **S *30***. COSY spectrum of compound **4**


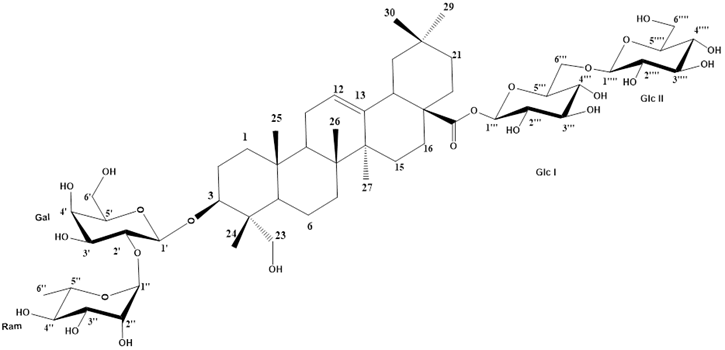

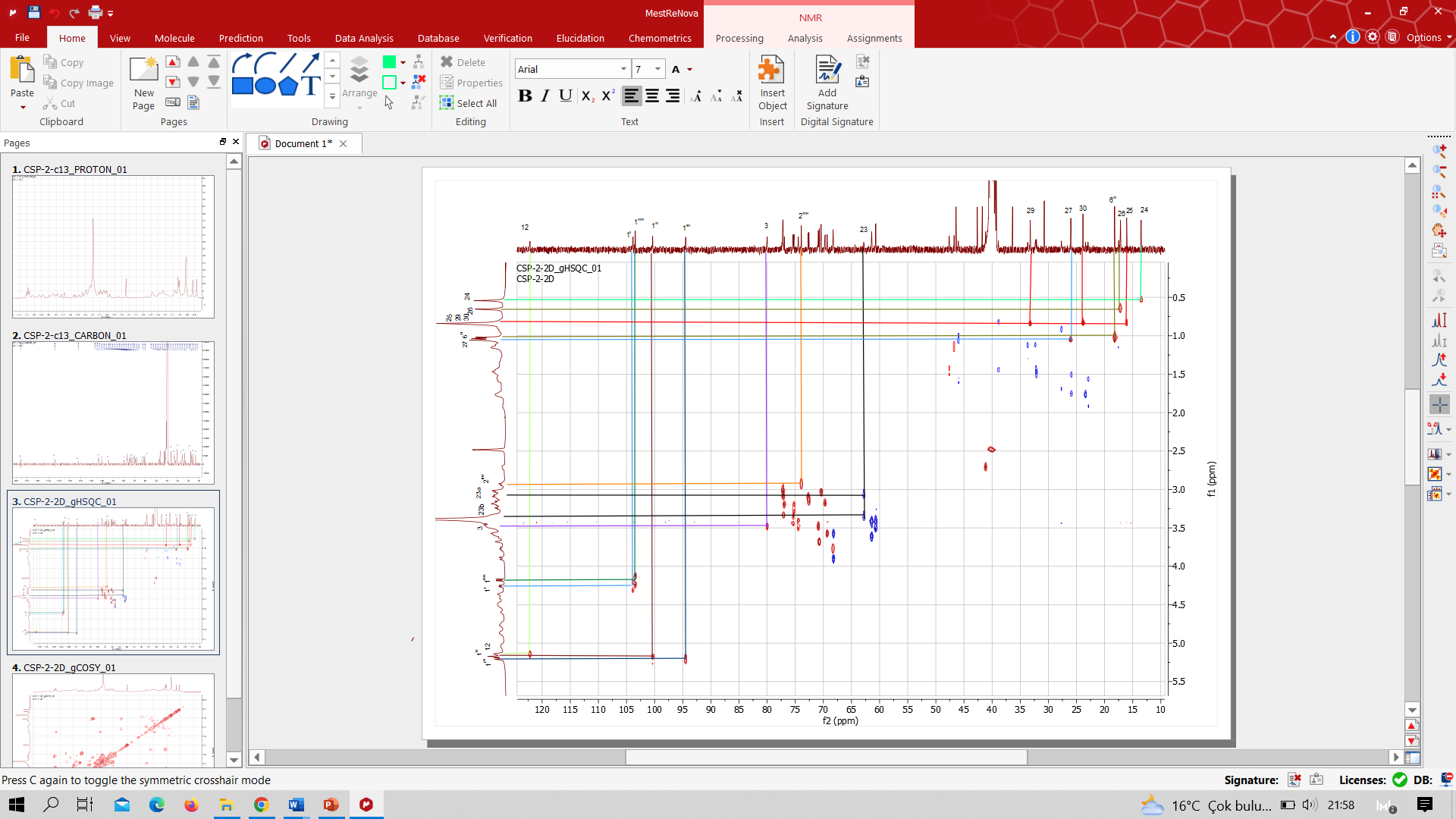


Figure **S *31***. HSQC spectrum of compound **4**


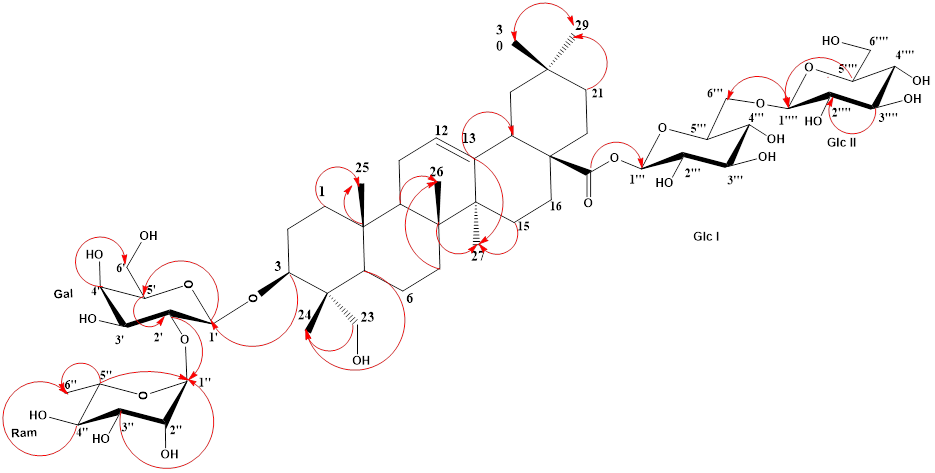

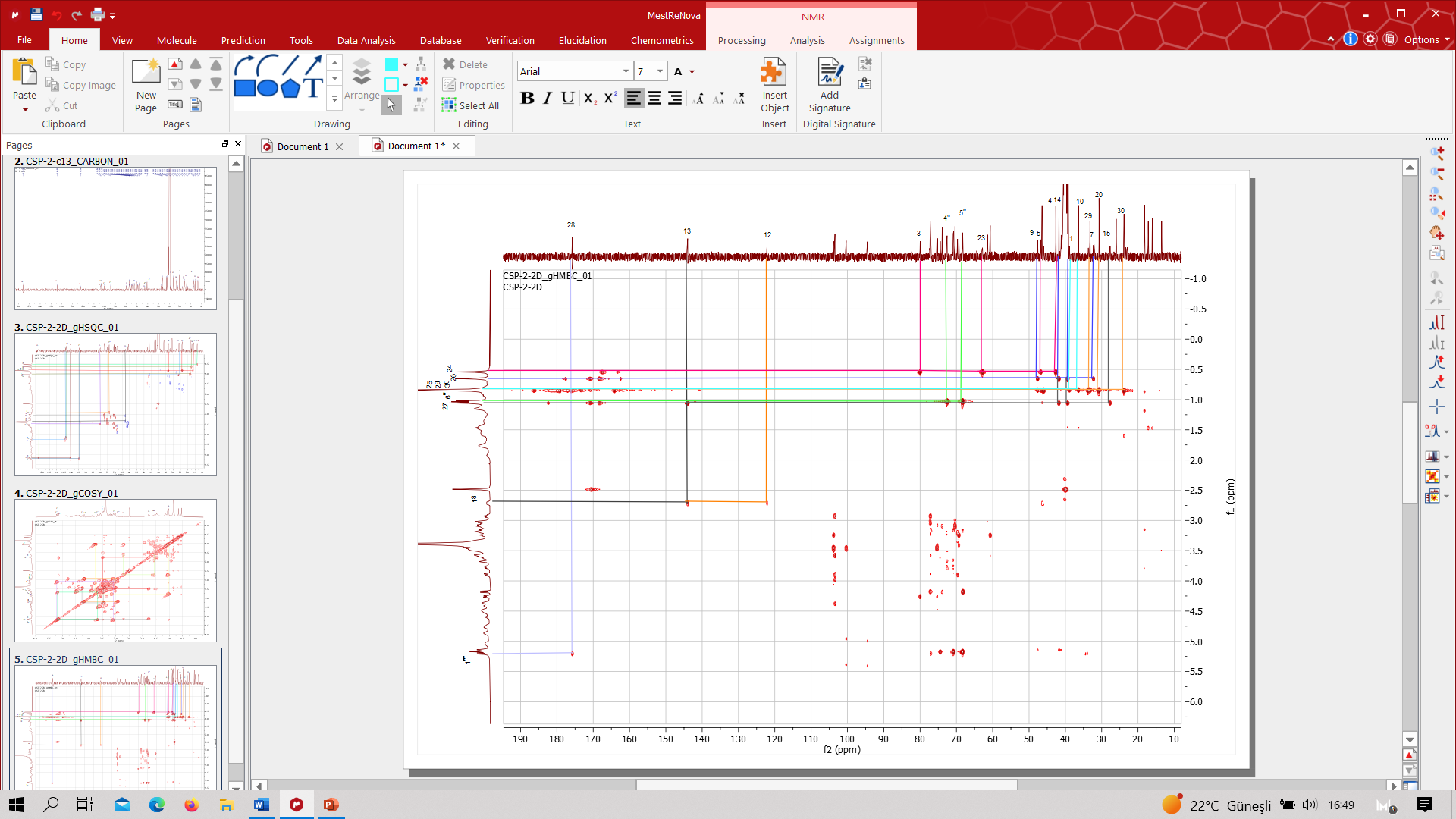


Figure **S *32****.* HMBC spectrum of compound **4**


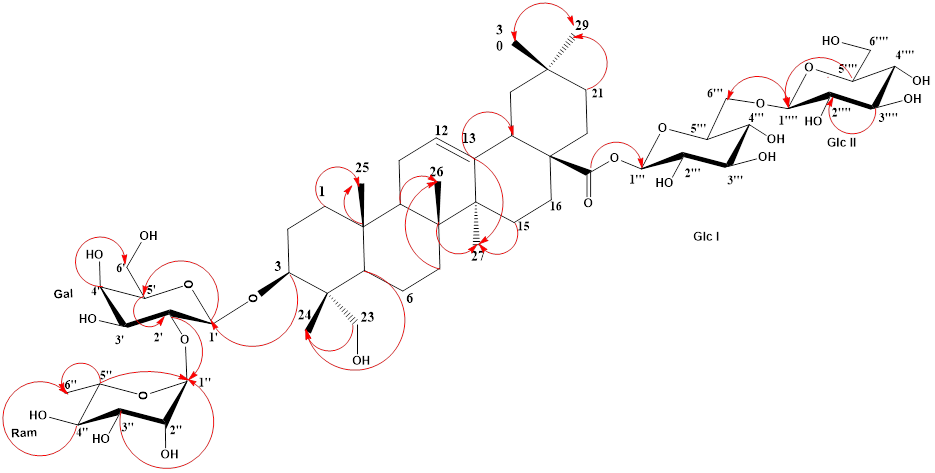

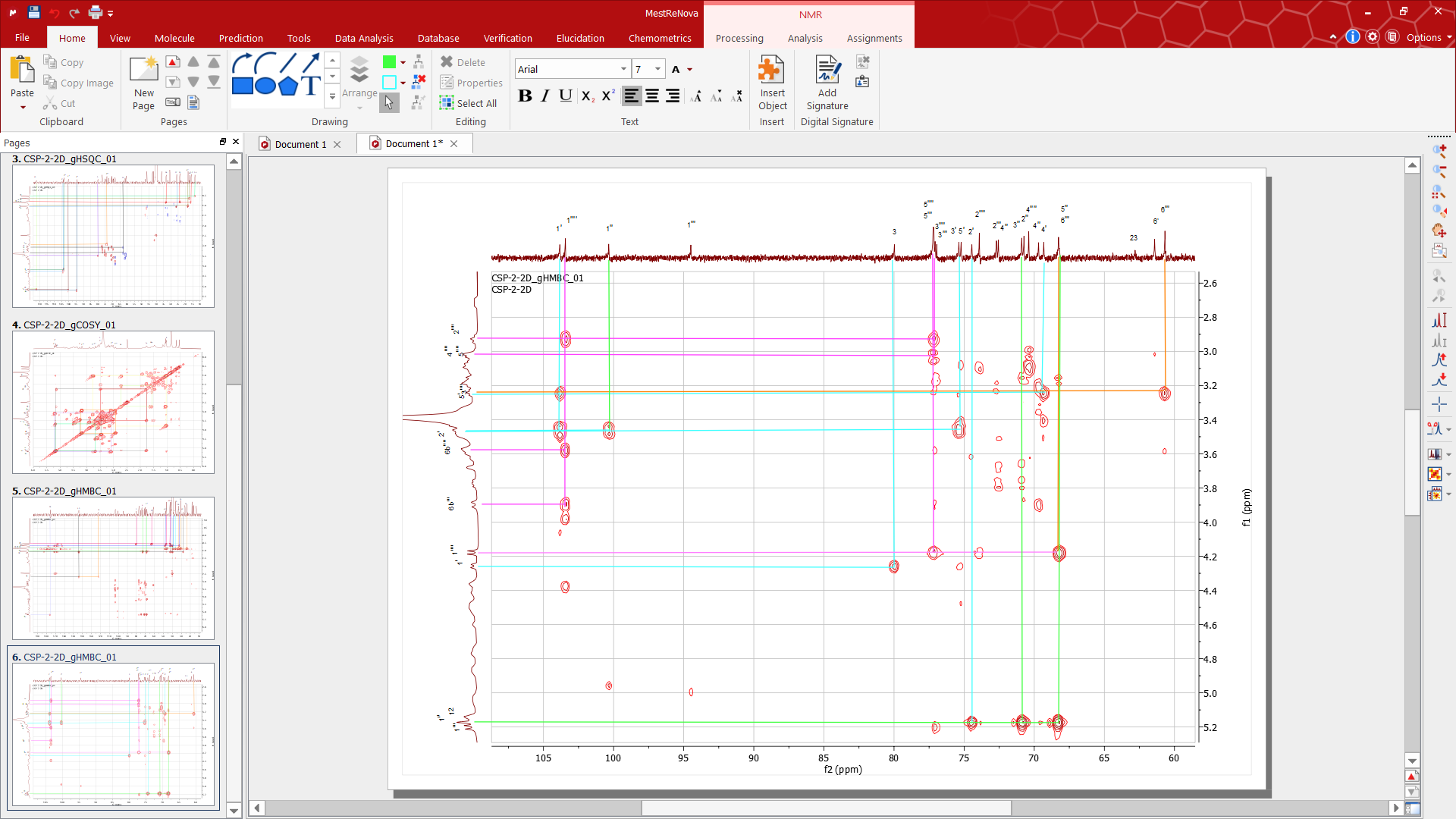


Figure **S *33***. HMBC spectrum of compound **4**

**
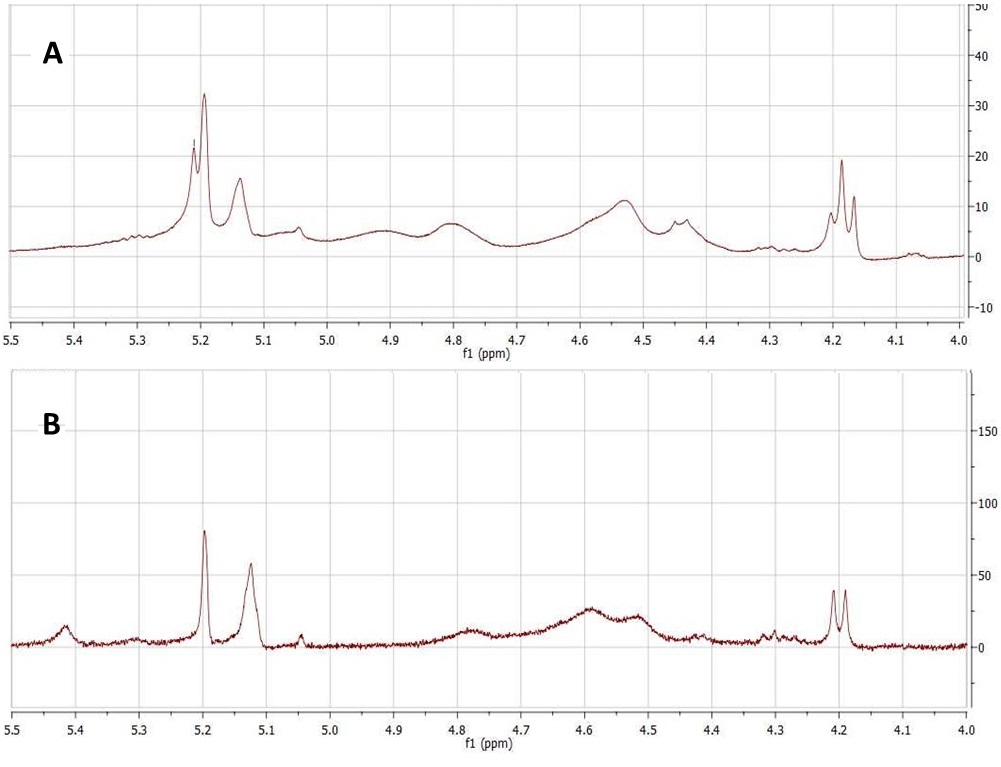
**

Figure **S *34***. Comparison between **A**-^1^H-NMR of compound **2** and **B-** alkaline hydrolysis product compound **2a**

**
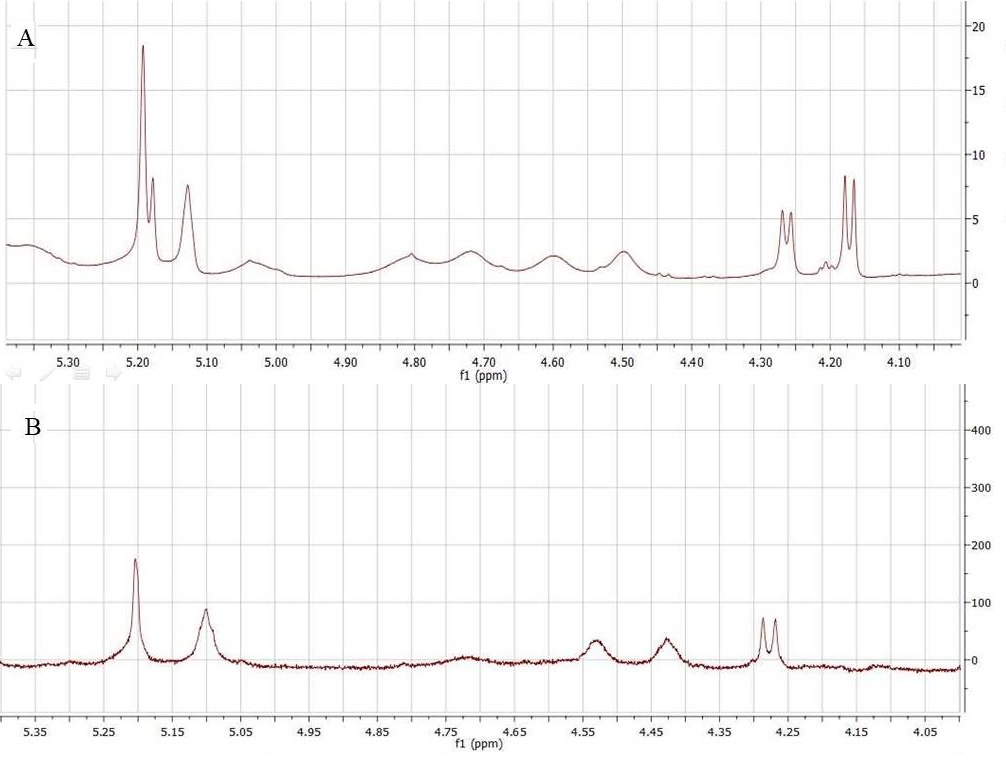
**

Figure **S *35***. Comparison between **A**-^1^H-NMR of compound **3** and **B**- alkaline hydrolysis product compound **3a**


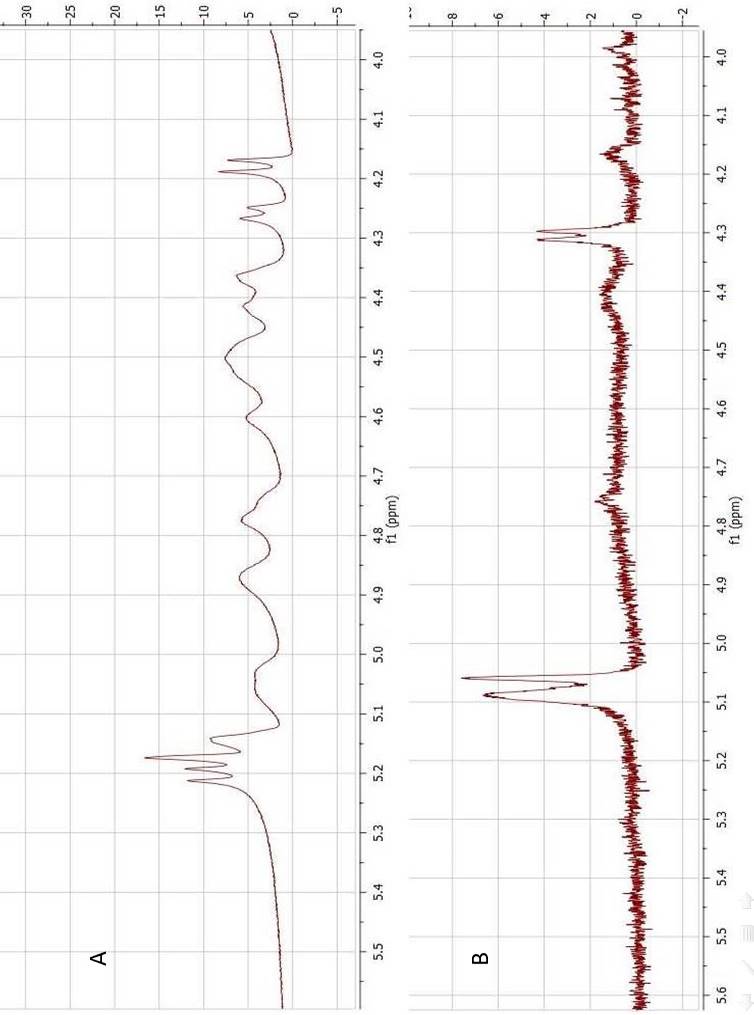


Figure **S *36***. Comparison between **A**-^1^H-NMR of compound **4** and **B**- alkaline hydrolysis product compound **4a**


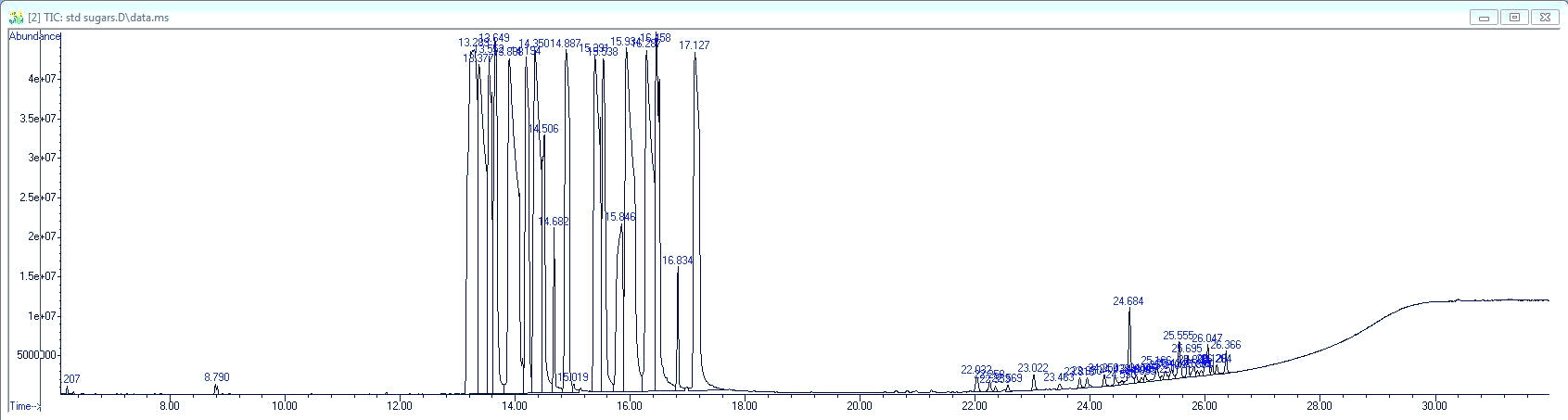


Figure **S *37***. GC-MS spectrum of silylated standard sugars


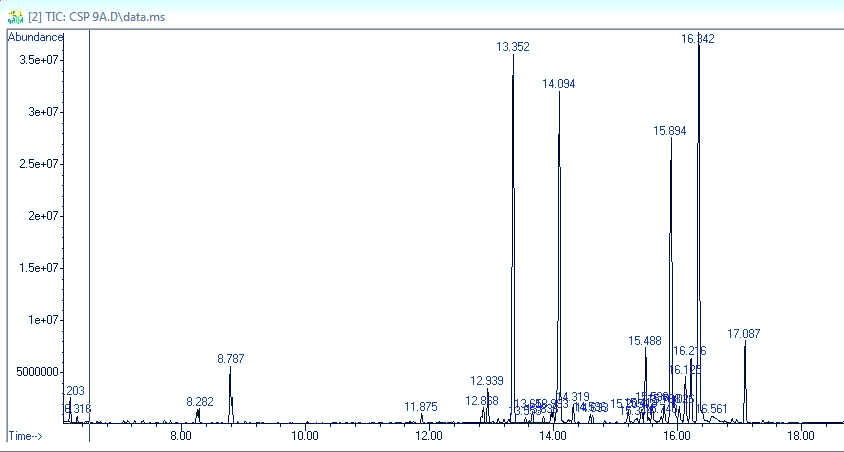


Figure **S *38***. GC-MS spectrum of silylated monosaccharides from compound **1**


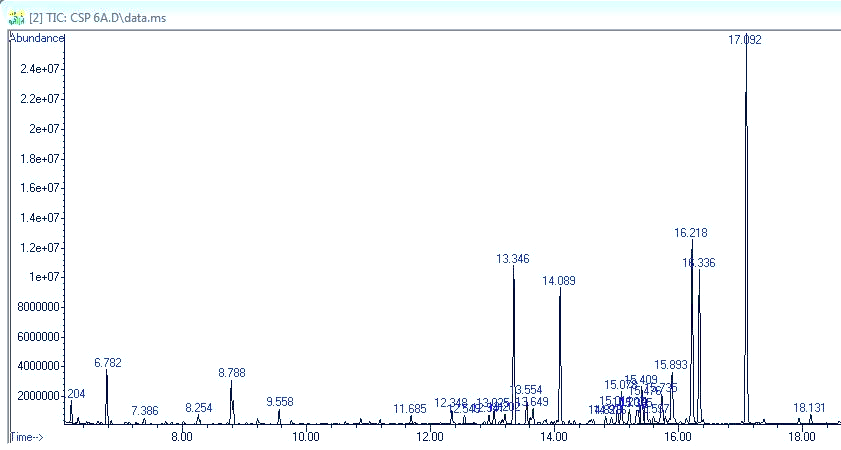


Figure **S *39****.* GC-MS spectrum of silylated monosaccharides from compound **2**


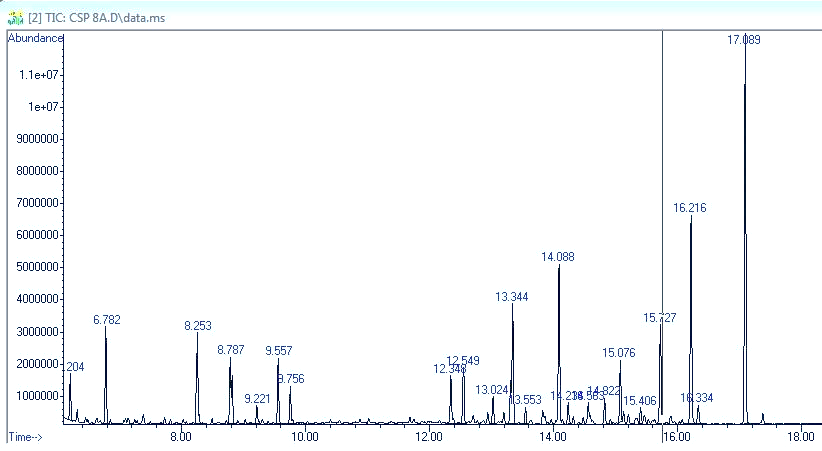


Figure **S *40***. GC-MS spectrum of silylated monosaccharides from compound **3**


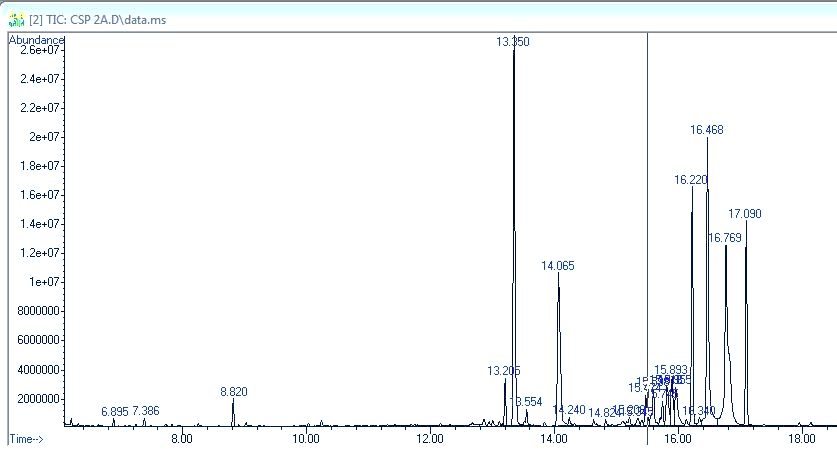


Figure **S *41****.* GC-MS spectrum of silylated monosaccharides from compound **4**

Figure **S *42***. Structure of compound **5** (macranthoidin A)


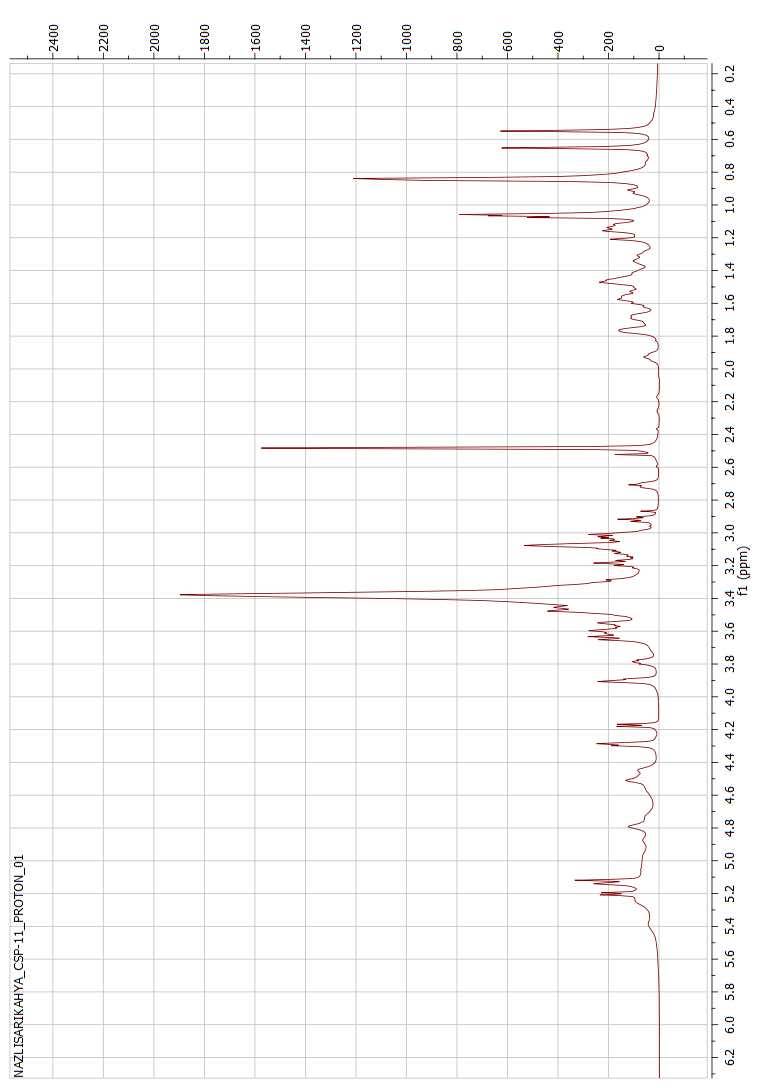


Figure **S *43***. ^1^H NMR spectrum of compound **5** (600 MHz, DMSO-*d_6_*)


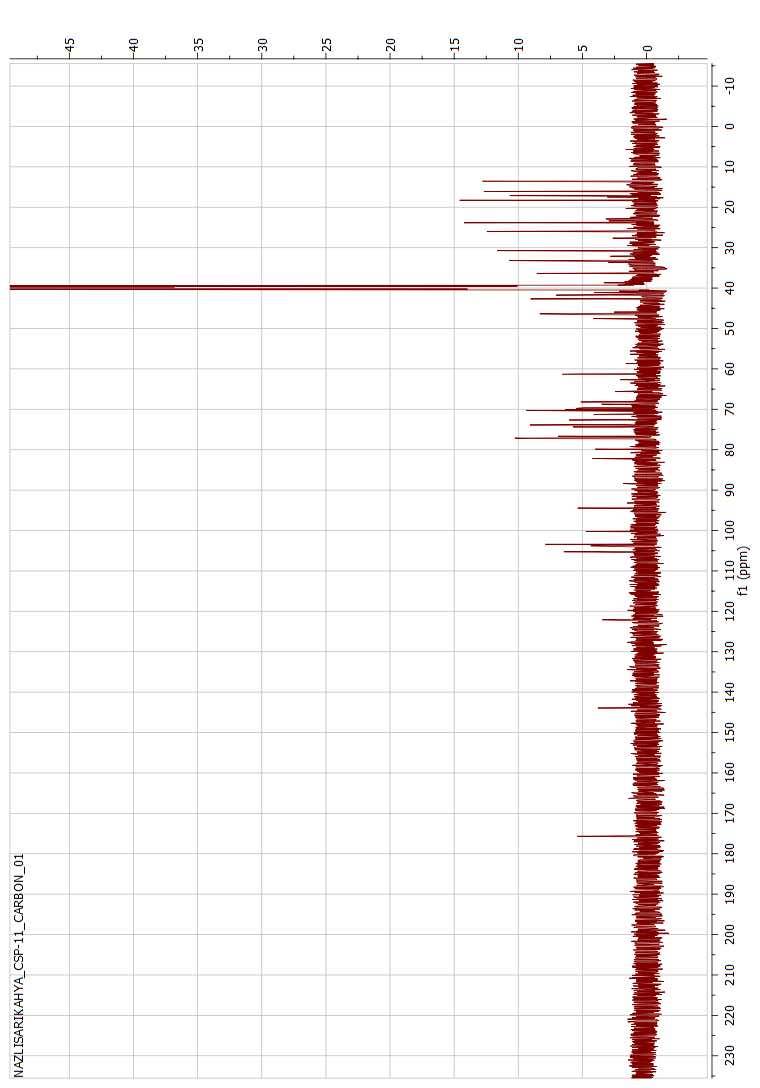


Figure **S *44***. ^13^C NMR spectrum of compound **5** (150 MHz, DMSO-*d_6_*)

Figure **S *45***. Structure of compound **6** (elmalienoside A)


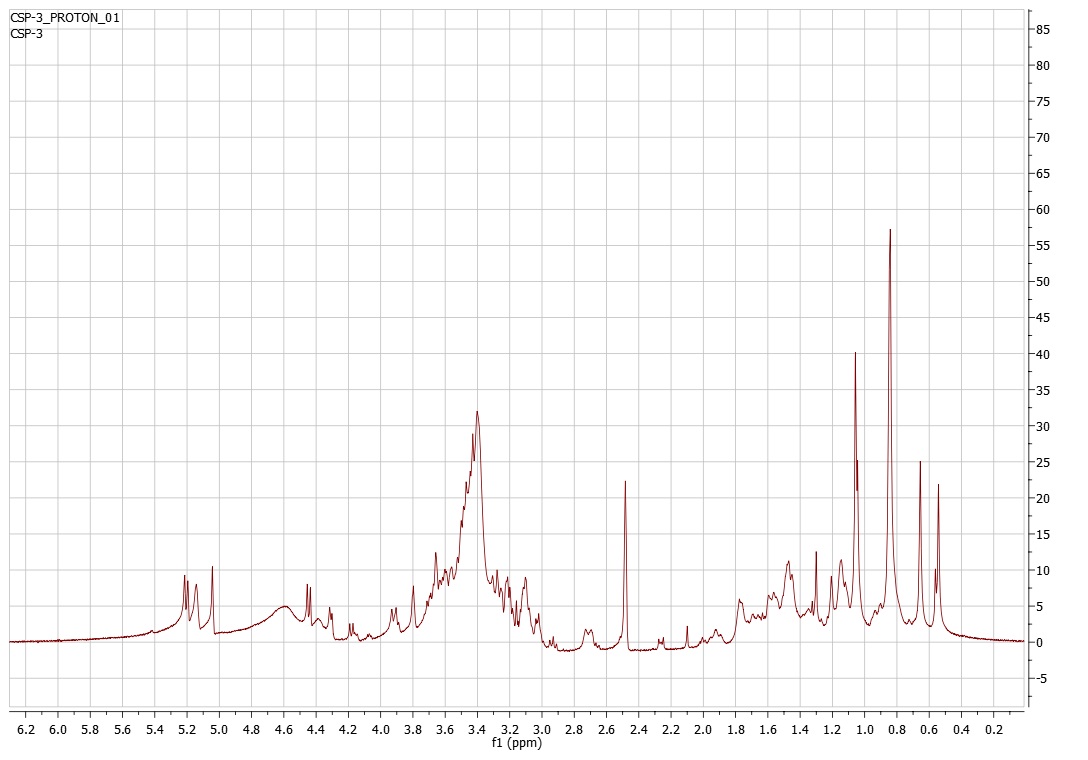


Figure **S *46***. ^1^H NMR spectrum of compound **6** (600 MHz, DMSO-*d_6_*)


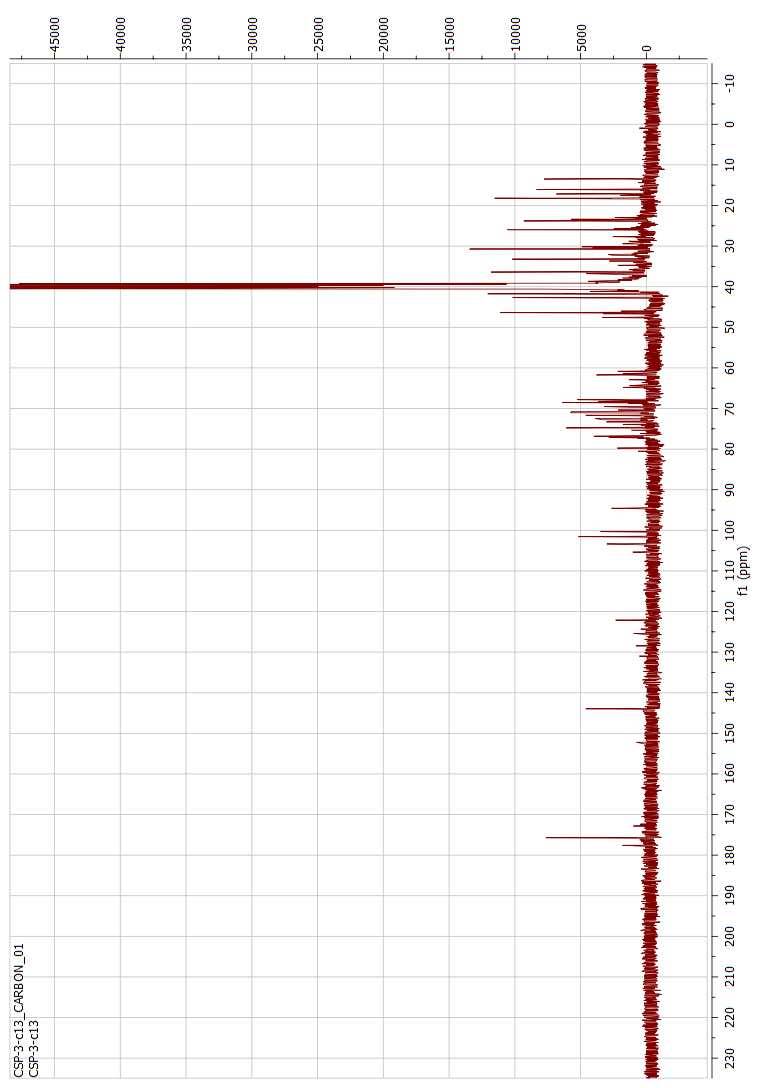


Figure **S *47***. ^13^C NMR spectrum of compound **6** (150 MHz, DMSO-*d_6_*)

Figure **S *48***. Structure of compound **7** (dipsacoside B)


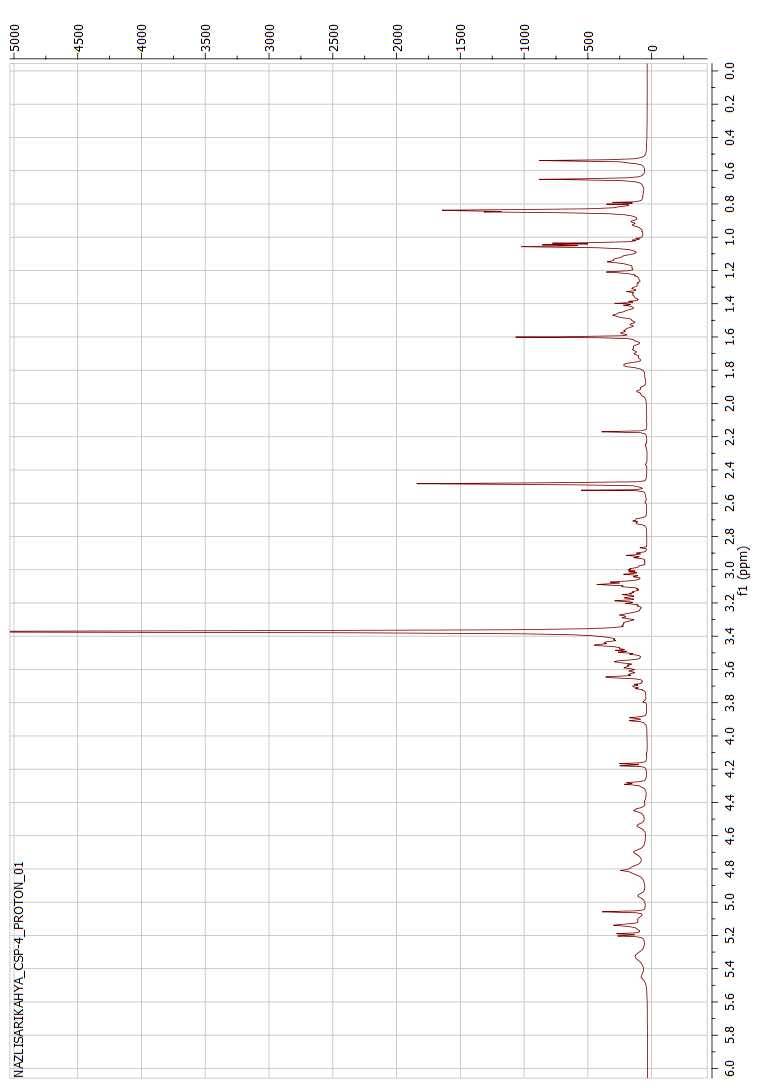


Figure **S *49***. ^1^H NMR spectrum of compound **7** (600 MHz, DMSO-*d_6_*)


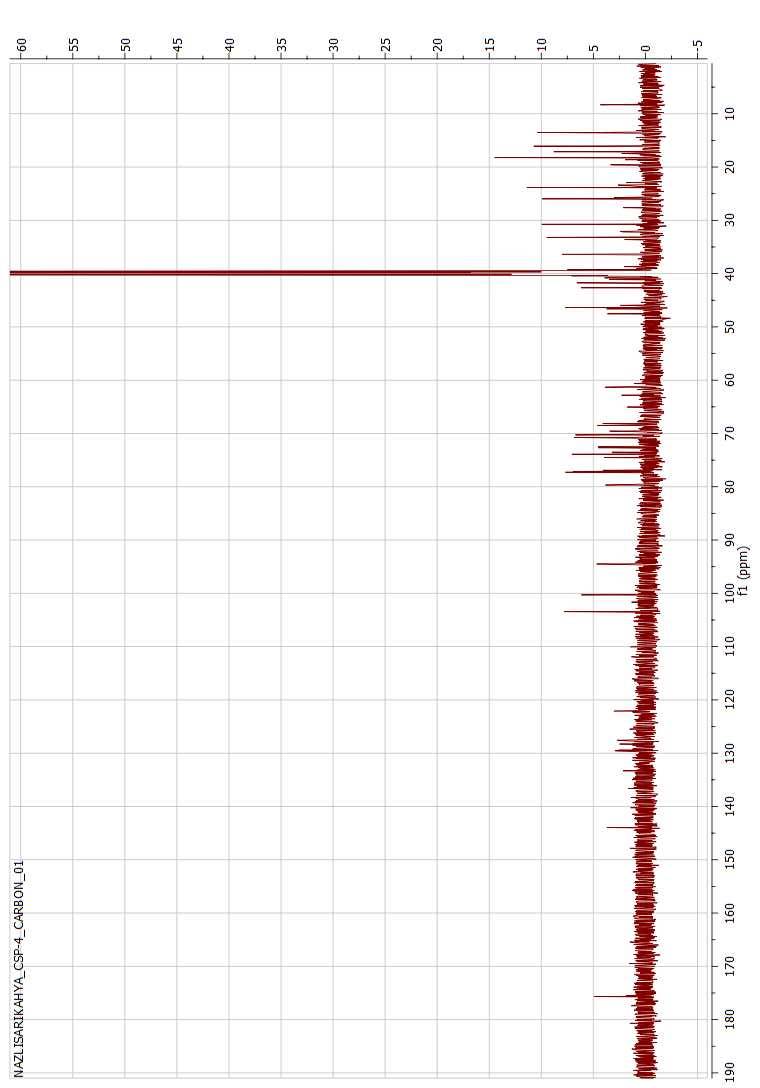


Figure **S *50***. ^13^C NMR spectrum of compound **7** (150 MHz, DMSO-*d_6_*)

Figure **S *51***. Structure of compound **8** (decaisoside E)

**
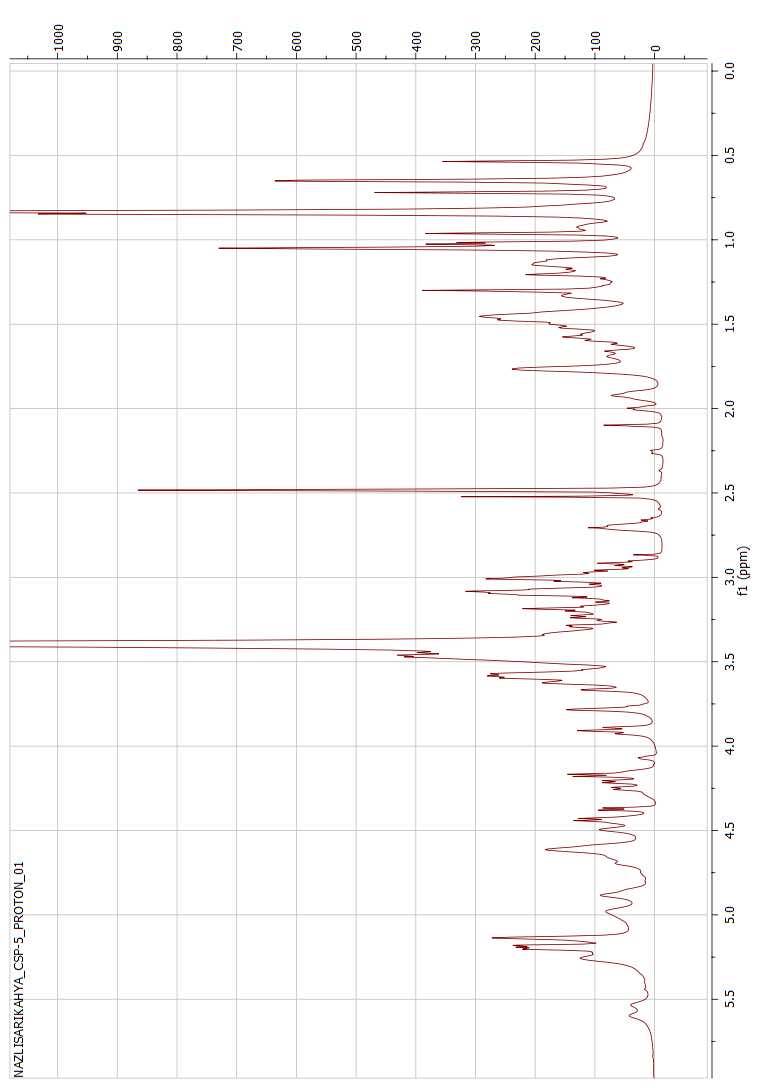
**

Figure **S *52***. ^1^H NMR spectrum of compound **8** (600 MHz, DMSO-*d_6_*)

**
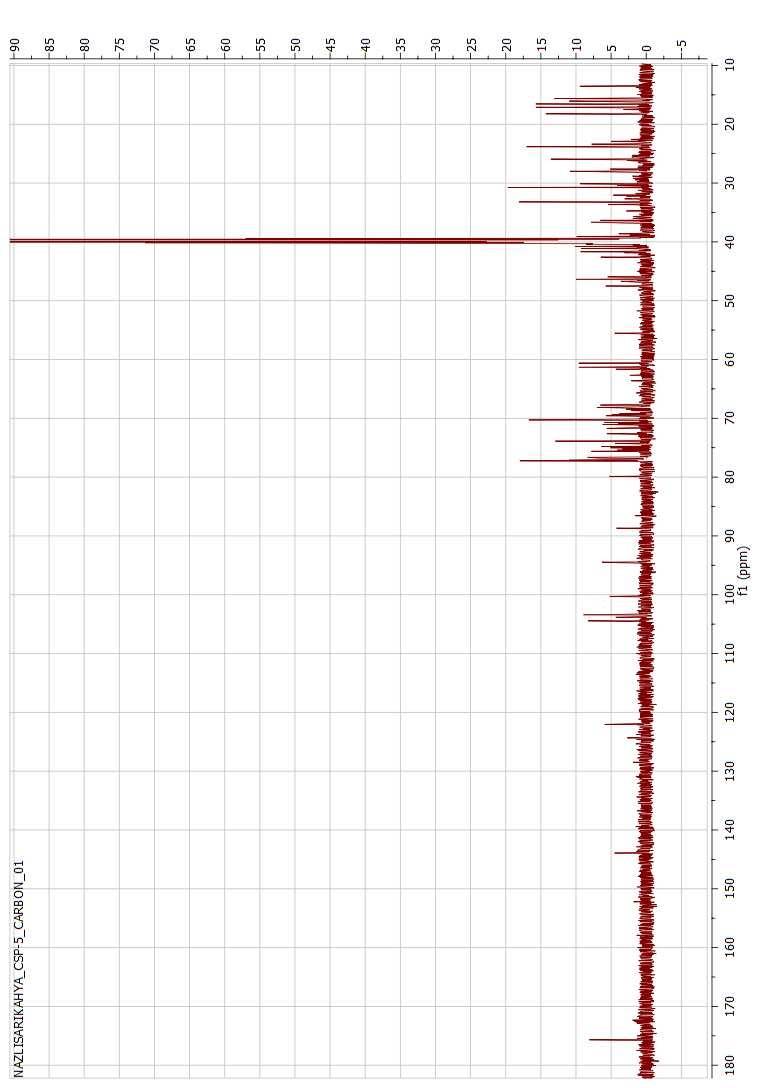
**

Figure **S *53***. ^13^C NMR spectrum of compound **8** (150 MHz, DMSO-*d_6_*)

Figure **S *54***. Structure of compound **9** (scoposide A)

**
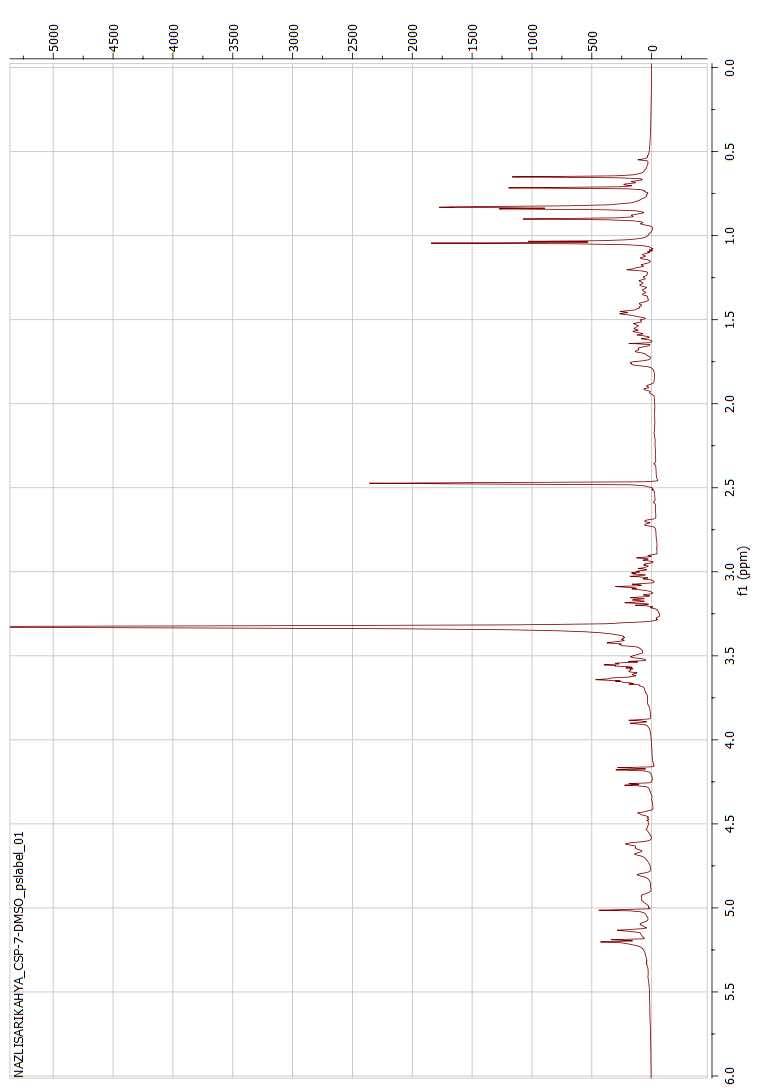
**

Figure **S *55***. ^1^H NMR spectrum of compound **9** (600 MHz, DMSO-*d_6_*)

**
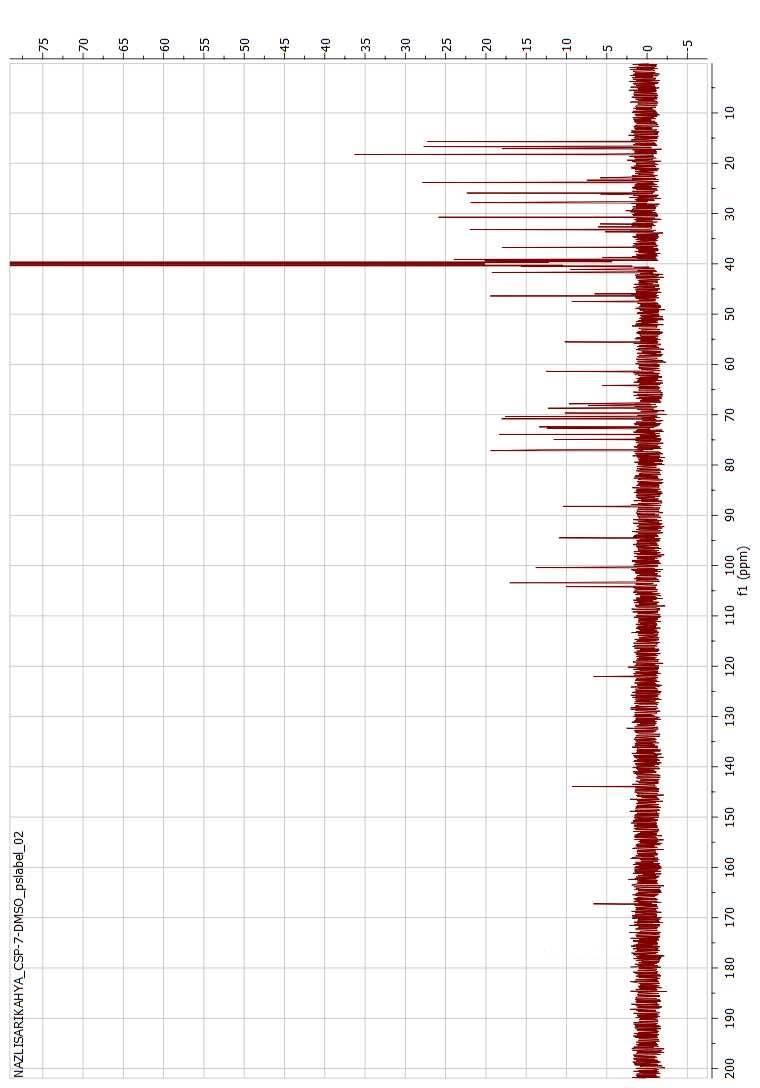
**

Figure **S *56***. ^13^C NMR spectrum of compound **9** (150 MHz, DMSO-*d_6_*)

Figure **S *57***. Structure of compound **10**

**
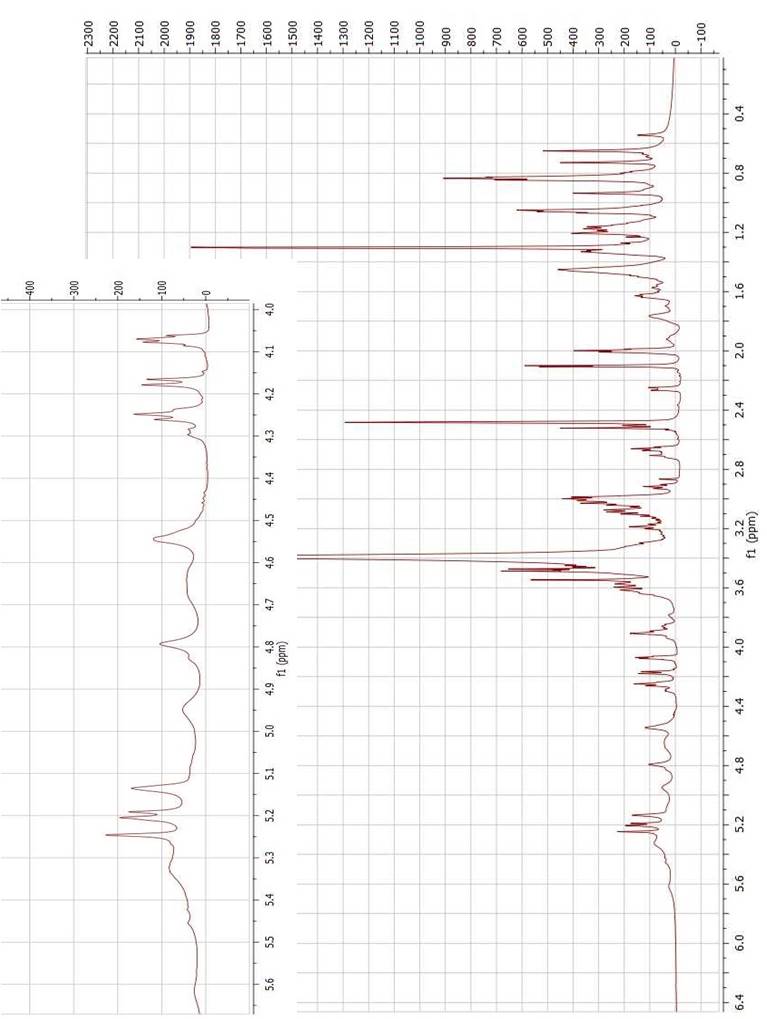
**

Figure **S *58***. ^1^H NMR spectrum of compound **10** (600 MHz, DMSO-*d_6_*)


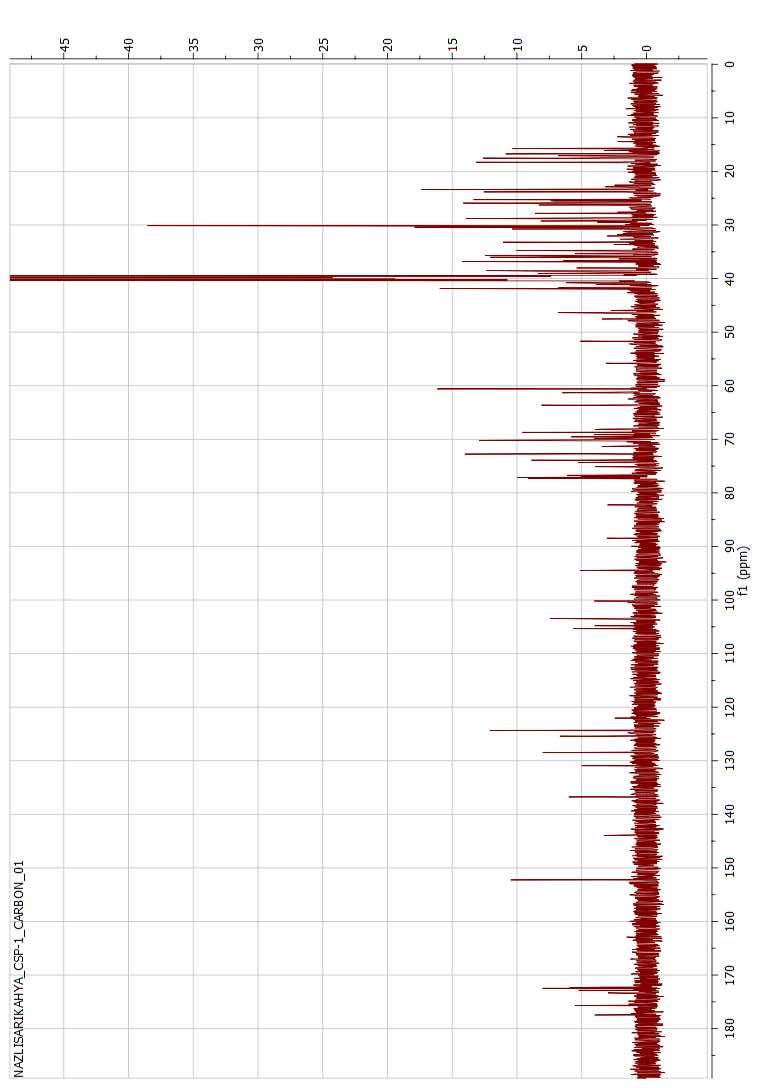


Figure **S *59***. ^13^C NMR spectrum of compound **10** (150 MHz, DMSO-*d_6_*)


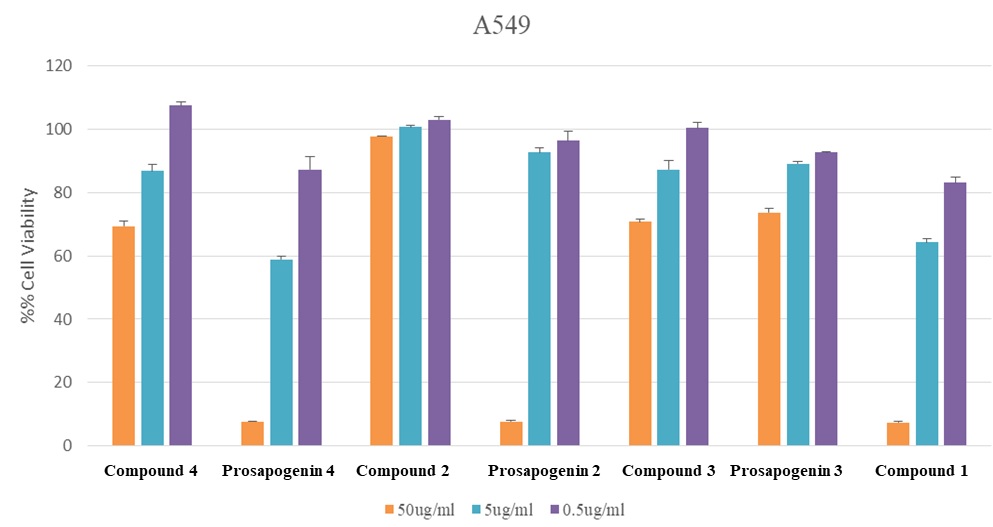


Figure **S *60*.** A549 Cell viability following 48 h treatment of the compound usage.

Each value represents the mean ± standard deviation of three independents measurements.


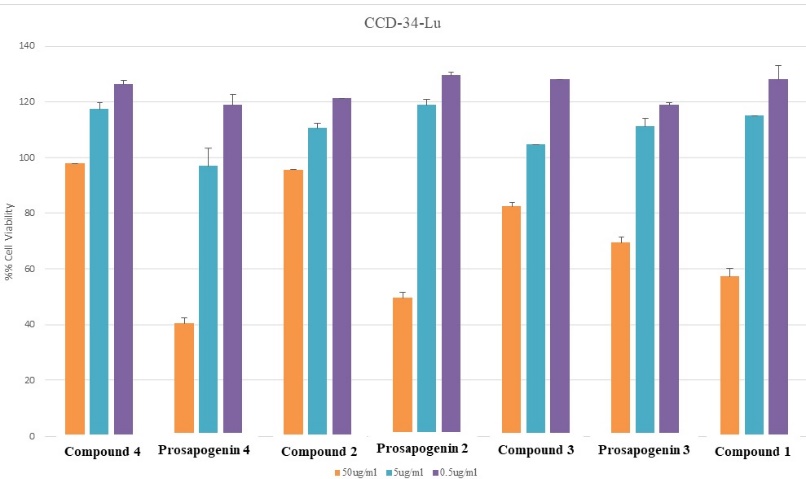


Figure **S *61*.** CCD34-Lu Cell viability following 48 h treatment of the compound usage.

Each value represents the mean ± standard deviation of three independents measurements.


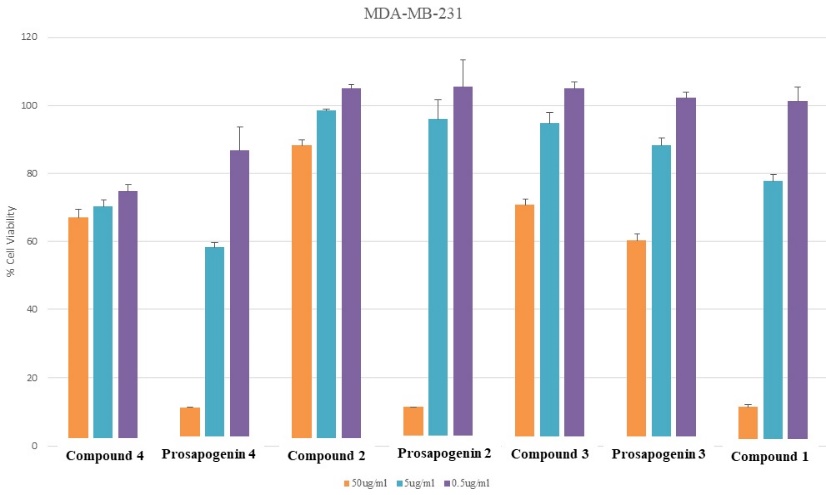


Figure **S *62*.** MDA-MB-231 Cell viability following 48 h treatment of the compound usage.

Each value represents the mean ± standard deviation of three independents measurements.


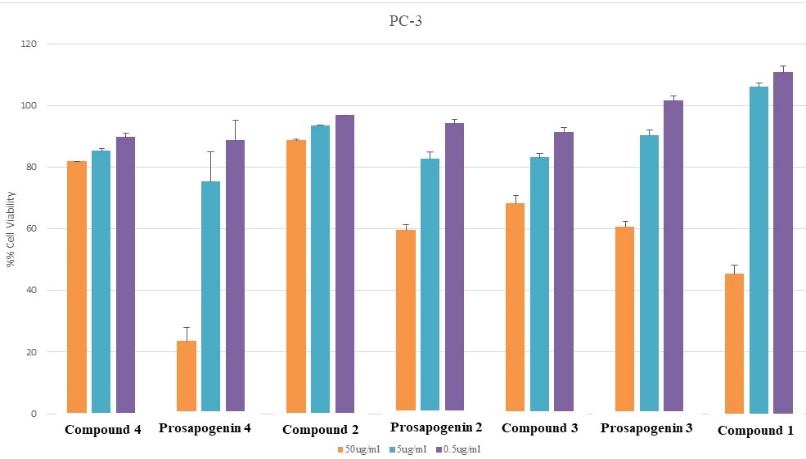


Figure **S *63*.** PC-3 Cell viability following 48 h treatment of the compound usage.

Each value represents the mean ± standard deviation of three independents measurements.


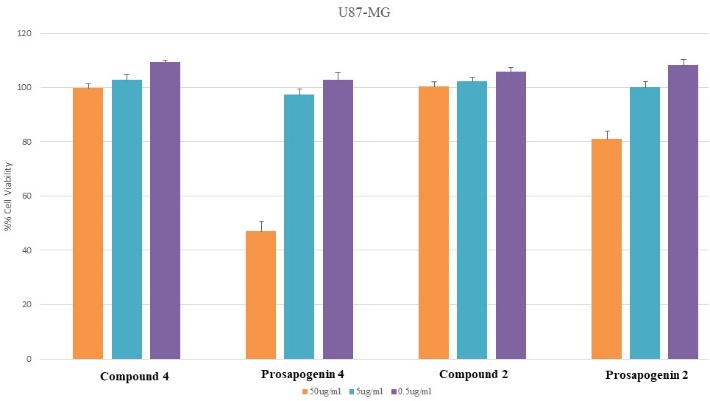


Figure **S *64*** U-87 MG Cell viability following 48 h treatment of the compound usage.

Each value represents the mean ± standard deviation of three independents measurements.


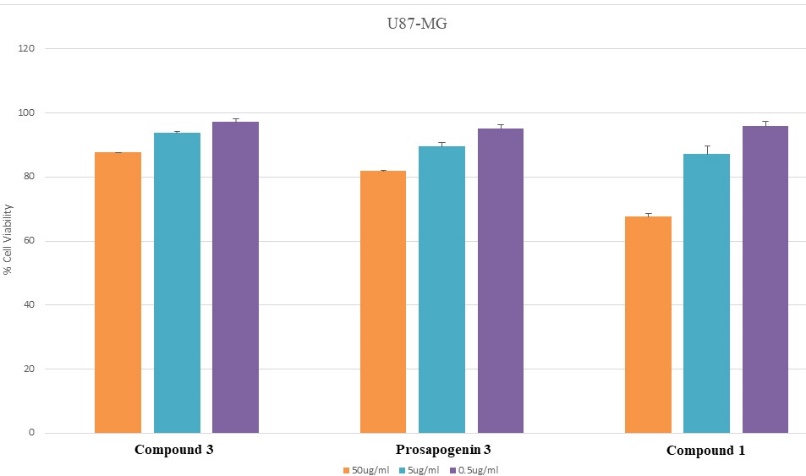


Figure **S** ***65*.** U-87 MG Cell viability following 48 h treatment of the compound usage.

Each value represents the mean ± standard deviation of three independents measurements.


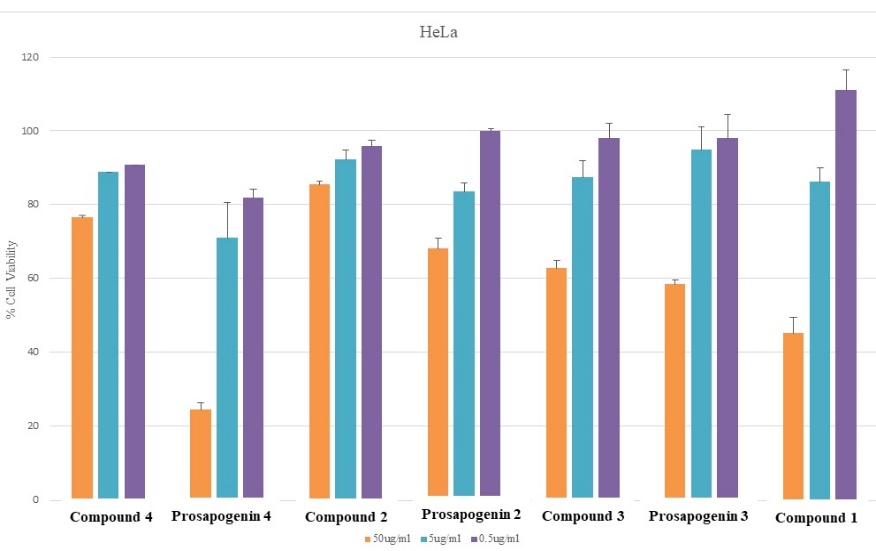


Figure **S *66*.** HeLa Cell viability following 48 h treatment of the compound usage.

Each value represents the mean ± standard deviation of three independents measurements.


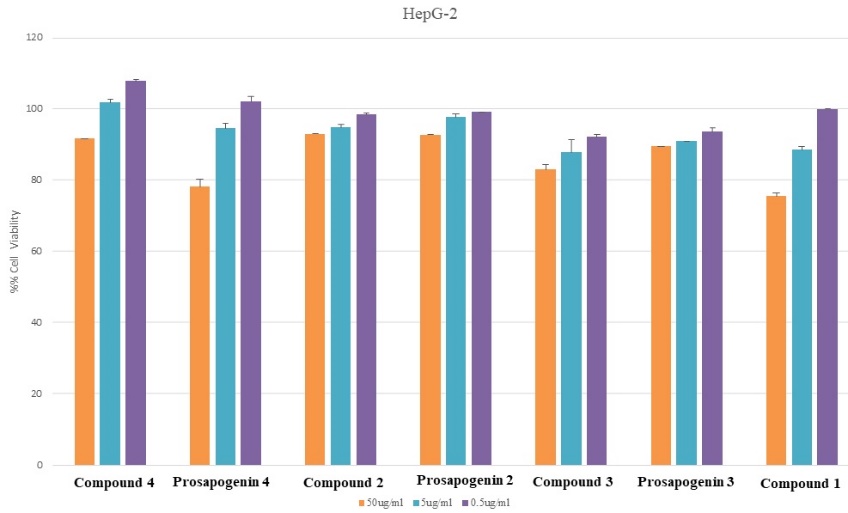


Figure **S *67*.** HepG-2 Cell viability following 48 h treatment of the compound usage.

Each value represents the mean ± standard deviation of three independents measurements.
